# Design, antiproliferative potency, and in silico studies of novel 5-methylfuran-3-yl)thio)-3-phenylquinazolin-4(3H)-one based derivatives as potential EGFR inhibitors

**DOI:** 10.1038/s41598-025-12140-1

**Published:** 2025-07-31

**Authors:** Sara M. Soliman, Adel A.-H. Abdel-Rahman, Eman S. Nossier, Modather F. Hussein, Amr Sabry, Hagar S. El-Hema

**Affiliations:** 1 Chemistry Department, Faculty of Science, Menoufia University, Shebin El-Kom, 32511 Egypt; 2 Department of Pharmaceutical Medicinal Chemistry and Drug Design, Faculty of Pharmacy (Girls), Al-Azhar University, Cairo, 11754 Egypt; 3 The National Committee of Drugs, Academy of Scientific Research and Technology, Cairo, 11516 Egypt; 4 Chemistry Department, College of Science, Jouf University, 72341 Sakaka, Aljouf Saudi Arabia; 5 Department of Pharmaceutical Manufacturing, Faculty of Pharmacy, MUST University, Giza, 3237101 Egypt; 6 Basic Science Department (Chemistry), Thebes Higher Institute for Engineering, Thebes Academy, Maadi, 11434 Egypt

**Keywords:** Quinazolin-4-one, Anticancer, EGFR, Molecular docking, ADMET, Quantum calculations, Organic chemistry, Synthetic chemistry methodology, Biochemistry, Biological techniques, Cancer, Cell biology, Chemical biology, Computational biology and bioinformatics, Drug discovery, Chemistry

## Abstract

Quinazolinone derivatives have been broadly studied as anti-cancer drug candidates due to their potential to inhibit key signaling pathways involved in tumor progression. In the current study, new 2-[(4-substituted-5-methylfuran-3-yl)thio]-3-phenylquinazolin-4(3*H*)-one derivatives (**2**–**10**) were designed and assessed for anti-cancer activity. Cytotoxicity of the compounds was tested against normal WI-38 cells and cancer cell lines HepG-2 (liver), MCF-7 (breast), and HCT-116 (colorectal). In addition, their inhibitory effects on EGFR and VEGFR-2, key targets for tumor growth and angiogenesis, were assessed. Compounds **6b** and **10** showed significant cytotoxic activity, with** 6b** (IC₅₀ = 0.19 ± 0.03 μM) being the most effective EGFR inhibitor, over **10** (IC₅₀ = 0.51 ± 0.04 μM) and as potent as erlotinib (IC₅₀ = 0.23 ± 0.02 μM). Flow cytometry revealed that **6b** induced apoptosis in 35.29% of MCF-7 cells and G₂/M phase cell cycle arrest, much better than that of untreated cells (6.81%). In silico ADMET prediction and molecular docking confirmed high EGFR binding affinity and favorable pharmacokinetic properties. Overall, compound **6b** showed promising anti-cancer activity via EGFR inhibition, apoptosis, and cell cycle arrest and is a good lead for further development as an EGFR-targeted agent.

## Introduction

With an estimated 20 million new cases and 10 million deaths from the disease each year, the burden of cancer is substantial and continually growing. This burden is predicted to double over the next 20 years, with the largest increases anticipated in low- and middle-income countries, where up to 30 million new cases may be reported by 2040^1–3^. Hormone therapy, photodynamic therapy, radiation therapy, immunotherapy, chemotherapy, stem cell transplantation, and surgery are among the many clinical therapeutic options that are available^4–8^. One important factor in reducing the severity of side effects is the degree of selectivity of the drugs employed in chemotherapy. The treatment of many cancer types has greatly benefited from the creation of novel medications with multiple modes of action. It is known that a multitargeted agent may be more therapeutically beneficial and have a lower profile of side effects than a monotargeted medication. Based on encouraging preclinical and clinical results, multitargeted agent therapeutic methods seem to be the next big development in cancer treatment^9–14^.

Cell signal transduction, which regulates cell migration, differentiation, proliferation, and survival, depends on the tyrosine kinase family member known as the epidermal growth factor receptor (EGFR)^15–17^. These days, a sizable fraction of human cancer cells overexpress EGFR. As a result, the EGFR protein has become considered an essential targeted treatment for cancer^18–25^. Furthermore, one of the characteristics of cancer cells that should be considered in the development of effective and safe anticancer medications is growth factor secretion.

Vascular endothelial growth factors (VEGFs), which are necessary for angiogenesis, are one of these. Consequently, it has been demonstrated that angiogenesis restriction is a successful cancer therapeutic approach^26–29^. Numerous malignancies, such as colorectal^32^, breast^31^, urothelial^33^, and lung nonsmall cell^30^ carcinomas, have been linked to the VEGFR-2 signaling pathway. These results highlighted the importance of EGFR and VEGFR-2 inhibition as a cancer treatment strategy. One important element in medicinal chemistry is quinazoline, an example of a heterocyclic scaffold containing nitrogen^34–40^. The beneficial biological activities of quinazoline heterocyclic compounds are numerous and include anti-inflammatory^41^, carbonic anhydrase inhibition^42–44^, anti-cancer action^45–51^, antimalarial^52^, antidiabetic^53^, antimicrobial^54–56^, antituberculosis^57^, anti-coronavirus^58^, and antioxidant activity^59^.

**Fig. 1:**
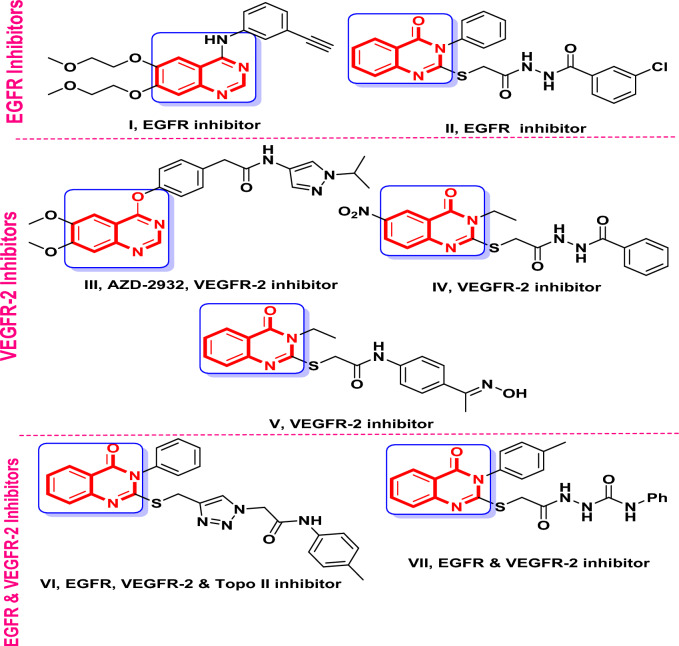
Potential cytotoxic agents bearing quinazolinone moiety that target EGFR and/or VEGFR-2 inhibitory pathways.

Quinazoline-based anticancer drugs include the potent VEGFR-2 inhibitor AZD-2932, **III**^60,61^ and the FDA-approved erlotinib **I** with EGFR inhibitory potency (Fig. 1). The *N*3-phenyl-quinazoline-4(3*H*)-one scaffold’s compound **II**, which has an S-alkylated substituent at position 2, shown encouraging antiproliferative and EGFR inhibitory properties, according to Fang et al.^62^. Significant cytotoxic activity was demonstrated by VEGFR-2 suppression in other *N*3-ethyl-quinazoline-4(3*H*)-one **IV** and **V** bearing S-alkylated substituent at position 2^63,64^. Derivatives **VI** and **VII** having an S-alkylated substituent at *p*-2 of *N*3-phenyl or *N*3-tolyl-quinazoline-4(3*H*)-one, respectively, demonstrated dual EGFR/VEGFR-2 inhibitory action^65,66^.

**Fig. 2:**
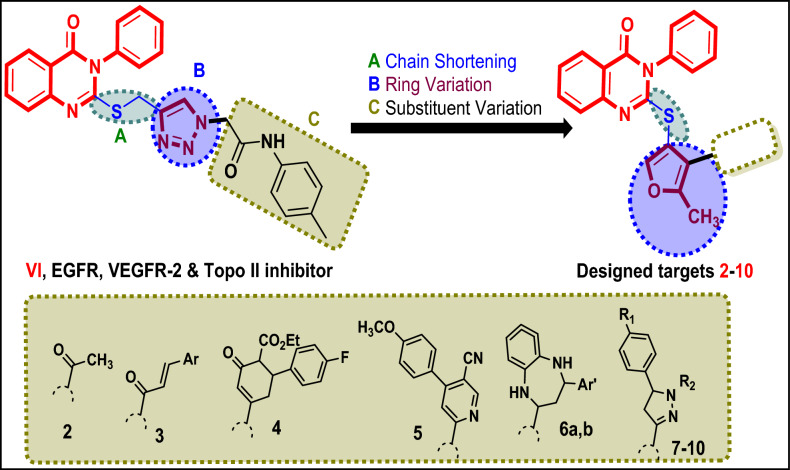
Design of new *N3*-phenyl-quinazoline-4(3*H*)-one candidates **2**–**10** with different *S*-alkylated substituent at p-2 targeting EGFR and VEGFR-2 kinases.

Using various drug design strategies, including maintaining the quinazolin 4(3*H*)-one core, shortening the thiomethyl linker at p-2 to thio one, ring variation of 1,2,3-triazole to the 5-methyfuran, and substituent variation of *p*-tolyl)acetamide with different fragments, novel quinazolin-4(3*H*)-ones **2**–**10** were designed as inhibitors of EGFR and VEGFR-2-TKs with regard to lead **VI**^65^. These findings were based on our earlier research for the development of new targeted anti-cancer agents^67–71^ (Fig. 2).

The produced compounds were tested in vitro against colorectal HCT-116 cancer cell lines, breast MCF-7, human liver HepG-2, and normal diploid cell line WI-38. Using the assessment of Bax, Bcl-2, and p53 levels, the potential quinazolin-4(3*H*)-ones were assessed for their capacity to inhibit EGFR and VEGFR-2 kinases and trigger apoptosis in the MCF-7 cell lines. In order to forecast the binding patterns and mechanism of action that underlie the potent quinazolin-4(3*H*)-ones’ cytotoxic effect, they underwent in silico ADMET elucidation and docking simulation with their targets.

## Results and discussion

### Chemistry

Since the design of quinazoline derivatives showed a wide range of biological potential, especially in the field of anti-cancer therapy, we were motivated to concentrate our efforts on their synthesis. The objective of this work was to synthesize a number of heterocyclic compounds derived from the thioxodihydro quinazoline derivative.

Given that, phenyl isothiocyanate and anthranilic acid combine in 1,4-dioxane to produce phenyl-2-thioxo-2,3-dihydroquinazolin-4(1*H*)-one, the latter product was created by the intermediate synthesis of the thiourea derivative, which was then employed to synthesize various heterocyclic compounds by reacting with chloroacetyl chloride to produce derivative **1** of chloroethanethioate^72^.

**Scheme 1:**
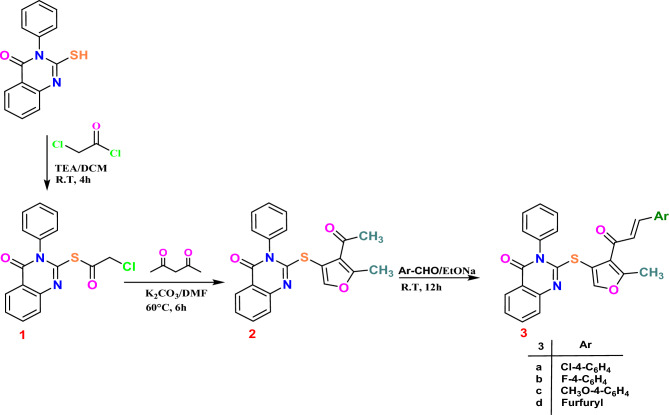
Synthesis of quinazolinone-based derivatives **1**–**3a**-**d**.

Acetyl-5-methylfuran derivative **2**^73,74^ was produced via the reaction of **1** with acetylacetone. This derivative could react with various aldehyde compounds to yield chalcone derivatives. Accordingly, compound **2** produced the chalcone derivatives **3a**–**d** when it reacted with any of the following: *p*-chlorobenzaldehyde, *p*-fluorobenzaldehyde, *p*-methoxybenzaldehyde, and furfuraldehyde (Scheme 1). The structures of Compounds **1**, **2**, and **3a**–**d** were elucidated based on their spectral and analytical data.

For compound **1**, the IR spectrum showed characteristic absorption bands corresponding to aromatic C–H stretching at 3081 and 3026 cm⁻^1^, aliphatic C–H stretching at 2936 cm⁻^1^, two distinct carbonyl groups at 1710 cm⁻^1^ for Acyl chloride and at 1659 cm⁻^1^ for quinazolin-4-one, an imine group at 1594 cm⁻^1^, and aromatic C=C stretching at 1495 cm⁻^1^ (Supplementary Figure S**1**). The ^1^H NMR spectrum exhibited a singlet at δ 4.22 ppm, indicating the presence of methylene (CH₂) protons, along with multiplets between δ 7.56–8.08 ppm corresponding to nine aromatic protons from two phenyl rings (Supplementary Figure S**2**). In the ^13^C NMR spectrum, a signal at δ 49.33 ppm was attributed to the CH₂ carbon, while multiple signals between δ 115.71 and 139.58 ppm were assigned to aromatic carbons of the two phenyl groups. A peak at δ 147.12 ppm was assigned to the pyrimidinone carbon bonded to a nitrogen atom, while the imine carbon appeared at δ 159.78 ppm. Finally, two downfield signals at δ 166.68 and 176.02 ppm confirmed the presence of the two carbonyl carbons of quinazolin-4-one and acyl chloride (Supplementary Figure S3).

The IR spectrum of compound **2** exhibited a characteristic absorption band at 1722 cm⁻^1^, confirming the presence of a new acetyl carbonyl group. The absence of the band at 1710 cm⁻^1^, which corresponds to the acyl chloride, further verified the formation of the desired compound (Supplementary Figure S4). The ^1^H NMR spectrum displayed two singlets signals at δ 2.71 and 2.87 ppm, corresponding to six protons from two methyl groups attached to the furyl ring and the carbonyl group. A singlet at δ 7.24 ppm was assigned to the CH proton of the furyl ring. The absence of the singlet at δ 4.22 ppm, which indicates methylene (CH₂) protons, further confirmed the formation of the desired compound (Supplementary Figure S5). In the ^13^C NMR spectrum, signals at δ 12.11 and 27.33 ppm were assigned to the two methyl carbons attached to the furyl ring and the carbonyl group, respectively. Multiple signals in the range of δ 115.71–147.12 ppm corresponded to aromatic carbons from the phenyl and furyl rings. The carbon of the furyl ring bonded to a methyl group appeared at δ 159.78 ppm. The signal at δ 180.00 ppm confirmed the presence of acetyl carbonyl carbon Additionally, the absence of a signal at δ 49.33 ppm, which is attributed to the CH₂ carbon, along with the lack of the carbonyl signal at 176.02 ppm (associated with acyl chloride), further confirmed the structure of the compound (Supplementary Figure S**6**).

The IR spectrum of compound **3a** showed a prominent absorption band at 3050 cm⁻^1^, corresponding to the stretching vibration of aromatic alkene (CH=CH) groups. A significant shift in the carbonyl absorption was observed, appearing at 1690 cm⁻^1^ instead of the typical 1722 cm⁻^1^, indicating the presence of a carbonyl group within an α,β-unsaturated ketone system. This shift is attributed to the electronic effects resulting from the substitution of the methyl group with a conjugated CH=CH–Ar moiety. Additionally, a band at 1527 cm⁻^1^ was assigned to the stretching vibrations of the alkene carbon–carbon double bonds (Supplementary Figure S**7**). In the mass spectrometry analysis, a molecular ion signal at 498 m/z [M +] was observed; this peak was congruent with the molecular formula C_28_H_19_ClN_2_O_3_S and aligned with the expected molar mass of the hypothesized molecular structure (Supplementary Figure S**8**). Moreover, a singlet at δ 2.58 ppm in the proton nuclear magnetic resonance (^1^H NMR) spectrum indicated the presence of three protons of a methyl group. This signal appeared downfield from the original δ 2.71 ppm, reflecting the change in the surrounding chemical environment. Two doublets at δ 6.95 and 7.12 ppm were attributed to the vinylic protons (CH=CH). The absence of the singlet at δ 2.87 ppm, previously corresponding to three methyl protons attached to a carbonyl group, further confirmed the successful transformation of the compound (Supplementary Figure S**9**). Furthermore, the carbon nuclear magnetic resonance (^13^C NMR) spectrum showed a signal at δ 12.30 ppm corresponding to the methyl carbon, along with signals at δ 131.63 and 135.68 ppm, indicating the presence of two vinylic (CH=CH) carbons. Additionally, two downfield signals at δ 176.53 ppm confirmed the presence of an α,β-unsaturated ketone system. The shift from the original δ 180.00 ppm is attributed to the substitution of the methyl group with vinylic carbons, which alters the electronic environment of the carbonyl carbon (Supplementary Figure S**10**).

The infrared spectrum of compound **3b** exhibited distinct peaks, including an absorption band at 3069 cm⁻^1^ corresponding to the stretching vibrations of aromatic alkene (CH=CH) protons. A peak observed at 1688 cm⁻^1^, instead of the typical 1722 cm⁻^1^, indicated the presence of a carbonyl group within an α,β-unsaturated ketone system. This shift is attributed to electronic effects resulting from the substitution of the methyl group with a conjugated CH=CH–Ar moiety. Additionally, a band at 1533 cm⁻^1^ was assigned to the stretching vibrations of the two alkene carbon atoms (Supplementary Figure S**11**). In the proton nuclear magnetic resonance (^1^H NMR) spectrum, a singlet at δ 2.51 ppm indicated the presence of three protons from a methyl group. This signal appeared downfield relative to the original δ 2.71 ppm, reflecting the influence of the altered electronic environment. Two doublets at δ 6.81 and 7.28 ppm were attributed to the vinylic protons (CH=CH). The absence of the singlet at δ 2.87 ppm, previously corresponding to three methyl protons attached to a carbonyl group, further supported the successful structural transformation of the compound (Supplementary Figure S**12**). The carbon nuclear magnetic resonance (^13^C NMR) spectrum showed a signal at δ 12.30 ppm corresponding to the methyl carbon, along with signals at δ 132.64 and 136.10 ppm, indicating the presence of two vinylic (CH=CH) carbon atoms. Additionally, a downfield signal at δ 176.54 ppm confirmed the presence of the carbonyl carbon within an α,β-unsaturated ketone system. The shift from the original δ 180.00 ppm is attributed to the replacement of the methyl group with vinylic carbons, which alters the electronic environment around the carbonyl carbon (Supplementary Figure S**13**).

The infrared spectrum of compound **3c** exhibited distinct absorption bands, including a peak at 3069 cm⁻^1^ corresponding to the stretching vibrations of aromatic alkene (CH=CH) protons, and another at 2998 cm⁻^1^ attributed to the methoxy group. A notable absorption at 1687 cm⁻^1^, downshifted from the typical 1722 cm⁻^1^, indicated the presence of a carbonyl group within an α,β-unsaturated ketone system. This shift is attributed to electronic effects resulting from the substitution of the methyl group with a conjugated CH=CH–Ar moiety. Additionally, a band at 1558 cm⁻^1^ was assigned to the stretching vibrations of the alkene carbon–carbon double bonds (Supplementary Figure S**14**). In the proton nuclear magnetic resonance (^1^H NMR) spectrum, a singlet at δ 2.50 ppm was observed, indicating the presence of three protons from a methyl group. This signal appeared downfield compared to the original δ 2.71 ppm, reflecting changes in the surrounding chemical environment. An additional singlet at δ 3.95 ppm was assigned to three methoxy protons. Two doublets at δ 6.92 and 7.28 ppm were attributed to vinylic protons (CH=CH). The absence of the singlet at δ 2.87 ppm, previously corresponding to three methyl protons attached to a carbonyl group, further confirmed the structural modification of the compound (Supplementary Figure S**15**). The carbon nuclear magnetic resonance (^13^C NMR) spectrum revealed a signal at δ 12.33 ppm, corresponding to the methyl carbon, and another at δ 55.30 ppm, attributed to the methoxy carbon. Additional signals at δ 129.47 and 136.10 ppm confirmed the presence of two vinylic (CH=CH) carbon atoms. A downfield signal at δ 176.54 ppm was consistent with the carbonyl carbon in an α,β-unsaturated ketone system. The observed shift from δ 180.00 ppm supports the electronic influence of vinylic substitution on the carbonyl environment (Supplementary Figure S**16**).

The infrared spectrum of compound **3d** exhibited distinct absorption bands, including a peak at 3071 cm⁻^1^ corresponding to the stretching vibrations of aromatic alkene (CH=CH) protons. A notable absorption at 1686 cm⁻^1^, downshifted from the typical 1722 cm⁻^1^, indicated the presence of a carbonyl group within an α,β-unsaturated ketone system. This shift is attributed to electronic effects resulting from the substitution of a methyl group with a conjugated CH=CH–Ar moiety. Additionally, a band at 1560 cm⁻^1^ was assigned to the stretching vibrations of alkene carbon–carbon double bonds (Supplementary Figure S**17**).The mass spectrum displayed a molecular ion peak at *m/z* 454 [M⁺], consistent with the molecular formula C₂₆H₁₈N₂O₄S, thereby supporting the proposed structure (Supplementary Figure S**18**). In the proton nuclear magnetic resonance (^1^H NMR) spectrum, a singlet at δ 2.50 ppm was observed, indicating the presence of three protons from a methyl group. This signal appeared downfield compared to the original δ 2.71 ppm, reflecting a change in the electronic environment. Two doublets at δ 6.90 and 7.28 ppm were attributed to vinylic protons (CH=CH). The absence of a singlet at δ 2.87 ppm, which previously corresponded to three methyl protons attached to a carbonyl group, further confirmed the successful structural modification (Supplementary Figure S**19**).The carbon nuclear magnetic resonance (^13^C NMR) spectrum revealed a signal at δ 14.10 ppm corresponding to the methyl carbon. Additional signals at δ 130.27 and 132.45 ppm were assigned to two vinylic (CH=CH) carbon atoms. A downfield signal at δ 176.54 ppm was consistent with the carbonyl carbon in an α,β-unsaturated ketone system. The observed shift from δ 180.00 ppm supports the electronic influence of vinylic substitution on the carbonyl environment (Supplementary Figure S**20**).

**Scheme 2:**
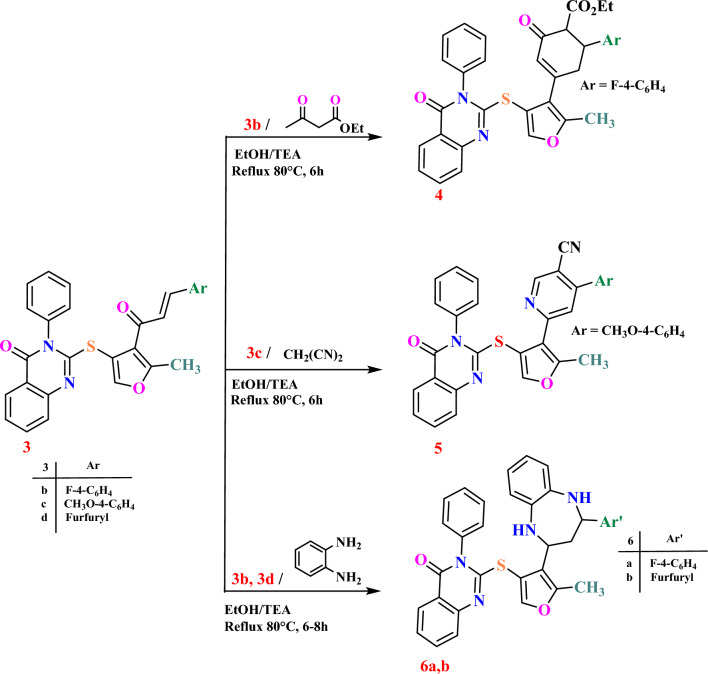
Synthesis of quinazolinone-based derivatives** 4**–**6a**,**b**.

Chalcones **3b**–**d** were capable of forming oxo cyclohexene carboxylate **4**, nicotinonitrile **5**, and benzodiazepine derivatives **6a**,**b** through the reaction of **3b** with ethyl acetoacetate, the response of **3c** with malononitrile, and the reaction of **3b**,**d** with o-phenylene diamine (Scheme 2). The structures of compounds **4**–**6a**,**b** were confirmed based on their analytical and spectral data.

The IR spectrum of compound **4** showed aliphatic C–H stretching bands at 2989, 2945, and 2873 cm⁻^1^. Two strong absorptions at 1728 and 1686 cm⁻^1^ confirmed the presence of ester and cyclohexenone carbonyl groups (Supplementary Figure S21).In the ^1^H NMR spectrum, a triplet at δ 1.22 ppm corresponded to the ester methyl group, while a doublet at δ 2.27 ppm and a doublet at δ 3.31 ppm were assigned to CH₂ and CH protons of the cyclohexenone ring. A singlet at δ 2.88 ppm indicated a methyl group linked to the furyl moiety. Signals at δ 3.79 ppm and 4.24 ppm represented CH and CH₂ protons adjacent to the fluoroaryl and ester groups, respectively. A singlet at δ 7.25 ppm corresponded to a cyclohexenone ring proton. The absence of vinylic CH=CH signals at δ–6.90–7.28 ppm confirmed the loss of the alkene system (Supplementary Figure **S22**). The ^13^C NMR spectrum displayed methyl and methylene signals at δ 15.57, 27.27, and 34.27 ppm. Ester CH₂ and adjacent CH carbons appeared at δ 61.27 and 65.57 ppm. Aromatic and furyl carbons were seen at δ 159.64 ppm. Three distinct carbonyl carbons were observed at δ 160.03, 176.11, and 192.94 ppm (Supplementary Figure **S23**).

The IR spectrum of compound **5** exhibited a strong absorption at 2221 cm⁻^1^, confirming the presence of a nitrile group, and a band at 1623 cm⁻^1^ attributed to the imine functionality within the pyridine ring. The absence of absorptions at 1687 cm⁻^1^ and 1580 cm⁻^1^, corresponding to the α,β-unsaturated carbonyl and vinylic C=C stretches, respectively, supported the structural transformation (Supplementary Figure S**24**). The mass spectrum showed a molecular ion peak at m/z 542 [M⁺], consistent with the molecular formula C₃₂H₂₂N₄O₃S and confirming the proposed structure (Supplementary Figure S**25**). In the ^1^H NMR spectrum, two singlets at δ 9.00 and 9.28 ppm were assigned to the CH protons of the nicotinonitrile ring. The disappearance of the characteristic vinylic doublets at δ 6.92 and 7.28 ppm further supported the loss of the alkene moiety (Supplementary Figure S**26**). The ^13^C NMR spectrum displayed signals at δ 104.90 ppm (pyridine carbon bonded to CN), δ 114.79 ppm (nitrile carbon), and δ 150.67 ppm (CH carbon adjacent to pyridine nitrogen). A signal at δ 157.08 ppm was attributed to the new imine carbon, with δ 157.00 ppm representing an additional pyridine carbon. The absence of the carbonyl signal at δ 176.54 ppm (previously linked to the furyl ring) confirmed the structural modification (Supplementary Figure S**27**).

The IR spectrum of compound** 6a** exhibited characteristic bands at 3120 and 3155 cm⁻^1^ corresponding to two NH stretching vibrations, and at 2974, 2933, and 2870 cm⁻^1^ due to aliphatic C–H stretching of the diazepine ring. Absence of 1688 cm⁻^1^ band of α,β-unsaturated ketone ensured our new targted compound (Supplementary Figure S**28**). The ^1^H NMR spectrum displayed a doublet of doublets at δ 2.90 ppm attributed to CH₂ protons of the diazepine ring, and a triplet at δ 3.59 ppm corresponding to two CH protons within the ring. A broad exchangeable singlet at δ 6.98 ppm indicated the presence of two NH protons. The absence of doublets at δ 6.81 and 7.28 ppm, previously associated with vinylic protons, confirmed the structural transformation (Supplementary Figure S**29**). The ^13^C NMR spectrum showed a signal at δ 39.35 ppm for the CH₂ carbon of the diazepine ring, and a signal at δ 54.33 ppm assigned to the two CH carbons. A downfield signal at δ 164.44 ppm was attributed to the imine carbon of the diazepine ring, while a signal at δ 168.54 ppm corresponded to the aromatic carbon bearing a fluorine substituent. The disappearance of the carbonyl signal at δ 176.54 ppm (linked to the furyl moiety in the precursor) further supported the successful formation of the target compound (Supplementary Figure S**30**).

The IR spectrum of compound **6b** exhibited characteristic absorption bands at 3227 and 3170 cm⁻^1^, confirming the presence of two N–H groups, and bands at 2949 and 2885 cm⁻^1^ due to aliphatic C–H stretching of dizepine ring. The absence of the band at 1686 cm⁻^1^ (α,β-unsaturated ketone) supported the successful formation of the target structure (Supplementary Figure S**31**). The mass spectrum showed a molecular ion peak at m/z 546 [M⁺], consistent with the molecular formula C₃₂H₂₆N₄O₃S, in agreement with the calculated molecular weight (Supplementary Figure S**32**). The ^1^H NMR spectrum revealed a doublet of doublets at δ 2.90 ppm assigned to CH₂ protons of the diazepine ring, and two triplets at δ 3.05 and 3.09 ppm corresponding to CH protons of the same ring. A singlet at δ 3.41 ppm, exchangeable with D₂O, confirmed the presence of two NH protons. The absence of doublet signals at δ 6.90 and 7.28 ppm, previously assigned to vinylic protons, further verified the structural transformation (Supplementary Figure S**33**).

**Scheme 3:**
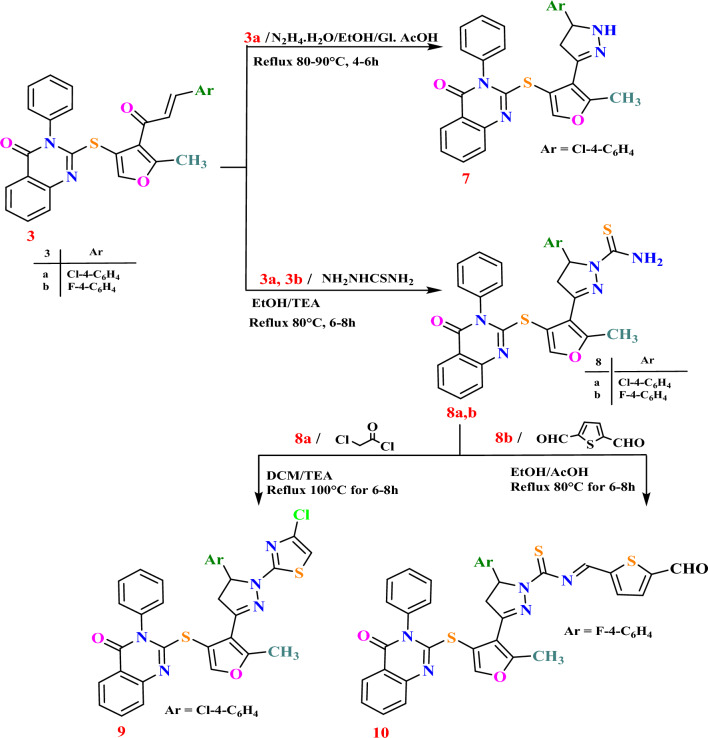
Synthesis of quinazolinone-based derivatives** 7**–**10.**

Chalcone **3a** demonstrated the ability to form pyrazoline **7** and Chalcones **3a,b** formed pyrazole carbothioamide derivatives **8a**,**b** through their reactions with hydrazine hydrate and thiosemicarbazide, respectively. Subsequently, compounds **8a**,**b** underwent further transformations, yielding chlorothiazole derivative **9** via reaction of **8a** with chloroacetyl chloride and formyl thiophen methylene derivative **10** through reaction of **8b** with thiophene-2,5-dicarbaldehyde, as illustrated in Scheme 3.

The molecular structure of compound **7** was confirmed through its spectral data. The IR spectrum displayed a broad absorption at ν 3416 cm⁻^1^, attributed to the N–H group, and a band at ν 1614 cm⁻^1^ corresponding to the imine group within the pyrazole ring (Supplementary Figure S**34**). In the ^1^H NMR spectrum, a doublet at δ 3.89 ppm was assigned to the CH₂ protons of the pyrazole ring, a triplet at δ 4.40 ppm corresponded to the CH proton of the ring, and a broad singlet at δ 9.90 ppm was attributed to the exchangeable NH proton (Supplementary Figure S**35**). The ^13^C NMR spectrum showed signals at δ 46.69 ppm (CH₂ of pyrazole), δ 55.65 ppm (CH of pyrazole), and δ 101.98 ppm for the furyl carbon linked to the pyrazole. A downfield signal at δ 160.00 ppm was assigned to the imine carbon of the newly formed pyrazole system (Supplementary Figure S**36**).

The IR spectrum of compound **8a** showed characteristic peaks at ν 3492 and 3398 cm⁻^1^ corresponding to the amino group, a band at ν 1611 cm⁻^1^ indicating the presence of a pyrazole imine group, and an absorption at 1302 cm⁻^1^ attributed to the thioamide functionality (Supplementary Figure S**37**). The mass spectrum revealed a molecular ion peak at m/z 571.09 [M⁺], consistent with the molecular formula C₂₉H₂₂ClN₅O₂S₂, supporting the proposed structure (Supplementary Figure S**38**). In the ^1^H NMR spectrum, a doublet at δ 3.52 ppm was assigned to the CH₂ protons of the pyrazole ring, a triplet at δ 3.80 ppm corresponded to the CH proton, and a broad exchangeable singlet at δ 9.14 ppm confirmed the presence of NH₂ protons (Supplementary Figure S**39**). The ^13^C NMR spectrum displayed signals at δ 46.10 ppm (CH₂) and 72.25 ppm (CH) of the pyrazole ring. Additional signals at δ 150.67 ppm and δ 176.53 ppm were attributed to the imine and thioamide carbons, respectively (Supplementary Figure S**40**).

The IR spectrum of compound **8b** exhibited sharp peaks at ν 3488 and 3460 cm⁻^1^ corresponding to the amino group, a band at ν 1615 cm⁻^1^ for the pyrazole imine group, and an absorption at ν 1264 cm⁻^1^ attributed to the thioamide functionality (Supplementary Figure S**41**). The ^1^H NMR spectrum showed a doublet at δ 3.59 ppm assigned to the CH₂ protons of the pyrazole ring, a triplet at δ 4.22 ppm for the CH proton, and an exchangeable singlet at δ 10.59 ppm confirming the presence of two NH₂ protons (Supplementary Figure S**42**). In the ^13^C NMR spectrum, signals appeared at δ 46.04 ppm (CH₂) and δ 75.05 ppm (CH) of the pyrazole ring, δ 164.34 ppm (imine carbon), and δ 179.59 ppm (thioamide carbon). An additional signal at δ 168.44 ppm indicated an aromatic carbon bonded to a fluorine atom (Supplementary Figure S**43**).

The IR spectrum of compound **9** showed an absorption band at ν 1642 cm⁻^1^ corresponding to the imine group of the thiazole ring, while the absence of peaks at 3492 and 3398 cm⁻^1^ confirmed the loss of the amino group, supporting the formation of the target compound (Supplementary Figure S**44**). The mass spectrum displayed a molecular ion peak at *m/z* 629 [M⁺], consistent with the molecular formula C₃₁H₂₁Cl₂N₅O₂S₂ and confirming the proposed structure (Supplementary Figure S**45**). The ^13^C NMR spectrum showed a downfield signal at δ 176.54 ppm assigned to the imine carbon of the 4-chlorothiazole ring. The absence of the C=S signal at δ 176.53 ppm further supported the structural transformation (Supplementary Figure S4**6**).

The IR spectrum of compound **10** displayed characteristic bands at ν 2737 and 2677 cm⁻^1^ for aldehydic C–H stretching and a strong band at ν 1710 cm⁻^1^ confirming the presence of an aldehyde carbonyl group. The band at ν 1602 cm⁻^1^ corresponded to newly formed imine group, while the absence of amino group peaks at 3488 and 3460 cm⁻^1^ confirmed successful transformation (Supplementary Figure S**47**). The ^1^H NMR spectrum showed a singlet at δ 7.97 ppm for the vinylic proton adjacent to the thioamide group and a singlet at δ 9.51 ppm for the aldehydic proton. The absence of the exchangeable amino signal at δ 10.59 ppm further supported the structural modification (Supplementary Figure S**48**). The ^13^C NMR spectrum displayed a signal at δ 157.50 ppm for the thiophene carbon linked to an imine group, δ 171.00 ppm for the imine carbon, δ 185.59 ppm for the aldehydic carbonyl carbon, and δ 190.00 ppm for the thioamide carbon, shifted downfield from δ 179.59 ppm due to electronic effects of the new substituents (Supplementary Figure S**49**).

### Biological evaluation

#### In vitro cytotoxic activity

The cytotoxic potency of quinazolinones **1**–**10** was established in vitro by the MTT assay^75–77^ on human liver HepG-2, breast MCF-7, colorectal HCT-116 cancer cell lines, and normal diploid cell line WI-38 at various concentrations of (100–1.56 μM) compared to erlotinib (Table. S1) to ascertain their IC_50_ as shown in Table 1. In comparison to erlotinib (IC_50_ = 4.50 ± 0.2, 5.23 ± 0.3, and 4.17 ± 0.2 μM against HepG-2, HCT-116, and MCF-7 cells, respectively), quinazolinone derivatives **2**–**5**, **6a**, **7**, **8b**, and **9** showed moderate to weak cytotoxicity against all screened cell lines with an IC_50_ range of 16.04–93.38 μM. In contrast, derivatives **1**, **6b**, **8a**, and **10** demonstrated excellent activity (IC_50_ range = 5.80–13.23, 7.97–19.63, and 3.91–10.58 μM against HepG-2, HCT-116, and MCF-7 cells, respectively). Furthermore, the MTT assay was employed to examine the safety profiles of the cytotoxic activity against the normal human diploid cell line WI-38 (Table 1). The IC_50_ values of all drugs were higher than those of erlotinib (range = 17.75 ± 1.4 to ˃100 µM compared to 6.72 ± 0.5 µM for erlotinib). They could therefore be considered safe chemotherapeutic agents.

**Table 1:** The antitumor activities of quinazolinones **1**–**10** upon human tumor HepG-2, HCT-116, MCF-7, and normal WI-38 cell lines at different concentrations to express as IC_50_ values using MTT assay.

Compd. no.	IC50 (mean ± SD) (µM)			
	HepG-2	HCT-116	MCF-7	WI-38
**1**	13.23 ± 1.1	19.63 ± 1.4	10.58 ± 0.9	75.41 ± 3.8
**2**	47.83 ± 2.8	78.72 ± 3.7	54.87 ± 3.2	36.90 ± 2.4
**3a**	21.77 ± 1.6	34.11 ± 2.1	37.90 ± 2.2	68.72 ± 3.6
**3b**	56.02 ± 3.2	83.32 ± 4.1	71.12 ± 3.8	28.78 ± 1.9
**3c**	69.58 ± 3. 6	˃100	23.44 ± 1.6	53.17 ± 2.9
**3d**	18.85 ± 1.4	25.91 ± 1.7	16.04 ± 1.3	˃100
**4**	64.88 ± 3.5	87.78 ± 4.3	62.13 ± 3.5	44.87 ± 2.7
**5**	38.52 ± 2.3	66.31 ± 3.5	49.96 ± 2.8	17.75 ± 1.4
**6a**	35.77 ± 2.1	52.53 ± 3.1	33.64 ± 2.0	85.10 ± 4.2
**6b**	5.80 ± 0.4	7.97 ± 0.5	3.91 ± 0.2	41.26 ± 2.5
**7**	27.91 ± 1.9	48.89 ± 2.6	45.81 ± 2.5	˃100
**8a**	9.68 ± 0.8	57.13 ± 3.3	8.29 ± 0.6	63.20 ± 3.3
**8b**	81.53 ± 4.2	93.38 ± 4.7	76.09 ± 3.9	26.91 ± 1.8
**9**	43.95 ± 2.5	74.71 ± 3.9	58.03 ± 3.4	39.81 ± 2.4
**10**	8.71 ± 0.7	11.60 ± 0.9	6.49 ± 0.4	35.17 ± 2.2
**Erlotinib**	4.50 ± 0.2	5.23 ± 0.3	4.17 ± 0.2	6.72 ± 0.5

IC_50_: Compound concentration required to inhibit growth by 50%, SD: Standard deviation; each value is the mean of three values, (–) not detected.

### Structure–activity relationship study

The above results in Table 1 indicated that, parent compound (4-oxo-3-phenylquinazolin-2-yl) 2-chloroethanethioate (**1)** recorded moderate activity (IC_50_ = 13.23 ± 1.1, 19.63 ± 1.4 and 10.58 ± 0.9 μM against HepG-2, HCT-116 and MCF-7 cell lines, respectively). Cyclization of chloroethanethionate fragment to give 5-methylfuran-3-yl moiety attached to quinazoline scaffold by thio-linker in **2**, revealed reduction in cytotoxicity (IC_50_ = 47.83 ± 2.8, 78.72 ± 3.7 and 54.87 ± 3.2 μM, respectively). Replacement of the acetyl group in **2** with other chalcones in **3a**–**d**, ethyl 4′-fluoro-3-oxo-1,2,3,6-tetrahydro-[1,1′-biphenyl]-2-carboxylate **4** or 4-(4-methoxyphenyl)nicotinonitrile **5**, exhibited poor cytotoxic activity (IC_50_ range = 18.85–69.58, 25.91– > 100 and 16.04–71.12 μM against HepG-2, HCT-116 and MCF-7 cells, respectively). On the other hand, replacement with benzo[*b*][1,4]diazepine bearing 4-fluorophenyl at p-2 remained the weak activity in **6a** (IC_50_ = 35.77 ± 2.1, 52.53 ± 3.1 and 33.64 ± 2.0 μM, respectively), while substitution with furfuryl moiety illustrated better cytotoxicity in **6b** (IC_50_ = 5.80 ± 0.4, 7.97 ± 0.5 and 3.91 ± 0.2 μM, respectively). In addition, replacement of acetyl group in **2** with pyrazoline moiety with free N-1 in **7** or bound to thiazole in **9**, yielded weak activity.

**Fig. 3:**
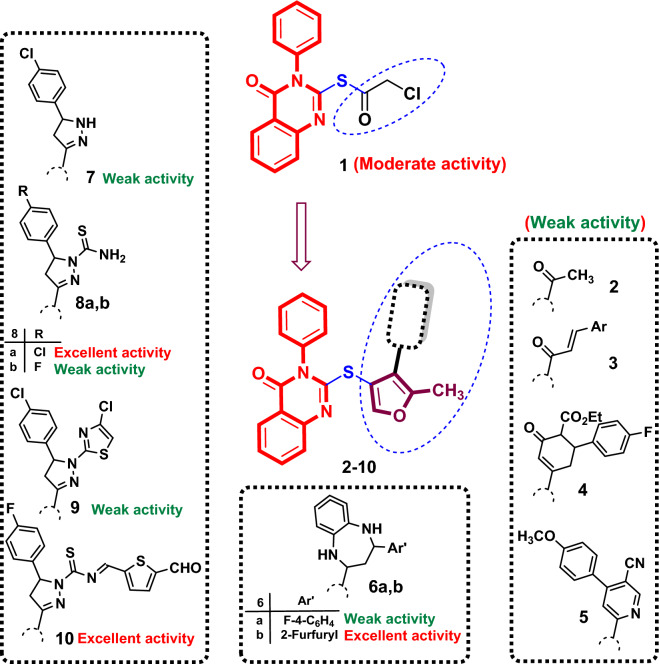
SAR study of quinazolinone-based derivatives** 1**–**10** as cytotoxic agents against human cancerous HepG-2, HCT-116 and MCF-7 cell lines.

The pyrazoline nucleus with carbothioamide at *N*-1 in **8** showed contrasting activities: Excellent with *p*-chlorophenyl in **8a** (IC_50_ = 9.68 ± 0.8 and 8.29 ± 0.6 μM against HepG-2 and MCF-7 cell lines, respectively) and weak with *p*-fluorosubstitution in **8b** (IC_50_ = 81.53 ± 4.2, 93.38 ± 4.7 and 76.09 ± 3.9 μM, respectively). Finally, substitution of *N*-1 of pyrazoline with *N*-((5-formylthiophen-2-yl)methylene)carbothioamide **10**, demonstrated elevated and excellent cytotoxicity (IC_50_ = 8.71 ± 0.7, 11.60 ± 0.9 and 6.49 ± 0.4 μM, respectively) (Fig. 3).

In summary, quinazolinone-based hybrids featuring a 5-methylfuran-3-yl moiety linked via a thio-linker and incorporating 2-furfurylbenzo[*b*][1,4]diazepine, 5-(4-chlorophenyl)-4,5-dihydro-1*H*-pyrazole-1-carbothioamide, or 5-(4-fluorophenyl)-*N*-[(5-formylthiophen-2-yl)methylene]-4,5-dihydro-1*H*-pyrazole-1-carbothioamide demonstrated remarkable potency against the screened cancer cell lines HepG-2, HCT-116, and MCF-7.

### In vitro enzyme inhibitory assessment against EGFR and VEGFR-2

Quinazolinone-based targets **1, 6b**,** 8a,** and **10** were chosen for further evaluation of their in vitro inhibitory activity against EGFR and VEGFR-2 because of their exceptional cytotoxic outcomes, with the goal of clarifying their mode of action. Erlotinib and sorafenib were used as standards^71,78,79^ for their respective IC_50_ values, which are shown in Table 2. By inspection of data in Table 2, it was noted that 2-furfurylbenzo[*b*][1,4]diazepine **6b** and *N*-((5-formylthiophen-2-yl)methylene)pyrazoline-1-carbothioamide **10** established promising inhibitory potency against EGFR with superior results of **6b** over **10** compared with erlotinib (IC_50_ = 0.19 ± 0.03, 0.51 ± 0.04 and 0.23 ± 0.02 μM, respectively). On the other hand, the rest screened derivatives **1** and **8a** gave weak inhibitory activity with IC_50_ values 25.46 ± 1.20 and 8.53 ± 0.25 μM, respectively. Concerning VEGFR-2, all screened quinazolinones **1, 6b**,** 8a**, and **10** revealed weak inhibitory efficacy (IC_50 range_ = 31.65–70.18 μM) when compared to sorafenib (IC_50_ = 0.83 ± 0.10 μM).

**Table 2:** In vitro Inhibitory assessment of the promising quinazolinone-based derivatives **1, 6b**,** 8a,** and **10** against EGFR and VEGFR-2 compared with erlotinib and sorafenib, respectively.

Compd. no.	IC_50_ (mean ± SD) (µM)	
	EGFR	VEGFR-2
1	25.46 ± 1.20	70.18 ± 2.55
6b	0.19 ± 0.03	31.65 ± 1.48
8a	8.53 ± 0.25	46.15 ± 1.20
10	0.51 ± 0.04	39.42 ± 1.35
Erlotinib	0.23 ± 0.02	–
Sorafenib	–	0.83 ± 0.10

According to prior findings, screened derivatives, particularly **6b** and **10**, appear to be substantially more selective for EGFR than VEGFR-2.

IC_50_: Compound concentration necessary to inhibit the enzyme activity by 50%, SD: Standard deviation; each value is the mean of three values, (–) not detected.

### Cell cycle arrest and apoptosis of compound 6b

**Fig. 4:**
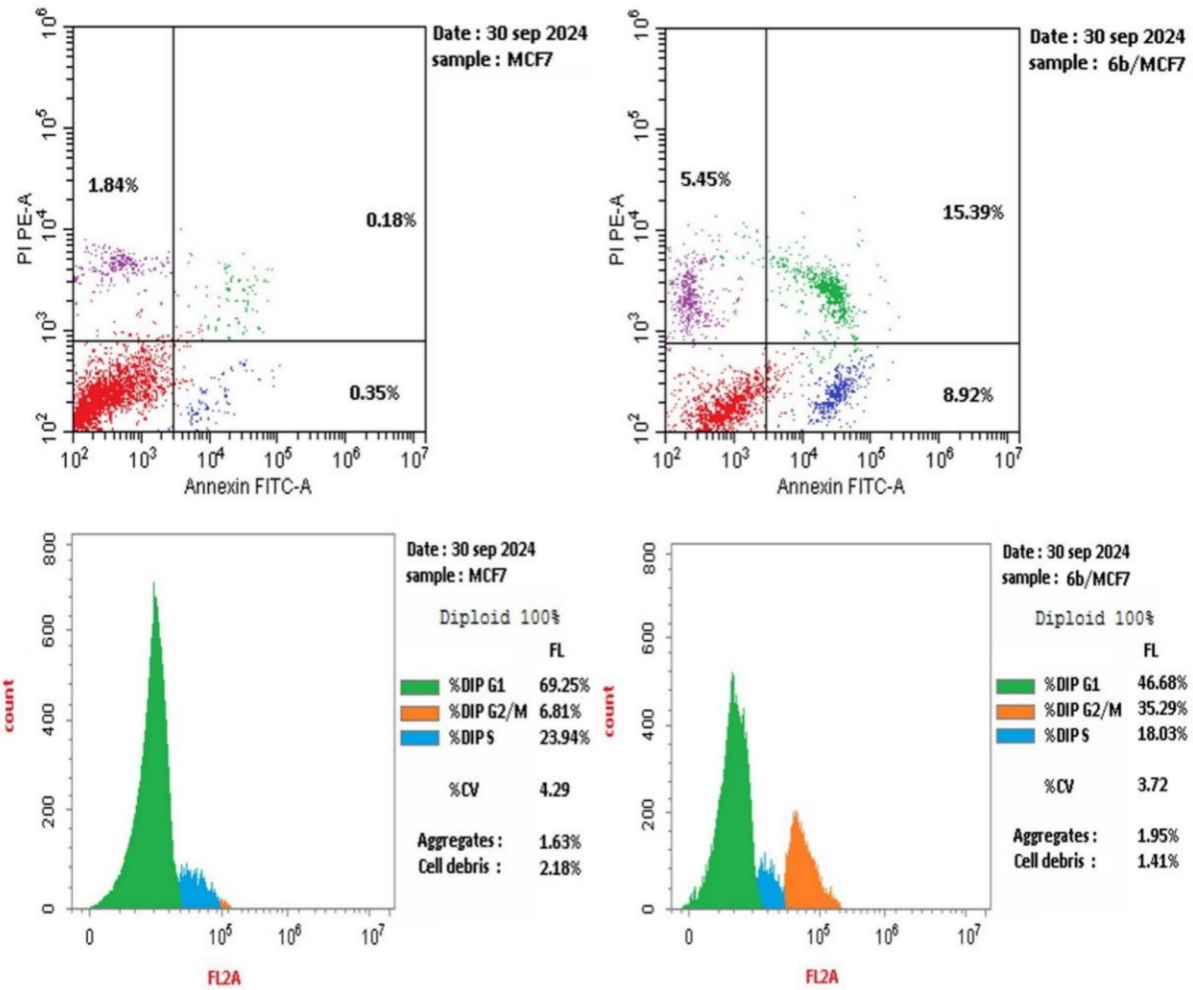
Examination of cell cycle and influence of quinazolinone-furan-benzo[*b*][1,4]diazepine **6b** on the percentage of V-FITC-positive annexin staining in MCF-7 cells regarding control.

**Fig. 5:**
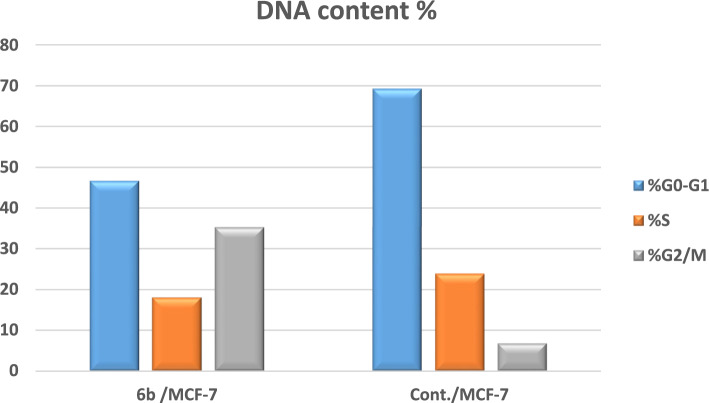
Examination of cell cycle with quinazolinone-furan-benzo[b][1,4]diazepine **6b.**

**Fig. 6:**
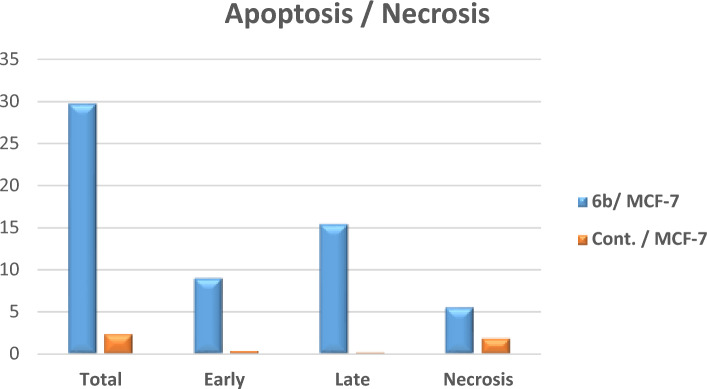
Influence of quinazolinone-furan-benzo[*b*][1,4]diazepine **6b** on apoptotic activity.

To investigate whether quinazolinone-furan-benzo[*b*][1,4]diazepine **6b**, the most potent derivative, induces cell death via the apoptotic pathway, MCF-7 cells were treated for 24 h at a concentration of 3.91 μM. The treated cells were subsequently analyzed using flow cytometry and annexin-V labeling (Fig. 4, 5, 6). As illustrated in Fig. 5 and Table S2, quinazolinone **6b** significantly increased cell accumulation in the G2/M phase to 35.29%, compared to only 6.81% in untreated MCF-7 cells. These results strongly suggest that compound **6b** effectively arrests MCF-7 cells at the G2/M phase of the cell cycle. Furthermore, as presented in Fig. 6 and Table S3, treatment with compound **6b** led to a notable increase in late apoptosis from 0.18% (DMSO control) to 15.39%, alongside a considerable rise in early apoptosis from 0.35% to 8.92%. Additionally, the necrosis rate increased to 5.45% compared to 1.84% in the DMSO control. These findings suggest that quinazolinone **6b** induces apoptosis in MCF-7 cells, as evidenced by the significant increase in apoptotic cell populations.

### Impact of quinazolinone-furan-benzo[b][1,4]diazepine 6b upon Bax, Bcl-2 and p53 levels in MCF-7 cells

The extrinsic pathway, which is controlled by the death receptor, and the intrinsic pathway, which is handled by the mitochondria, are the two primary mechanisms that govern the cell during apoptosis. The two proteins Bcl-2 and Bax can alter this planned process due to their respective roles as pro- and anti-apoptotic (inducer), and the balance between them adjusts cell death^80^. A further vital component that either causes cell death or prevents cell proliferation is the tumor suppressor gene, p53. Cancers that continue to exhibit p53 suppression and genomic stability may proliferate more rapidly and develop resistance to a variety of anticancer therapies^51^. Compared to untreated control cells (38.45 Pg/mL), MCF-7 cells exposed to compound **6b**’s IC_50_ of 3.91 μM for 24 h displayed a 6.3-fold rise in Bax levels (241.60 Pg/mL). Conversely, Bcl-2 protein was downregulated 2.6 times, from 7.31 to 2.82 ng/mL. Furthermore, compound **6b** increased the amount of p53 protein in MCF-7 treated cells by 7.2 times, while control cells only had 119.65 Pg/mL (Table 3).

**Table 3:** Impact of quinazolinone-furan-benzo[*b*][1,4]diazepine **6b** upon Bax, Bcl-2 and p53 levels.

Compd. No.	Bax (Pg/mL)	Bcl-2 (ng/mL)	Bax/Bcl-2	P53 (Pg/mL)
6b / MCF-7	241.6 ± 1.33	2.82 ± 0.11	85.67	860.55 ± 2.47
Control / MCF-7	38.45 ± 1.50	7.31 ± 0.30	5.26	119.65 ± 2.55

### In silico studies

#### ADMET elucidation

By employing the free online resource SwissADME to examine the targeted medications’ absorption, distribution, metabolism, excretion, and toxicity (ADMET), crucial information about the best treatment option can be provided^81–84^. The recommendations provided by Veber and Lipinski may be used to ascertain which drug performs the best when taken orally. Therefore, promising quinazolinones **6b**,** 8a,** and **10** were studied for their expected ADMET. As shown in Table 4, With one exception to the Lipinski rule (MW > 500), it was demonstrated that 2-furfurylbenzo[*b*][1,4]diazepine **6b** complied with the prior regulations. On the other hand, the rest of the screened derivatives, **8a** and **10,** didn’t obey both rules, with one violation of the Veber rule (TPSA > 140) and two violations of Lipinski (MW > 500, MLOGP > 4.15).

**Table 4:** Promising quinazolinones **6b**,** 8a,** and **10** and their computed physicochemical properties.

Compd.	MWa	TPSA (Å^2^)^b^	nRB^c^	nHBA^d^	nHBD^e^	MLogP^f^	Violations^g^
Rule	≤ 500	≤ 140	≤ 10	≤ 10	≤ 5	≤ 4.15	–
**6b**	544.62	110.53	5	4	2	3.41	1 (Lipinski)
							MW > 500;
							0 (Veber)
**8a**	572.10	147.04	6	4	1	4.35	2 (Lipinski)
							MW > 500, MLOGP > 4.1;
							1 (Veber)
							TPSA > 140
**10**	677.79	178.69	9	7	0	4.69	2 (Lipinski)
							MW > 500, MLOGP > 4.1;
							1 (Veber)
							TPSA > 140

^a^Molecular Weight; ^b^Topological Polar Surface Area; ^c^Number of Rotatable Bond; ^d^Number of Hydrogen Bond Acceptor; ^e^Number of Hydrogen Bond Donor; ^f^Calculated Lipophilicity (MLog Po/w); ^g^Violations from Lipinski and Veber Rules.

**Fig. 7:**
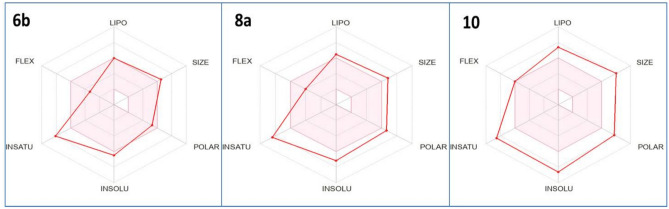
The radar map of effective quinazolinones **6b**, **8a,** and **10**. The predicted values for the examined objectives are indicated by the red lines, while the pink area indicates the ideal values for each oral bioavailability component.

The assessed quinazolinone-based candidate **6b** was in the optimal range (pink zone) of the key features (lipophilicity, size, polarity, and flexibility), according to the bioavailability radar chart (Fig. 7), with the exception of solubility and saturation, giving powerful proof of its oral bioavailability. While derivatives **8a** and **10** deviated from all criteria except flexibility, indicating difficulty in oral administration.

Table 5 shows that potential quinazolinone target **6(b)** has a higher chance of gastrointestinal absorption, while others do not, with no blood–brain barrier penetration for all. Therefore, **6(b)** may be utilized to treat peripheral disorders without resulting in central problems. P-glycoprotein, commonly known as a drug efflux transporter, plays a role in removing drugs from cells and may influence drug tolerance. Since none of the screening quinazolinones **6b**, **8a**, and **10** are P-gp substrates, there is minimal efflux out of the cell with the highest activity. In fact, research has shown that blocking more CYP enzymes raises the possibility that a drug may interact with other chemical compounds in a situation known as drug-drug interaction (DDI)^83^. It was therefore expected that these medications would not significantly affect the majority of CYPs.

**Table 5:** Anticipated pharmacokinetic properties of quinazolinones **6b**,** 8a,** and **10**.

Pharmacokinetic properties	Compd. No.		
	**6b**	**8a**	**10**
GIT absorption	High	Low	Low
BBB permeability	NO	NO	NO
P-gp substrate	NO	NO	NO
CYP1A2 inhibitor	YES	NO	YES
CYP2C19 inhibitor	NO	NO	NO
CYP2C9 inhibitor	YES	YES	NO
CYP2D6 inhibitor	NO	NO	NO
CYP3A4 inhibitor	YES	YES	YES
Bioavailailityscore	0.55	0.17	0.17
PAINS alert	0	0	0

### Molecular docking simulation

A docking simulation was conducted to demonstrate how the in vitro inhibitory assessment results of quinazolinones **6b**, **8a**, and **10** varied upon EGFR kinase. First, the docking protocols were confirmed by re-docking the native ligand, erlotinib, in the EGFR active site (PDB code: 1M17)^84,85^ . As a result, the energy score value between the native ligand and its docked position was − 10.93 kcal/mol, with a relatively tiny RMSD value (0.81 Å).

The original ligand, erlotinib, was fitted into the EGFR active site by hydrophobic connections, as shown in Fig. S1, and it was enhanced through the quinazoline moiety’s H-bonding with the essential amino acid **Met769** (distance: 1.90 Å).

**Fig. 8:**
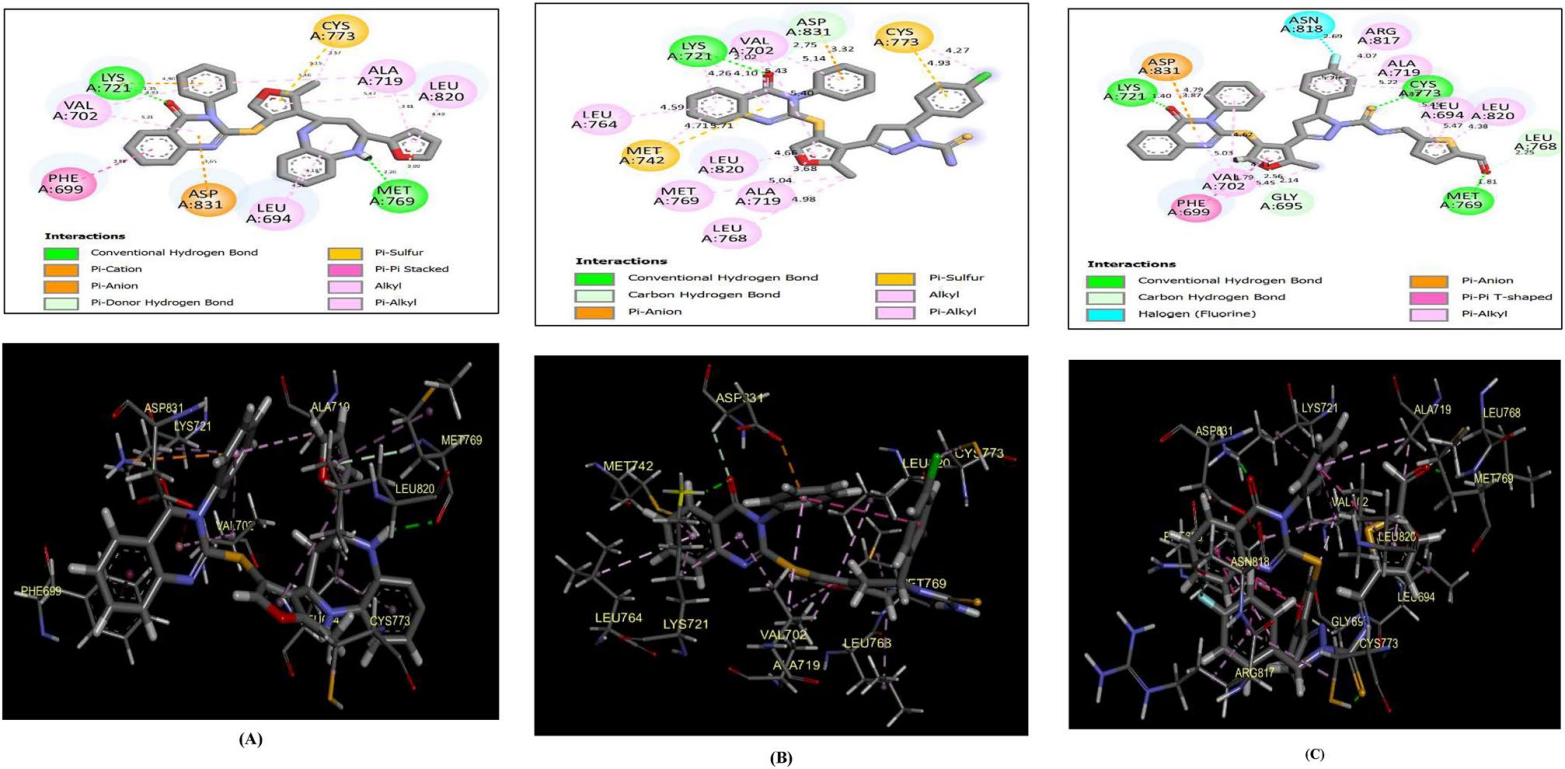
(**A**, **B**, and **C)** views illustrated (2D and 3D) binding features of the quinazolinones **6b**, **8a,** and **10** within the active site of EGFR (PDB code: 1M17), respectively.

Then, the screened quinazolinones **6b**, **8a**, and **10** were docked, giving energy score values of − 11.26, − 7.53, and − 10.85 kcal/mol, respectively. Figure 8 demonstrates that the quinazolinone scaffold of all derivatives binds to EGFR via H-bonding between the carbonyl oxygen and **Lys 721**. On the other hand, the key amino acid **Met769** afforded H-bonding with benzo[*b*][1,4]diazepine-*N*1 in **6b** and formyl oxygen in **10** (distance: 2.20 and 1.81 Å, respectively). Furthermore, carbothioamide sulfur in **10** formed H-bond acceptor with the backbone of **Cys773** (distance: 1.87 Å). Additional hydrophobic contacts appeared with **Phe699**, **Val702**, **Leu820**, and **Asp831** in **6b**, **8,** and **10**.

### Quantum chemical calculation

The quantum chemical features of the studied synthetic compounds are presented in Table 6, with particular attention to the energy gap (ΔE), highest occupied molecular orbital (HOMO), and lowest unoccupied molecular orbital (LUMO) energies in table S4 (Supplemetary file). These parameters are crucial in determining the stability, reactivity, and potential biological activity of the compounds.

**Table 6:** Computed Quantum Chemical Calculation of the Assembled compounds (**1**–**10**).

Compd. No.	LUMO	HOMO	ΔE	A	I	X	η	S or σ	ω	ΔN max
1	− 0.1689	− 0.22645	0.05752	0.1689	0.22645	0.19769	0.02876	34.7705	1.35887	3.43689
2	− 0.1693	− 0.21764	0.04834	0.1693	0.21764	0.19347	0.02417	41.3736	1.54864	4.002275
3a	− 0.1898	− 0.22757	0.03773	0.1898	0.22757	0.208705	0.01886	53.0082	2.30892	5.530082
3b	− 0.1902	− 0.22817	0.03795	0.1902	0.22817	0.209195	0.01897	52.7009	2.30632	5.512384
3c	− 0.1919	− 0.2291	0.03711	0.1919	0.2291	0.210545	0.01855	53.8938	2.38907	5.673538
3d	− 0.2190	− 0.2211	0.00206	0.2190	0.2211	0.220065	0.00103	9.66183	46.7909	106.3115
4	−0.1760	− 0.23643	0.0604	0.1760	0.23643	0.220065	0.00103	33.112	1.40830	3.414403
5	− 0.1681	− 0.19327	0.0251	0.1681	0.19327	0.18072	0.01255	79.6812	2.60236	7.2
6a	− 0.2240	− 0.22563	0.00159	0.2240	0.22563	0.22483	0.00079	1250.00	63.1856	140.5187
6b	− 0.2490	− 0.25165	0.00258	0.2490	0.25165	0.250355	0.00129	772.20	48.3997	96.6621
7	− 0.1680	− 0.23959	0.07151	0.1680	0.23959	0.203835	0.0357	27.9681	1.16203	2.850440
8a	− 0.1680	− 0.22492	0.05687	0.1680	0.22492	0.196485	0.02843	35.1679	1.35770	3.454985
8b	− 0.1680	− 0.22674	0.05869	0.1680	0.22674	0.197395	0.02934	34.0773	1.3278	3.363349
9	− 0.1679	−0.23905	0.07106	0.1679	0.23905	0.20352	0.03553	28.1452	1.16578	2.86405
10	− 0.1812	− 0.23137	0.05009	0.1812	0.23137	0.206325	0.02504	39.9281	1.69974	4.11908

### HOMO–LUMO energy gap (ΔE) and reactivity

The HOMO–LUMO energy gap (ΔE) is a quantum descriptor that is key to the determination of the electronic properties and reactivity of a molecule. The value of ΔE is the energy difference between the HOMO and LUMO levels, and it represents the capacity of the molecule to be electronically excited and to interact with biological systems. Lower ΔE signifies higher reactivity and potential biological interactions because electron transfer is facilitated.

The electronic structure of these compounds may be visualized more effectively employing electrostatic potential (ESP) maps. ESP maps highlight electron density distribution in which low density areas (positive ESP) favor nucleophilic attack and regions of high electron density (negative ESP) favor electrophilic attack. These aspects play a crucial role in the understanding of the binding capacity of such compounds to biological targets such as DNA and enzymes related to cancer.

According to the quantum chemical calculation, Compound **6a** exhibits the lowest ΔE value of 0.00159 eV, indicating very high chemical reactivity. Conversely, Compound **4** exhibits the highest ΔE of 0.0604 eV, indicating relatively lower reactivity. Notably, compounds with lower ΔE values are expected to bind to biological targets such as DNA or cancer-related proteins and suppress cancer cell proliferation.

### Insights into anticancer potential

Lower ΔE-compounds are more reactive and thus more prone to interact with cancer interest targets. Below mentioned are some key conclusions, based on a combined study of HOMO, LUMO, ΔE, and ESP:

- *Strongly reactive compounds* Compounds **6a**, **6b**, and **3d** with the lowest ΔE values should strongly interact with key components of critical cancer cells, including DNA and essential enzymes of cell growth. Such interactions have the potential to cause anticancer activity by promoting apoptosis or disrupting vital metabolic pathways.
- *Moderate LUMO values* Compounds such as **8a** and **10**, having moderate LUMO values, can be used as enzyme inhibitors by binding with electron-rich cancer-associated proteins and affecting critical biochemical processes.
- *High HOMO values* A compound with a high HOMO, such as Compound **5**, has a higher tendency to bind with oxidative stress centers in cancer cells, causing oxidative damage and cell death.
- *Selective toxicity potential* If they are made available, ESP maps can also be employed to anticipate the selective binding of such molecules to cancer cells over normal cells, which could reduce cytotoxicity effects and increase therapeutic selectivity.

**Fig. 9:**
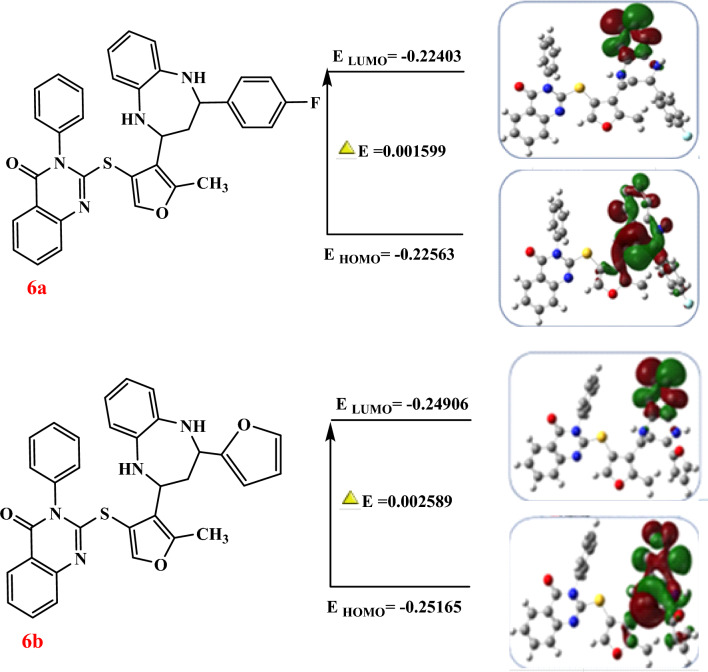
DFT-optimized structures and HOMO/LUMO (highest occupied molecular orbital/lowest unoccupied molecular orbital) diagrams of compounds **6a** and **6b**.

Based on HOMO–LUMO characteristics and ΔE values, compounds **6a** and **6b** are the best anticancer prospects due to their high reactivity and good electronic character (Fig. 9). Further investigations, including molecular docking and in vitro anticancer screening, was carried out to verify these forecasts and elicit their specificity and potency against cancer cells. These results provide a breakthrough opportunity for designing efficient anticancer agents employing quantum chemical descriptors.

## Conclusion

This work designed and synthesized a novel series of 2- [(4-substituted-5-methylfuran-3-yl)thio]-3-phenylquinazolin-4(3*H*)-one derivatives **2**–**10** as possible anti-cancer entities. Three malignant human liver HepG-2, breast MCF-7, colorectal HCT-116, and one normal diploid cell line, WI-38, were used to test the newly synthesized substance’s anti-proliferative efficacy. When compared to erlotinib (IC_50_ = 4.50 ± 0.2, 5.23 ± 0.3, and 4.17 ± 0.2 μM against HepG-2, HCT-116, and MCF-7, respectively), quinazolinone-based targets **1**, **6b**, **8a**, and **10** demonstrated exceptional activity (IC_50_ range = 5.80–13.23, 7.97–19.63, and 3.91–10.58 μM against HepG-2, HCT-116, and MCF-7 cells, respectively). To elucidate their mechanism of action, these compounds were chosen for additional evaluation of their in vitro inhibitory performance against EGFR and VEGFR-2. Both **6b** and **10** showed promising inhibitory effectiveness against EGFR, with **6b** surpassing. According to flow cytometric analysis, the most potent analog, **6b**, triggered 35.29% total apoptosis in MCF-7 cells and stopped the cell cycle at the G2/M phase in comparison with the untreated cells’ 6.81% apoptosis. It was shown that the in vitro investigations were represented and validated by in silico ADMET and docking simulation with EGFR.

## Experimental

### Synthetic procedures

#### Materials and methods

All melting points were measured with an electro-thermal IA 9100 device (Shimadzu, Kyoto, Japan) and are uncorrected. The produced compounds’ anticancer properties were tested at Mansoura University’s Pharmacy Faculty. Using KBr discs, IR spectra were performed using a JASCO FT/IR 6100 Japan spectrometer. In the deuterated solvent DMSO-d6 at 25 °C, BRUKER and JEOL spectrometers operate at 400 MHz for ^1^HNMR spectra and 100 MHz for ^13^CNMR spectra. To illustrate signal multiplicity, coupling constants (*J*) were shown in Hertz (Hz), and NMR spectra were displayed as chemical shifts (ppm) against tetramethyl silane (TMS) as an internal standard. Agilent was used to record mass spectra. All of the raw ingredients needed to synthesize each chemical were purchased from Sigma Aldrich. Chemicals and solvents including ethanol, di methyl formamide, and glacial acetic acid were bought from Merck and used as original. Silica gel plates with aluminum back were utilized to check the reaction progress and completion.

### General synthetic procedure and spectral studies

Full spectral characterization data (IR, ^1^H NMR, ^13^C NMR, and mass spectra) for all synthesized compounds are provided in the Supplementary Information (Figures **S1**–S**49**).

*S*-(4-Oxo-3-phenyl-3,4-dihydroquinazolin-2-yl) 2-chloroethanethioate (**1**).

A solution of 2-mercapto-3-phenylquinazolin-4(3*H*)-one (10 mmol, 2.54 g) was dissolved in 10 mL of dry dichloromethane (DCM) in a 100 mL round-bottom flask under a nitrogen atmosphere, with stirring in an ice bath maintained at 0–5 °C. Triethylamine (12 mmol, 1.21 mL) was added slowly to neutralize the hydrogen chloride generated during the reaction. After stirring for 10 min, chloroacetyl chloride (12 mmol, 0.95 mL) was added dropwise, keeping the temperature below 5 °C. The reaction mixture was then stirred at room temperature for 4 h, with reaction progress monitored by thin-layer chromatography (TLC) using an ethyl acetate:Hexane (3:1) solvent system. Upon completion, the mixture was poured into 50 mL of cold water and stirred for 15 min. The organic phase was sequentially washed with 20 mL of 10% sodium chloride solution to remove inorganic impurities, followed by 20 mL of 5% sodium bicarbonate solution to neutralize residual acid. The crude was recrystallized with ethanol to yield white to pale yellow crystalline solid. Yield: 77%; m.p. 298–300 °C. ^1^H-NMR (DMSO-*d6*, 400 MHz,) δ: 4.22 (s, 2H, CH_2_), 7.56 − 8.08 (m, 9H, Ar–H) ppm. ^13^C-NMR (DMSO-*d6*, 100 MHz) δ: 49.33 (C, CH_2_), 115.71 − 139.58 (11C, Ar–C), 147.12 (1C, C quinazolin-4-one -N), 159.78 (C, C=N), 166.68 (C, C=O amide), 176.02 (C, C=O-S) ppm. IR (KBr) cm^-1^ν_max_: 3081, 3026 (CH-Ar), 2936 (CH-aliphatic), 1710 (C=O-S), 1659 (C=O amide), 1594 (C=N), 1495 (C=C Ar). Anal. calcd for C_21_H_16_N_2_O_3_S (376.09): C 58.10, H 3.35, N 8.47; found C 58.12, H 3.30, N 8.41.

2- [(4-Acetyl-5-methylfuran-3-yl)thio]-3-phenylquinazolin-4(3*H*)-one** (2**).

In a 100 mL round-bottom flask, **1** (10 mmol, 3.30 g) was dissolved in 10 mL of dry DMF to create a solution. Anhydrous potassium carbonate (K₂CO₃) (15 mmol, 2.07 g) was added as a base to this solution. To produce the reactive thiolate anion, the reaction mixture was agitated for half an hour at room temperature. After that, the reaction mixture was agitated for six h at 60 °C under reflux while acetylacetone (10 mmol, 1.12 mL) was added dropwise. TLC (ethyl acetate: Hexane, 3:1) was used to track the reaction’s progress. In order to precipitate the result, the mixture was cooled to room temperature once the reaction was finished and then added to 50 ml of ice-cold water. After being recovered by vacuum filtration, the solid was cleaned of contaminants using cold ethanol. By recrystallizing the crude product from ethanol, the pure chemical was obtained as an orange crystalline solid. Yield: 70%; m.p. > 300 °C. ^1^H-NMR (DMSO-*d6*, 400 MHz) δ: 2.71, 2.87 (s, 6H, 2CH₃), 7.24 (s, 1H, Ar–CH furyl), 7.27–7.94 (m, 9H, Ar–H) ppm. ^13^C-NMR (DMSO-*d6*, 100 MHz) δ: 12.11, 27.33 (2C, 2CH₃), 115.71–147.12 (15C, Ar–C), 150.00 (1C, C=N), 159.78 (1C, Ar–C furyl-CH₃), 166.68 (1C, C=O amide), 180.00 (1C, C=O-furyl). IR (KBr) cm⁻^1^ν_max_: 3090 (CH-Ar), 2977, 2930 (CH-alipha), 1722 (C=O-furyl), 1661 (C=O amide), 1586 (C=N), 1531 (C=C Ar). Anal. Calcd for C_21_H_16_N_2_O_3_S (376.09): C 67.01, H 4.28, N 7.44; found: C 67.02, H 4.30, N 7.41.

2-{{{4-[3-(4-Aryl)acryloyl]-5-methylfuran-3-yl}thio}}-3-phenylquinazolin-4(3*H*)-on (**3a-d**)

*General procedure* To a solution of sodium ethoxide (20 mL), **2** (10 mmol, 3.76 g) and substituted aldehydes (10 mmol) (*p*-chlorobenzaldehyde 1.41 g, *p*-florobenzaldehtyde 1.07 mL, *p-*methoxy benzaldehyde 1.21 mL, and furfuraldehtyde 0.83 mL) were added. The reaction mixture was stirred for 12 h. The progress of the reaction was monitored by TLC (ethyl acetate: hexane, 3:1). Once the reaction was completed, The reaction mixture was poured onto crushed ice and then acidified with HCl. The resulting precipitate was filtered off, dried well, and recrystallized from ethanol.

2-{{{4-[3-(4-Chlorophenyl)acryloyl]-5-methylfuran-3-yl}thio}}-3-phenylquinazolin-4(3*H*)-one (**3a**)

Colour: Off-White crystalline solid, yield: 75%; m.p. 220–222 °C. ^1^H-NMR (DMSO-d6, 400 MHz,) δ: 2.58 (s, 3H, CH3), 6.95, 7.12 (d, j = 8.2 Hz, 2H, 2CH), 7.56–8.08 (m, 14H, Ar–H) ppm. ^13^C-NMR (DMSO-d6, 100 MHz) δ: 12.19 (C, CH3), 114.78–140.32 (23C, Ar–C), 150.65 (C, C=N), 160.29 (1C, Ar–C furyl-CH₃), 162.70 (1C, C=O amide), 176.53 (1C, C=O-furyl). IR (KBr) cm-1 νmax: 3100, 3050 (CH-Ar), 2926 (CH-alipha), 1690 (C=O α,β-unsaturated ketone), 1655 (C=O amide), 1583 (C=N), 1527 (CH=CH alkene), 1498 (C=C Ar). MS (70 eV) m/z (%): 498 (M + , 45), 175 (100), 500 (M + 2, 15). Anal. calcd for C_28_H_19_ClN_2_O_3_S (498.08): C 67.40, H 3.84, N 5.61; found: C 67.48, H 3.85, N 5.62.

2-{{{4-[3-(4-Fluorophenyl)acryloyl]-5-methylfuran-3-yl}thio}}-3-phenylquinazolin-4(3*H*)-one (**3b**)

Colour: Black crystalline solid, yield: 69%; m.p. 269–271 °C. ^1^H-NMR (DMSO-d6, 400 MHz,) δ: 2.51 (s, 3H, CH3), 6.81, 7.28 (d, j = 16.8 Hz 2H, 2CH), 7.30–7.98 (m, 14H, Ar–H) ppm. ^13^C-NMR (DMSO-d6, 100 MHz) δ: 12.30 (C, CH3), 116.17–140.07 (22C, Ar–C), 153. 10 (C, C=N), 157.55 (C, Ar–C furyl-CH₃), 160.30 (C, C=O amide), 165.60 (C, Ar–C-F), 176.54 (C, C=O-furyl). IR (KBr) cm-1 νmax: 3093, 3069 (CH-Ar), 2927 (CH-alipha), 1688 (C=O α,β-unsaturated ketone), 1651 (C=O amide), 1596 (C=N), 1533 (CH=CH alkene), 1467 (C=C Ar). Anal. calcd for C28H19FN2O3S (482.11): C 69.70, H 3.97, N 5.81; found: C 69.71, H 3.95, N 5.80.

2-{{{4-[3-(4-Methoxyphenyl)acryloyl]-5-methylfuran-3-yl}thio}}-3-phenylquinazolin-4(3*H*)-one (**3c**)

Colour: Pale yellow crystalline solid, yield ield: 70%; m.p. 298–300 °C. ^1^H-NMR (DMSO-d6, 400 MHz,) δ: 2.50 (s, 3H, CH3), 3.95 (s, 3H, OCH3), 6.92, 7.28 (d, j = 7.4 Hz, 2H, 2CH), 7.30–7.98 (m, 14H, Ar–H) ppm. 13C-NMR (DMSO-d6, 100 MHz) δ: 12.33 (C, CH3), 55.30 (C, OCH3), 116.17–140.08 (22C, Ar–C), 153.60 (C, Ar–C-OCH3), 154.39 (C, C=N), 154.47 (1C, Ar–C furyl-CH₃), 160.29 (C, C=O amide), 176.54 (C, C=O-furyl). IR (KBr) cm-1 νmax: 3093, 3069 ( CH-Ar), 2998, 2939 (CH-alipha), 1687 (C=O α,β-unsaturated ketone), 165 7(C=O amide), 1597 (C=N), 1580 (CH=CH alkene), 1558 (C=C Ar). Anal. calcd for C29H22N2O4S (494.57): C 70.43, H 4.48, N 5.66, found: C 72.43, H 4.52, N 5.61.

2-{{{4-[3-(Furan-2-yl)acryloyl]-5-methylfuran-3-yl}thio}}-3-phenylquinazolin-4(3*H*)-one (**3d**)

Colour: Brown crystalline solid, yield: 77%; m.p. > 300 °C. 1H-NMR (DMSO-d6, 400 MHz,) δ: 2.50 (s, 3H, CH3), 6.90, 7.28 (d, j = 8.5 Hz, 2H, 2CH), 7.30–7.97(m, 13H, Ar–H) ppm. 13C-NMR (DMSO-d6, 100 MHz) δ: 14.10 (C, CH3), 119.30–145.45 (21C, Ar–C), 152.07 (C, C=N), 154.30 (C, Ar–C furyl-CH₃), 160.57(1C, C=O amide), 176.54 (1C, C=O-furyl). IR (KBr) cm-1 νmax: 3091, 3071 (CH-Ar), 2971 (CH-alipha), 1686 (C=O α,β-unsaturated ketone), 1655 (C=O amide), 1637 (C=N), 1588 (CH=CH alkene), 1560 (C=C Ar). MS (70 eV) m/z (%): 454 (M + , 11), 102 (100). Anal. calcd for C26H18N2O4S (454.10): C 68.71, H 3.99, N 6.16: found: C 69.71, H 4.00, N 6.15.

Ethyl 4′-fluoro-5-{2-methyl-4-[(4-oxo-3-phenyl-3,4-dihydroquinazolin-2-yl)thio]furan-3-yl}-3-oxo-1,2,3,6-tetrahydro-[1,1′-biphenyl]-2-carboxylate (**4**)

A solution was prepared by dissolving **3b** (10 mmol, 4.82 g) in 10 mL of dry ethanol in a 100 mL round-bottom flask under a nitrogen atmosphere. Ethyl acetoacetate (10 mmol, 1.08 mL) was added dropwise to the reaction mixture, followed by triethylamine (2–3 drops, catalytic amount) to facilitate enamine formation. After 30 min of stirring at room temperature, the reaction mixture was heated for 6 h at 80 °C under reflux. TLC (ethyl acetate: Hexane, 33:1) was used to track the reaction’s development. In order to precipitate the result, the mixture was cooled to room temperature once the reaction was finished and then added to 50 ml of ice-cold water. To get rid of contaminants, the solid was collected using vacuum filtration and then cleaned with cold ethanol. By recrystallizing the crude product from ethanol, a yellow crystalline solid was produced. Yield:70%; m.p. > 300 °C. ^1^H-NMR (DMSO-*d6*, 400 MHz) δ: 1.22 (t, *j* = 7.2 Hz, 3H, CH_3_ ester), 2.27 (d, *j* = 8.2 Hz, 2H, CH_2_ cyclohexenone), 2.88 (s, 3H, CH_3_-furyl), 3.31 (d, *j* = 9.2 Hz, 1H, CH cyclohexenone-ester), 3.79 (q, *j* = 7.8 Hz, 1H, CH cyclohexenone -Ph-F), 4.24 (q, *j* = 10.01 Hz, 2H, CH_2_ ester), 7.25–7.96 (m, 15H, 1H, CH cyclohexenone-C=O, 14 Ar–H) ppm. ^13^C-NMR (DMSO-*d6*, 100 MHz) δ: 15.57 (2C, 2CH₃), 27.27 (C, CH cyclohexenone-Ph-F), 34.27 (C, CH₂ cyclohexenone), 61.27 (C, CH_2_ ester), 65.57 (C, CH cyclohexenone-ester), 115.84–139.72 (25C, Ar–C), 152.11 (C, C=N), 152.54 (C, Ar–C furyl-CH_3_), 159.64 (C, Ar C-F), 160.03 (C, C=O amide), 176.11 (C, C=O ester), 192.94 (C, C=O cyclohexenone) ppm. IR (KBr) cm^-1^ ν_max_: 3080, 3028 (CH-Ar), 2989, 2945, 2920, 2873 (CH-aliphatic), 1728 (C=O cyclohexanone), 1686 (C=O ester), 1666 (C=O amide), 1619 (C=N), 1599 (C=C Ar). Anal. Calcd for C_34_H_27_FN_2_O5_S_ (594.66): C 68.67, H 4.58, N 4.71; found C 68.77, H 4.60, N, 4.71.

4-(4-Methoxyphenyl)-6-{2-methyl-4-[(4-oxo-3-phenyl-3,4-dihydroquinazolin-2-yl)thio]furan-3-yl}nicotinonitrile (**5**)

A solution of **3c** (10 mmol, 4.94 g) was dissolved in 10 mL of ethanol in a 100 mL round-bottom flask under a nitrogen atmosphere. To this, malononitrile (10 mmol, 0.66 mL) was added, followed by the dropwise addition of triethylamine (2–3 drops, catalytic amount) to promote cyclization. The reaction mixture was stirred at room temperature for 30 min and then refluxed at 80 °C for 6 h. Reaction progress was monitored by thin-layer chromatography (TLC) using ethyl acetate:hexane (3:1) as the mobile phase. After completion, the mixture was cooled to room temperature and poured into 50 mL of ice-cold water to precipitate the product. The resulting solid was collected by vacuum filtration, washed with cold ethanol to remove impurities, and purified by recrystallization from ethanol to yield a black crystalline solid. Yield: 68%; m.p. 277–279 °C. ^1^H-NMR (DMSO-*d6*, 400 MHz) δ: 2.50 (s, 3H, CH₃), 3.94 (s, 3H, OCH₃), 7.06–8.02 (m, 14H, Ar–H), 9.00, 9.28 (s, 2H, 2CH nicotinonitrile ring) ppm. ^13^C-NMR (DMSO-*d6*, 100 MHz) δ: 12.39 (C, CH₃), 55.10 (C, OCH₃), 100.00 (C, Ar–C furyl-S), 104.90 (C, C pyridine-CN), 114.79 (C, CN), 115.70–140.08 (22C, Ar–C), 150.67 (C, CH pyridine-N), 153.62 (C, Ar–C-OCH_3_), 155.10 (C, C=N pyrimidine), 157.00 (1C, Ar–C furyl-CH₃), 157.08 (C, C=N pyridine), 160.30 (C, C=O amide) ppm. IR (KBr) cm⁻^1^ ν_max_: 3089, 3023 (CH-Ar), 2923 (CH-aliphatic), 2892 (OCH₃), 2221 (CN), 1654 (C=O), 1623, 1603 (2C=N), 1581 (C=C Ar). MS (70 eV) *m*/*z* (%): 542 (M + , 10), 339 (100). Anal. Calcd for C_32_H_22_N_4_O_3_S (542.14): C 70.83, H 4.09, N, 10.33; found C 70.80, H 4.10, N 10.35.

2-{{{4-[4-(4-Fluorophenyl)-2,3,4,5-tetrahydro-1*H*-benzo[b][1,4]diazepin-2-yl]-5-methylfuran-3-yl}thio}}-3-phenylquinazolin-4(3*H*)-one (**6a**)

In a 100 mL round-bottom flask with a nitrogen atmosphere, **3b** (10 mmol, 4.82 g) was dissolved in 10 mL of dry ethanol to create a solution. To encourage the cyclization reaction, o-phenylenediamine (10 mmol, 1.08 g) and triethylamine (2–3 drops, catalytic quantity) were added to the reaction mixture. After 30 min of stirring at room temperature, the reaction mixture was heated under reflux for 6–8 h at 80 °C. TLC (ethyl acetate: Hexane, 3:1) was used to track the reaction’s progress. Upon completion, the reaction mixture was cooled to room temperature and poured into 50 mL of ice-cold water to precipitate the product. The solid was collected by vacuum filtration and washed with cold ethanol to remove impurities. The crude product was purified by recrystallization from ethanol, yielding a bright black crystalline solid. Yield: 77%; m.p. > 300 °C. ^1^H-NMR (DMSO-*d*_*6*_, 400 MHz,) *δ*: 2.74 (s, 3H, CH_3_), 2.90 (d,d, *j* = 9.6 Hz ,2H, CH_2_ diazepine), 3.59 (t, *j* = 12.5 Hz, 2H, 2CH diazepine), 6.98 (brs, 2H, 2NH exchangeable), 7.28–7.98 (m, 18H, Ar–H) ppm. ^13^C-NMR (DMSO-d_6_, 100 MHz) *δ*: 12.23 (C, CH_3_), 39.35 (C, CH_2_ diazepine), 54.33 (2C, 2CH diazepine), 114.80–140.30 (28C, Ar–C), 160.30 (1C, C=O amide), 164. 44 (C, C=N diazepine), 168.54 (1C, Ar–C-F) ppm. IR (KBr) cm^-1^ ν_max_: 3120, 3155 (2NH), 3099, 3064 (CH-Ar), 2974, 2933, 2920, 2870 (CH-aliphatic), 1665 (C=O), 1619 (C=N), 1550 (C=C Ar). Anal. Calcd for C_28_H_19_FN_2_O_3_S (482.11): C 69.70, H 3.97, N 5.81; found C 69.71, H 3.97, N 5.80.

2-((4-(4-(Furan-2-yl)-2,3,4,5-tetrahydro-1H-benzo[b][1,4]diazepin-2-yl)-5-methylfuran-3-yl)thio)-3-phenylquinazolin-4(3H)-one (**6b**)

A solution was prepared by dissolving** 3d** (10 mmol, 4.54 g) in 10 mL of dry ethanol in a 100 mL round-bottom flask under a nitrogen atmosphere. o-Phenylenediamine (10 mmol, 1.08 g) was added to the reaction mixture, followed by the addition of triethylamine (2–3 drops, catalytic amount) to promote the cyclization reaction. The reaction mixture was stirred at room temperature for 30 min, then heated under reflux at 80 °C for 6–8 h. The progress of the reaction was monitored by TLC (ethyl acetate: hexane, 3:1). Upon completion, the reaction mixture was cooled to room temperature and poured into 50 mL of ice-cold water to precipitate the product. The solid was collected by vacuum filtration and washed with cold ethanol to remove impurities. The crude product was purified by recrystallization from ethanol, yielding a bright pale brown crystalline solid. Yield: 65%; m.p. > 300 °C. ^1^H-NMR (DMSO-*d*_*6*_, 400 MHz,) *δ*: 2.55 (s, 3H, CH_3_), 2.90 (d,d, *j* = 6.2 Hz , 2H, CH_2_ diazepine), 3.05, 3.09 (t, *j* = 7.1 Hz, 2H, 2CH diazepine), 3.41 (brs, 2H, 2NH exchangeable), 6.61–7.97 (m, 17H, Ar–H) ppm. IR (KBr) cm^-1^ ν_max_: 3227, 3170 (2NH), 3085, 3064 (CH-Ar), 2949, 2885 (CH-aliphatic), 1663 (C=O), 1613 (C=N), 1527 (C=C Ar). MS (70 eV) *m*/*z* (%): 546 (M + , 10), 253 (100). Anal. Calcd for C_32_H_26_N_4_O_3_S (546.17): C 70.31, H 4.79, N 10.25; found C 70.30, H 4.84, N 10.21.

2-{{{4-[5-(4-Chlorophenyl)-4,5-dihydro-1*H*-pyrazol-3-yl]-5-methylfuran-3-yl}thio}}-3-phenylquinazolin-4(3*H*)-one (**7**)

A mixture of 3a (10 mmol, 4.89 g) and hydrazine hydrate (25 mmol, 1.25 mL, 99%) was dissolved in absolute ethanol (20 mL) in catalytic amount of gl. AcOH was refluxed at 80–90 °C for 4–6 h under continuous stirring. The reaction progress was monitored by thin-layer chromatography (TLC) using ethyl acetate:Hexane (7:3) as the mobile phase. Upon completion, the reaction mixture was cooled to room temperature and poured into crushed ice (50 mL) with vigorous stirring. The precipitated solid was filtered, washed with cold ethanol, and dried in an oven at 60 °C. The crude product was purified by recrystallization from ethanol-chloroform mixture (3:1), affording the target compound as a yellow to light brown crystalline solid. Yield: 80%; m.p. 212–214 °C. ^1^H-NMR (DMSO-*d6*, 400 MHz,) δ: 2.52 (s, 3H, CH_3_), 3.89 (d, *j* = 5.2 Hz, 2H, CH_2_ pyrazole), 4.40 (t, *j* = 8.5 Hz, 1H, CH pyrazole), 5.75–8.41 (m, 14H, Ar–H), 9.90 (brs, 1H, NH exchangeable) ppm. ^13^C-NMR (DMSO-*d6*, 100 MHz) δ: 12.34 (C, CH_3_), 46.69 (C, CH_2_ pyrazole), 55.65 (C, CH pyrazole), 101.98 (C, C furyl-pyrazole), 111.08–144.44 (20C, Ar–C), 151.00 (C, C=N pyrazole), 153.33 (C, C furyl-CH_3_), 160.00 (C, C=N pyrimidine), 161.92 (C, C=O) ppm. IR (KBr) cm^-1^ ν_max_: 3416 ( NH), 3061, 3027 (CH-Ar), 2923 (CH-aliphatic), 1664 (C=O), 1614, 1569 (2C=N), 1535 (C=C Ar). Anal. Calcd for C_28_H_21_ClN_4_O_2_S (512.11): C 65.56, H 4.13, N 10.92; found C 65.55, H 4.19, N 10.92A.

5-(4-Chlorophenyl)-3-{2-methyl-4-[(4-oxo-3-phenyl-3,4-dihydroquinazolin-2-yl)thio]furan-3-yl}-4,5-dihydro-1*H*-pyrazole-1-carbothioamide (**8a**)

A solution was prepared by dissolving **3a** (10 mmol, 4.89 g) in 10 mL of dry ethanol in a 100 mL round-bottom flask under a nitrogen atmosphere. thiosemicarbazide (0.91 g, 10 mmol) was added to the reaction mixture, followed by the addition of triethylamine (2–3 drops, catalytic amount) to facilitate cyclization. The reaction mixture was stirred at room temperature for 30 min, then heated under reflux at 80 °C for 6–8 h. The progress of the reaction was monitored by TLC (ethyl acetate: hexane, 3:1).Upon completion, the reaction mixture was cooled to room temperature and poured into 50 mL of ice-cold water to precipitate the product. The solid was collected by vacuum filtration and washed with cold ethanol to remove impurities. The crude product was purified by recrystallization from ethanol, yielding a yellow crystalline solid. Yield: 62%; m.p. 298–300 °C. ^1^H-NMR (DMSO-*d6*, 400 MHz,) δ: 2.50 (s, 3H, CH_3_), 3.52 (d, *j* = 8.1 Hz, 2H, CH_2_ pyrazole), 3.80 (t, *j* = 6.5 Hz, 1H, CH pyrazole), 7.24–8.31 (m, 14H, Ar–H), 9.14 (brs, 2H, NH_2_ exchangeable) ppm. ^13^C-NMR (DMSO-*d*_*6*_, 100 MHz) *δ*: 12.39 (C, CH_3_), ,46.10 (C, CH_2_ pyrazole), 72.25 (1C, CH–N pyrazole), 114.79–140.29 (23C, Ar–C), 150.67 (C, C furyl-S), 160.29 (1C, C=O amide), 176.53 (C, C=S) ppm. IR (KBr) cm^-1^ ν_max_: 3492, 3398 (NH_2_), 3088, 3061 (CH-Ar), 2952, 2925 (CH-aliphatic), 1655 (C=O), 1611, 1588 (2C=N), 1566 (C=C Ar), 1302 (C=S). MS (70 eV) *m*/*z* (%): 571(M + , 10), 339 (100). Anal. Calcd for C_29_H_22_ClN_5_O_2_S_2_ (571.09): C, 60.88; H, 3.88; Cl, 6.20; N, 12.24; O, 5.59; S, 11.21%. found C, 60.90; H, 3.90; Cl, 6.20; N, 12.24; O, 5.59; S, 11.21%.

(4-Fluorophenyl)-3-{2-methyl-4-[(4-oxo-3-phenyl-3,4-dihydroquinazolin-2-yl)thio]furan-3-yl}-4,5-dihydro-1*H*-pyrazole-1-carbothioamide (**8b**)

A solution of **3b** (10 mmol, 4.82 g) was prepared by dissolving it in 10 mL of dry ethanol in a 100 mL round-bottom flask under a nitrogen atmosphere. To this, thiosemicarbazide (0.91 g, 10 mmol) was added, followed by the dropwise addition of triethylamine (2–3 drops, catalytic amount) to promote cyclization. The mixture was stirred at room temperature for 30 min, then heated under reflux at 80 °C for 6–8 h. The reaction progress was monitored by thin-layer chromatography (TLC) using ethyl acetate:hexane (3:1) as the eluent. Upon completion, the reaction mixture was cooled to room temperature and poured into 50 mL of ice-cold water to induce precipitation of the product. The solid was collected by vacuum filtration, washed with cold ethanol to remove impurities, and purified by recrystallization from ethanol, yielding a yellow crystalline solid. Yield: 775; m.p. 298–300 °C. ^1^H-NMR (DMSO-*d*_*6*_, 400 MHz,) *δ*: 2.31 (s, 3H, CH_3_), 3.59 (d, *j* = 5.2 Hz , 2H, CH_2_), 4.22 (t, *j* = 8.6 Hz, 1H, CH), 7.28–7.97 (m, 14H, Ar–H), 10.59 (brs, 2H, NH_2_ exchangeable) ppm.^13^C-NMR (DMSO-d_6_, 100 MHz) *δ*: 12.39 (1C, CH_3_), 46.04 (1C, CH_2_), 75.05 (1C, CH–N), 116.17–140.30 (21C, Ar–C), 157.59, 164.34 (2C, 2C=N), 160.30 (1C, C=O), 168.44 (1C, C-F), 179.59 (1C, C=S)ppm. IR (KBr) cm^-1^ ν_max_: 3488, 3460 cm^-1^ (ν NH_2_), 3082, 3067 cm^-1^ (ν CH-Ar), 2950, 2924 cm^-1^ (ν CH-aliphatic), 1657 cm^-1^ (ν C=O), 1615, 1596 cm^-1^ (ν 2C=N), 1582 cm^-1^ (ν C=C Ar), 1264 cm^-1^ (ν C=S). Anal. Calcd for C_29_H_22_FN_5_O_2_S_2_ (555.12): C 62.69, H 3.99, N 12.60; found C 62.70, H 4.02, N, 12.60.

2-{{{4-[5-(4-Chlorophenyl)-1-(4-chlorothiazol-2-yl)-4,5-dihydro-1*H*-pyrazol-3-yl]-5-methylfuran-3-yl}thio}}-3-phenylquinazolin-4(3*H*)-one (**9**)

A solution was prepared by dissolving **8a** (10 mmol, 5.72 g) in 10 mL of dry (DCM) in a 100 mL round-bottom flask under a nitrogen atmosphere. The reaction mixture was cooled to 0–5 °C using an ice bath. Chloroacetyl chloride (10 mmol, 0.8 mL) was added dropwise to the reaction mixture with continuous stirring. After complete addition, triethylamine (2–3 drops, catalytic amount) was added to neutralize the evolving HCl and to facilitate nucleophilic substitution. The reaction mixture was stirred at 0–5 °C for 30 min, then allowed to warm to room temperature and refluxed further for 6–8 h at 100 °C . The reaction progress was monitored by TLC (ethyl acetate: Hexane, 3:1). After completion, the reaction mixture was poured into 50 mL of ice-cold water with stirring to precipitate the product. The resulting solid was collected by vacuum filtration and washed thoroughly with cold ethanol to remove impurities. The crude product was purified by recrystallization from ethanol, yielding a yellow crystalline solid. Yield: 89%; m.p. 228–230 °C. ^13^C-NMR (DMSO-*d*_*6*_, 100 MHz) *δ*: 13.39 (C, CH_3_), 44.10 (C, CH_2_ pyrazole), 60.69 (1C, CH pyrazole), 114.79–140.08 (24C, Ar–C), 150.76 (1C, C=N pyrimidine), 160.30 (C, C=O amide), 162.70 (C, C=N pyrazole), 176.54 (C, C=N thiazole) ppm. IR (KBr) cm^-1^ ν_max_: 3083, 3060, 3025 (CH-Ar), 2950, 2925, 2830 (CH-aliphatic), 1669 (C=O), 1642, 1615, 1597 (3C=N), 1569 (C=C Ar). MS (70 eV) *m*/*z* (%):629 (M + , 10), 253(100). Anal. Calcd for C_31_H_21_Cl_2_N_5_O_2_S_2_ (629): C 59.05, H 3.36, N11.11; found C 60.00, H 3.42, N 1.11.

5-(4-Fluorophenyl)-*N*-[(5-formylthiophen-2-yl)methylene]-3-(2-methyl-4-{[4-oxo-3-phenyl-3,4-dihydroquinazolin-2-yl)thio]furan-3-yl}-4,5-dihydro-1*H*-pyrazole-1-carbothioamide (**10**)

A solution was prepared by dissolving **8b** (10 mmol, 5.55 g) in 15 mL of absolute ethanol in a 100 mL round-bottom flask under a nitrogen atmosphere. To this solution, thiophene-2,5-dicarbaldehyde (10 mmol, 1 mL) was added dropwise with continuous stirring. A catalytic amount of glacial acetic acid (2–3 drops) was added to promote Schiff base formation. The reaction mixture was stirred at room temperature for 30 min, then heated under reflux at 80 °C for 6–8 h. The progress of the reaction was monitored by TLC (ethyl acetate: Hexane, 3:1). After completion, the reaction mixture was cooled to room temperature and poured into 50 mL of ice-cold water with stirring to induce precipitation. The solid product was collected by vacuum filtration and washed thoroughly with cold ethanol to remove impurities. The crude product was purified by recrystallization from ethanol, yielding a yellow crystalline solid. Yield: 86%; m.p. 296–298 °C. ^1^H-NMR (DMSO-*d*_*6*_, 400 MHz,) *δ*: 2.54 (s, 3H, CH_3_), 3.61 (d, *j* = 10.4 Hz, 2H, CH_2_ pyrazole), 3.81 (t, *j* = 16.3 Hz ,1H, CH pyrazole), 7.28–7.95 (m, 16H, Ar–H), 7.97 (s, 1H, CH=N), 9.51 (s, 1H, CH aldehyde) ppm. ^13^C-NMR (DMSO-*d*_*6*_, 100 MHz) *δ*: 12.33 (C, CH_3_), 46.04 (C, CH_2_ pyrazole), 72.25 (C, CH pyrazole), 116.17–140.30 (26C, Ar–C), 157.50 (C, CH-S), 160.30 (1C, C=O amide), 168.38 (1C, Ar–C-F), 185.59 (1C, C=O aldehyde), 190.00 (1C, C=S) ppm. IR (KBr) cm^−1^ ν_max_: 3090, 3067 (CH-Ar), 2977 (CH-alipha), 2737, 2677 (CHO), 1710, 1644 (2C=O), 1602, 1528 (3C=N), 1474 (C=C Ar), 1267 (C=S).

### Biological evaluation

#### Cytotoxic activity

Following the published protocol^75–77^, the cytotoxic effects of the newly synthesized quinazolinones **1**–**10** were assessed in vitro using the MTT test method on human liver HepG-2, breast MCF-7, colorectal HCT-116 cancer cell lines, and normal diploid cell line WI-38. There were further details in the supplemetary file.

#### Inhibitory assessment against EGFR and VEGFR-2 activities

Using erlotinib as a reference, the potential quinazolinones **1**–**10** were evaluated in vitro for their inhibitory effect on EGFR activity in accordance with the previously described methodology^78,79^. The supplementary file contained more details.

#### Detection of cell cycle analysis and apoptosis of compound 6b

Using flow cytometry, the investigation of cell cycle analysis and apoptosis was described^79,81^. After applying quinazolinone-furan-benzo[*b*][1,4]diazepine **6b** to MCF-7 cells for 24 h, the cells were incubated at 37 °C. The supplemental file contained more information.

#### The effect of compound 6b on MCF-7 cell levels of Bax, Bcl-2 and p53

For the promising quinazolinone-furan-benzo[*b*][1,4]diazepine 6b, the Bax, Bcl-2, and p53 levels in MCF-7 cells were elucidated using the previously disclosed method.

#### Molecular docking simulation

Computational docking simulation has contributed to facilitate the rationalization of biological findings more easily. The EGFR (PDB code: 1M17) active site has been identified to include quinazolinones **6b**, **8a**, and **10** where Auto Dock Vina 4.2 was utilized^86,87^. Complete explanations can be found in the supporting documentation.

#### Quantum chemical calculations

Quantum mechanical calculations play a crucial role in understanding molecular properties and reaction mechanisms. In this study, density functional theory (DFT) calculations were performed using the B3LYP functional and the 6-31G(d) basis set to optimize molecular structures and analyze electronic properties. All calculations were conducted using Gaussian software, and further details are provided in the supporting information.

## Supplementary Information

Supplementary Information.

## Data Availability

All relevant data generated or analyzed during this study are included in the (supplementary information file) uploaded with the manuscript.
