# Supplementary material for: Design, antiproliferative potency, and in silico studies of novel 5-methylfuran-3-yl)thio)-3-phenylquinazolin-4(3H)-one based derivatives as potential EGFR inhibitors
Source: Sci Rep. 2025 Jul 31;15:27992. doi: 10.1038/s41598-025-12140-1 (PMC12314051; doi:10.1038/s41598-025-12140-1)
Supplement: Supplementary file 1 — Supplementary Information. [file 41598_2025_12140_MOESM1_ESM.docx]

**Supporting Information**

Design, Antiproliferative Potency, and In Silico Studies of Novel 5-Methylfuran-3-yl)thio)-3-phenylquinazolin-4(3*H*)-one Based Derivatives as Potential EGFR Inhibitors

**Sara M. Soliman^1^, Adel A.-H. Abdel-Rahman^1*^, Eman S. Nossier^2,3*^, Modather F. Hussein^4^,**

**Amr Sabry^5^, Hagar S. El-Hema^6*^**

^1^ Chemistry Department, Faculty of Science, Menoufia University, Shebin El-Kom 32511, Egypt; saramouse1555@gmail.com; (S.M.S.); adelnassar63@yahoo.com (A.A.-H.A.-R..)

^2*^ Department of Pharmaceutical Medicinal Chemistry and Drug Design, Faculty of Pharmacy (Girls), Al-Azhar University, Cairo, 11754, Egypt; dr.emannossier@gmail.com (E.S.N.)

^3*^ The National Committee of Drugs, Academy of Scientific Research and Technology, Cairo, 11516, Egypt

^4^ Chemistry Department, College of Science, Jouf University, Sakaka, Aljouf 72341, Saudi Arabia; mfhussin@ju.edu.sa (M.F.H..)

^5^ Department of Pharmaceutical Manufacturing, Faculty of Pharmacy, MUST University, Giza 3237101, Egypt; 79365@must.edu.eg (A.S.)

^6*^ Basic Science Department (Chemistry), Thebes Higher Institute for Engineering, Thebes Academy, Maadi 11434, Egypt; hagarsabry.23@yahoo.com (H.S.E.-H.)

*Corresponding authors: ([hagarsabry.23@yahoo.com](mailto:hagarsabry.23@yahoo.com));(adelnassar63@yahoo.com);([dr.emannossier@gmail.com](mailto:dr.emannossier@gmail.com)).

**CONTENTS:**

1. **Experimental**
   1. **Chemistry**

- **Figure S1–S49**. Copies of ^1^H NMR, ^13C^ NMR, IR, and mass spectra of Compounds.

**1.2. Biological evaluation docking study, and Quantum chemical calculations study.**

- **Table S1.** The percentage cytotoxicity of quinazolinones **1**–**10** upon human tumor HepG-2, HCT-116, MCF-7 and normal WI-38 cell lines at different concentrations according to the MTT assay.
- **Table S2.** Cell cycle analysis after 48 h incubation with compound **6b**
- **Table S3.** Apoptosis induction analysis within MCF-7 cells treated with compound **6b**
- **Table S4.** HOMO, LUMO, and ESP energies for the compounds.
- **Figure S2. A** & **B** diagrams illustrated 2D and 3D binding features of the original ligand erlotinib within the active site of EGFR (PDB code: 1M17).

**Copies of ^1^H NMR, ^13^C NMR, IR, Mass spectra of Compounds**

**Characterization of Compound 1:**

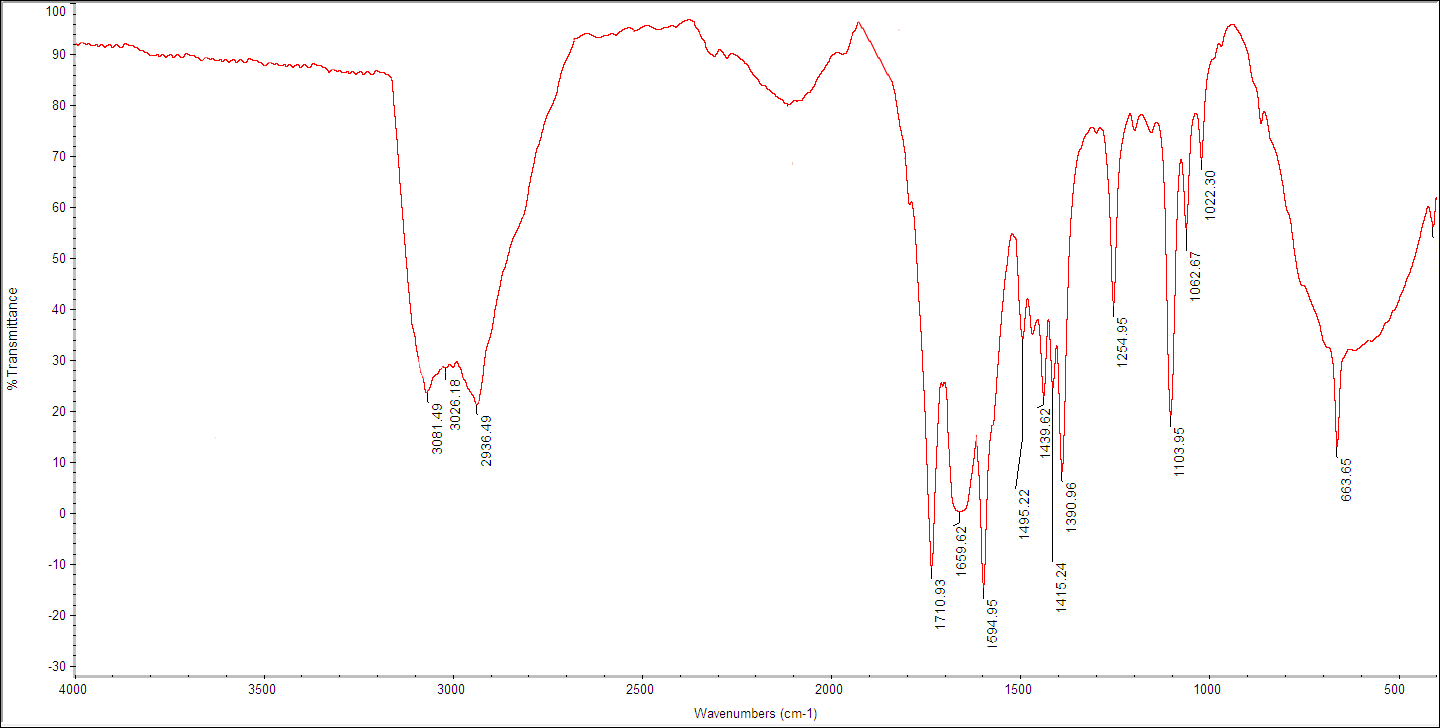


**Figure S1. IR spectrum of Compound 1**


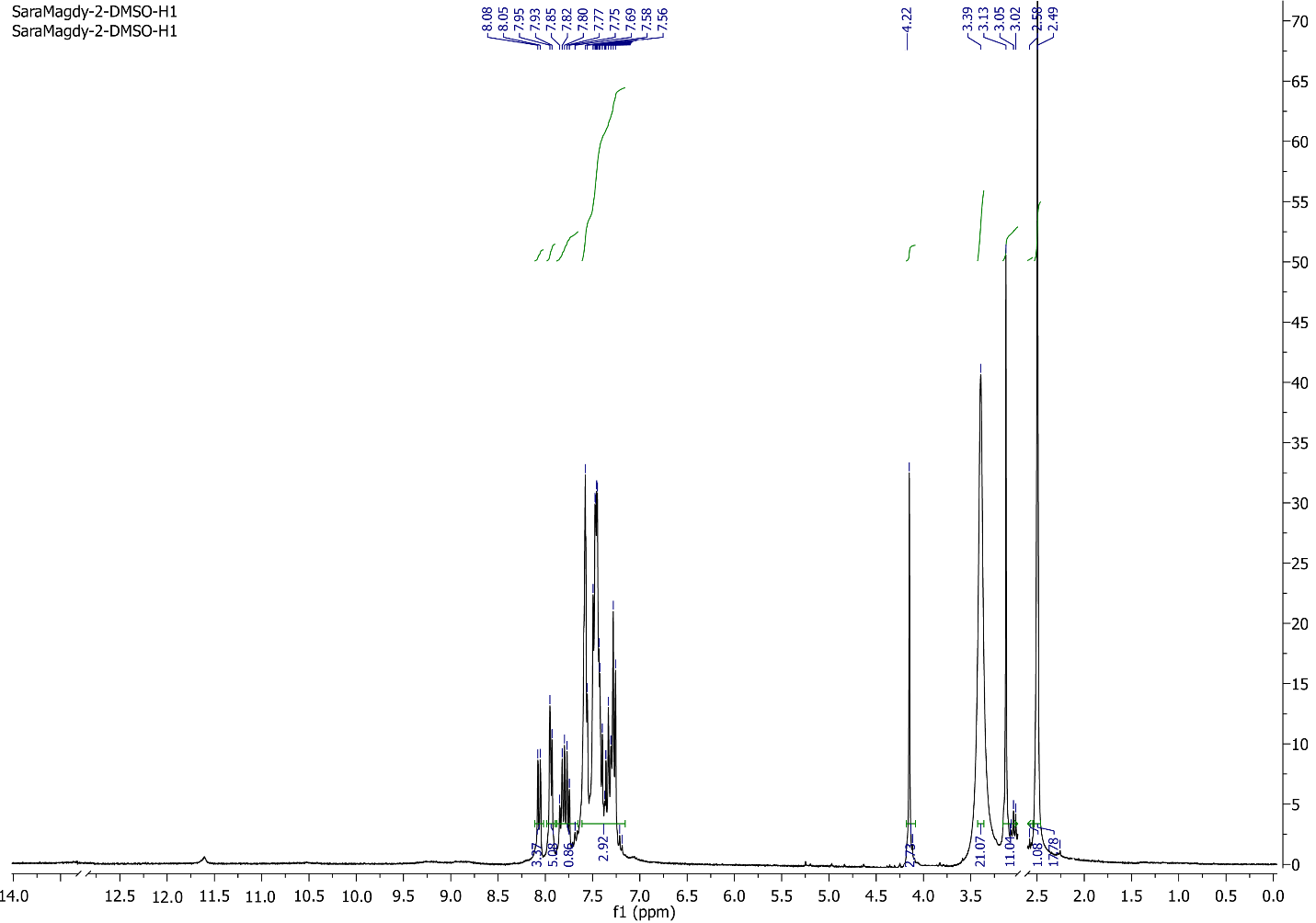


**Figure S2. ^1^H NMR spectrum (400 MHz, DMSO) of compound 1**


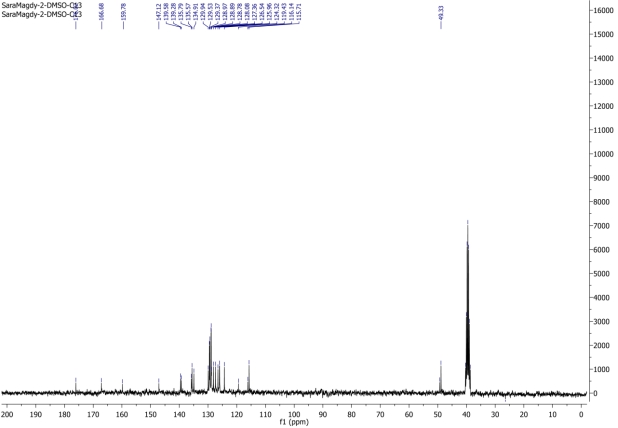


**Figure S3. ^13^C NMR spectrum (100 MHz, DMSO) of compound 1**

**Characterization of Compound 2:-**

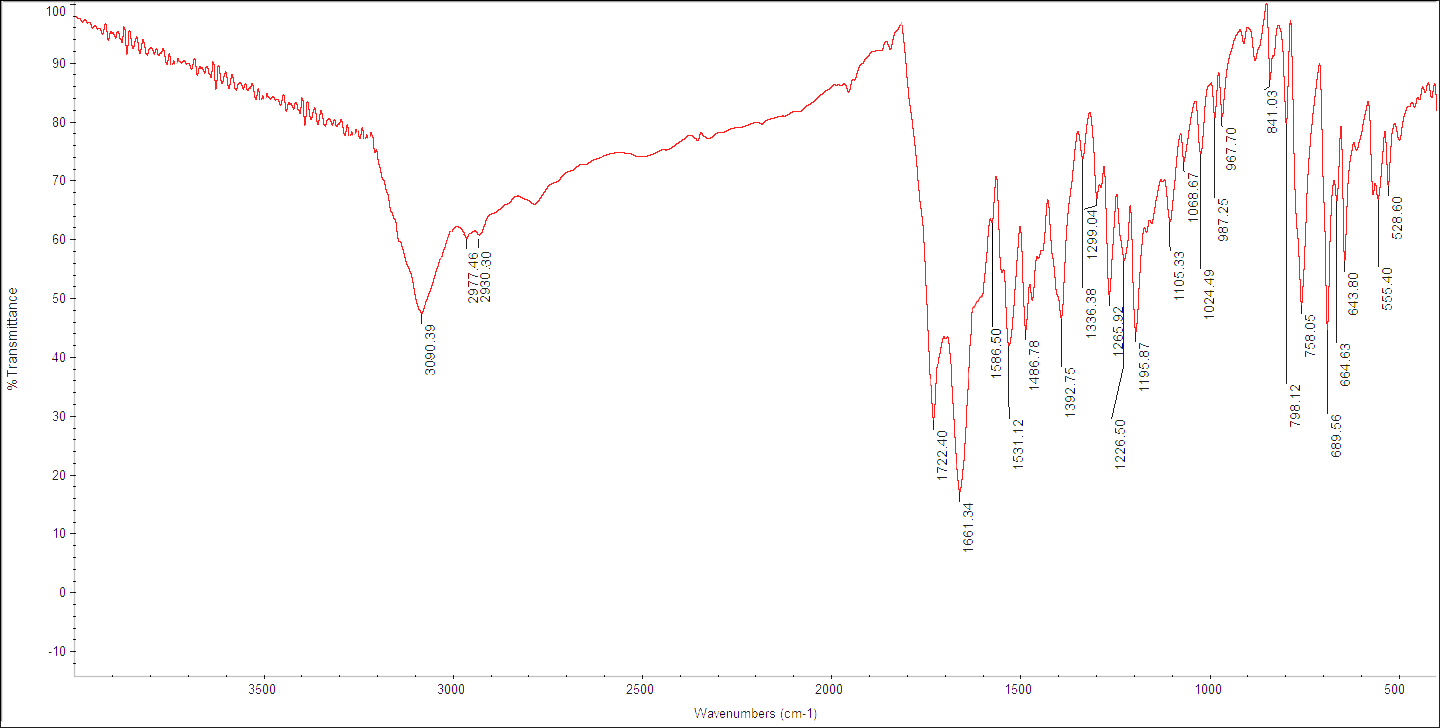


**Figure S4. IR spectrum of Compound 2**


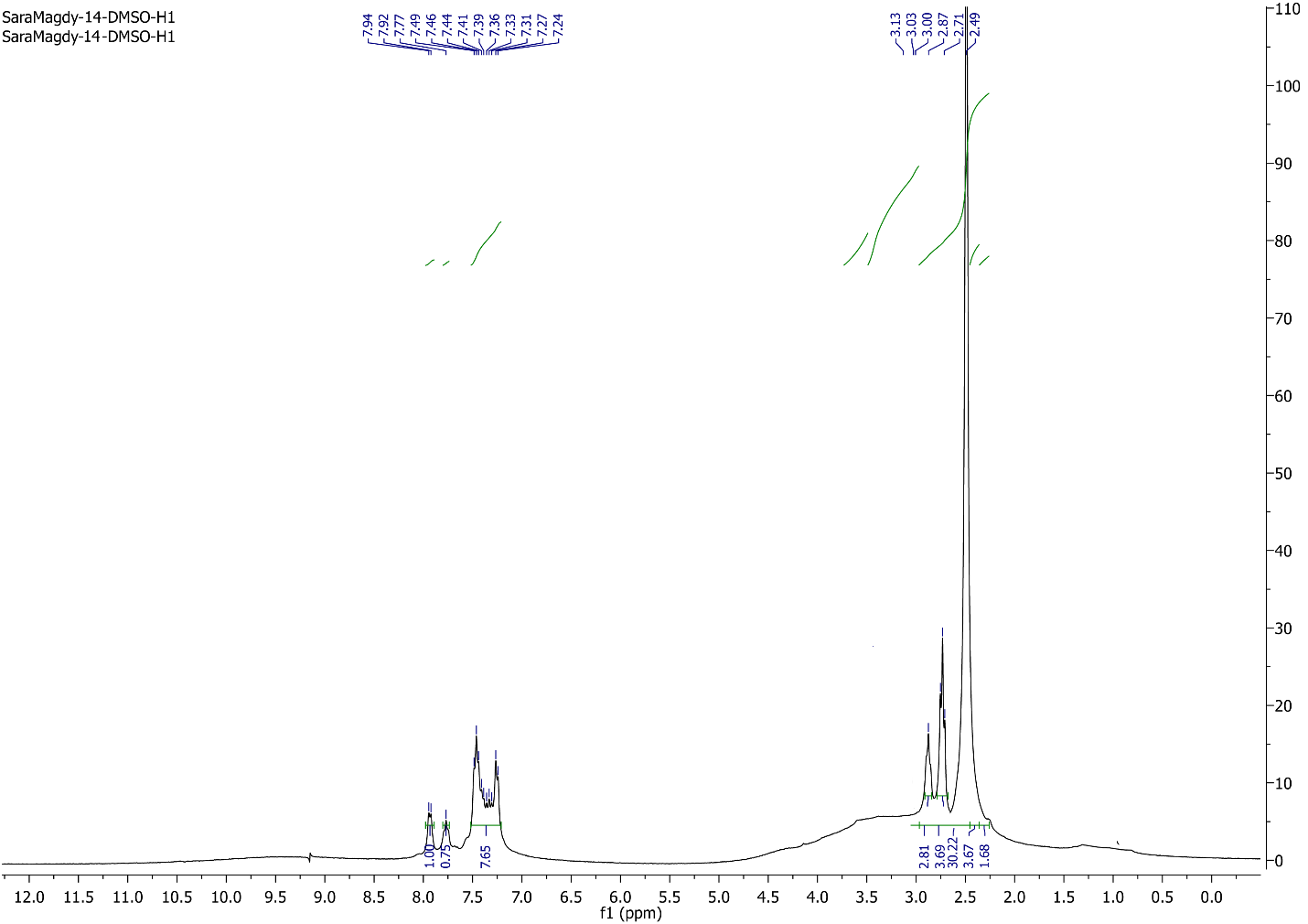


**Figure S5. ^1^H NMR spectrum (400 MHz, DMSO) of compound 2**

**
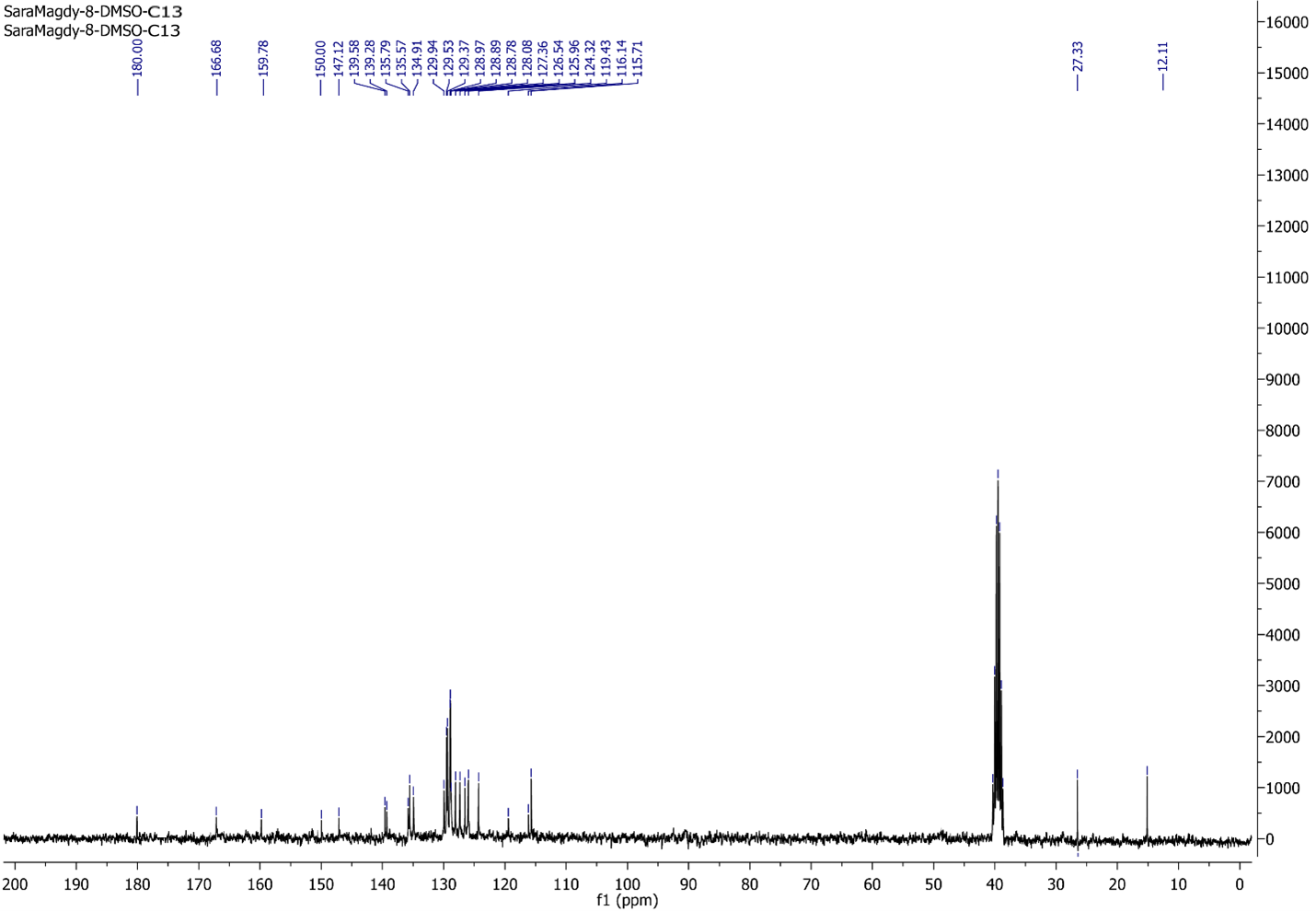
**

**Figure S6. ^13^C NMR spectrum (100 MHz, DMSO) of compound 2**

**Characterization of Compound 3a:-**

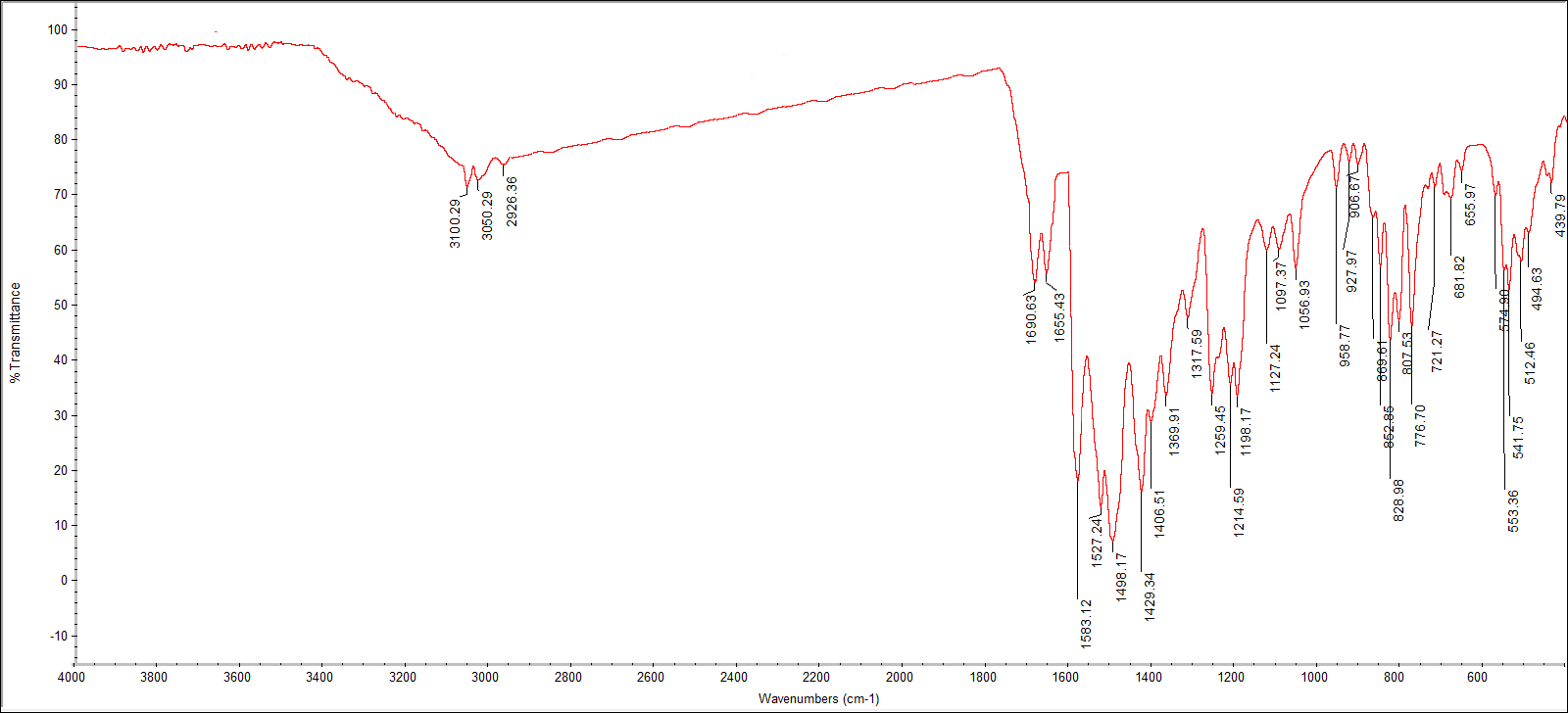


**Figure S7. IR spectrum of Compound 3a**


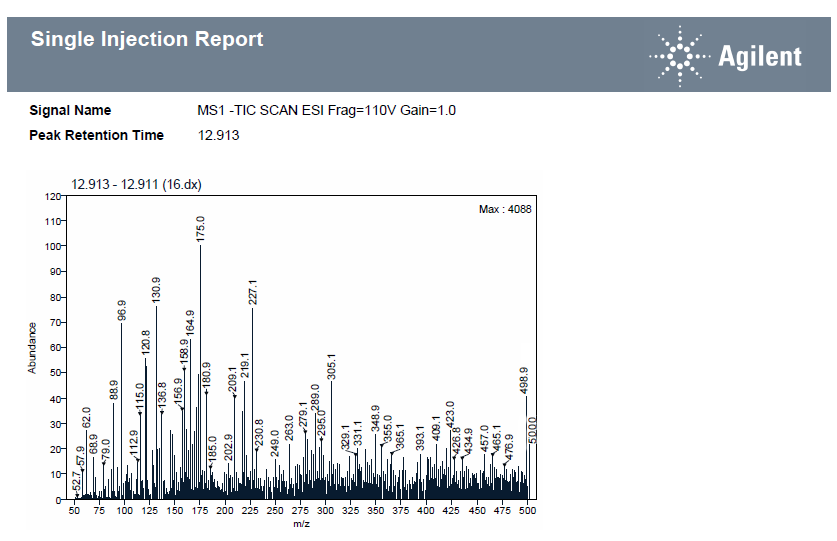


**Figure S8. Mass spectrum of Compound 3a**


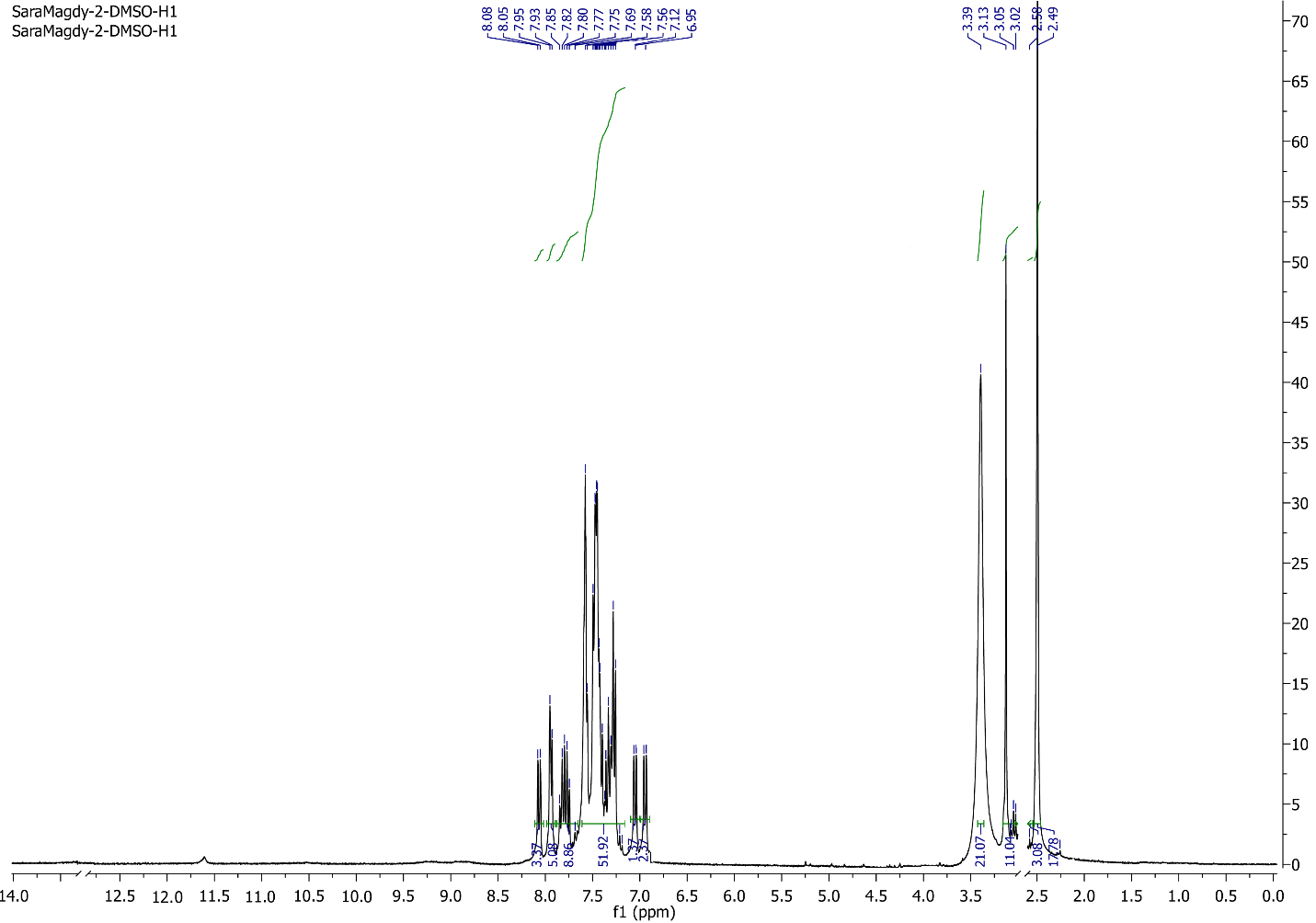


**Figure S9. ^1^H NMR spectrum (400 MHz, DMSO) of compound 3a**


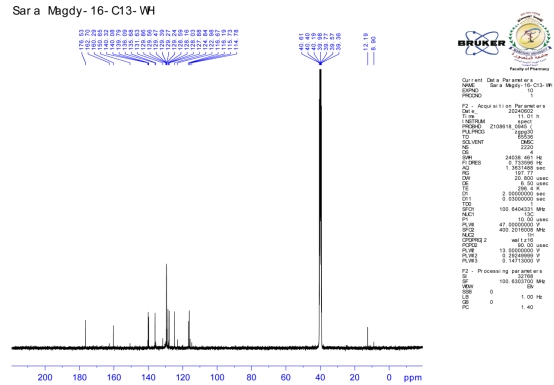


**Figure S10. ^13^C NMR spectrum (100 MHz, DMSO) of compound 3a**

**Characterization of Compound 3b:-**

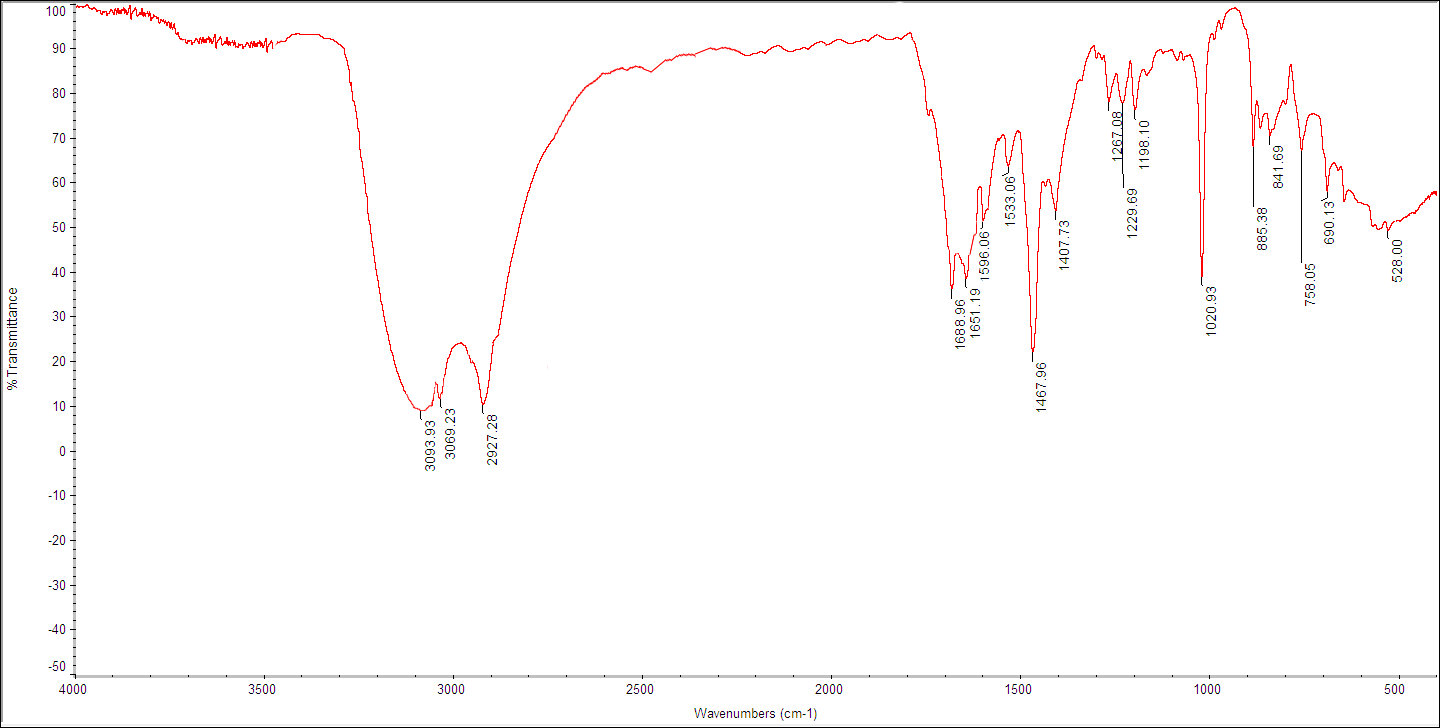


**Figure S11. IR spectrum of Compound 3b**


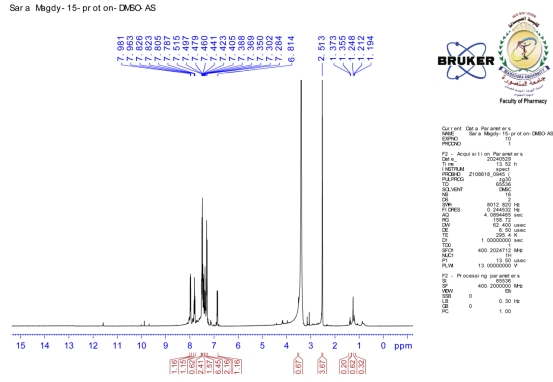


**Figure S12. ^1^ HNMR spectrum (400 MHz, DMSO) of compound 3b**


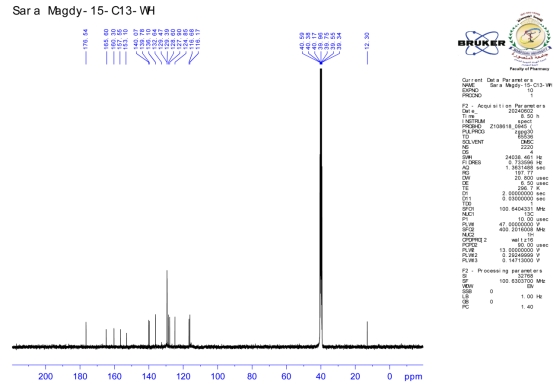


**Figure S13. ^13^C NMR spectrum (100 MHz, DMSO) of compound 3b**

**Characterization of Compound 3c:-**

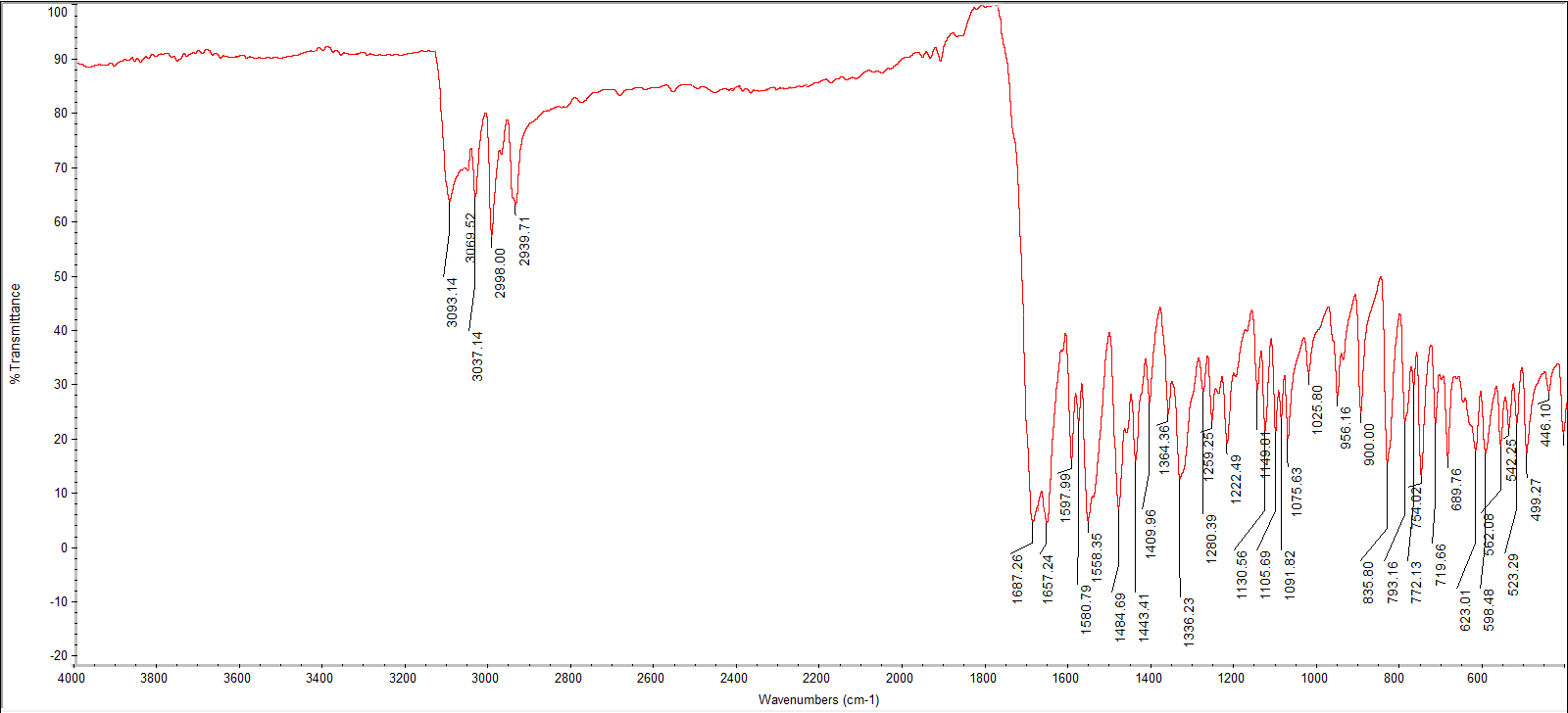


**Figure S14. IR spectrum of Compound 3c**


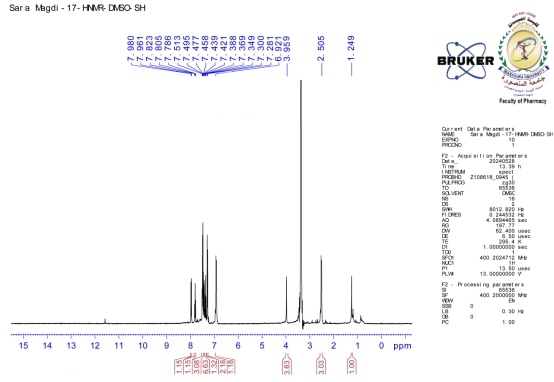


**Figure S15. ^1^ HNMR spectrum (400 MHz, DMSO) of compound 3c**


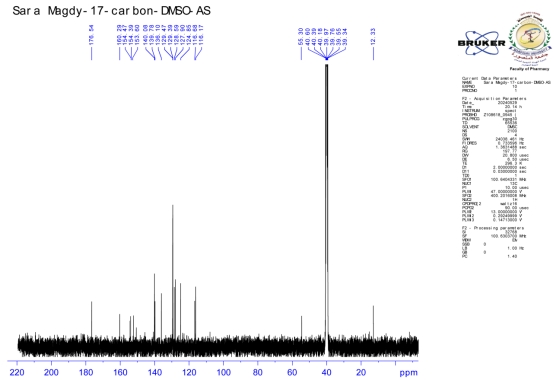


**Figure S16. ^13^C NMR spectrum (100 MHz, DMSO) of compound 3c**

**Characterization of Compound 3d:-**

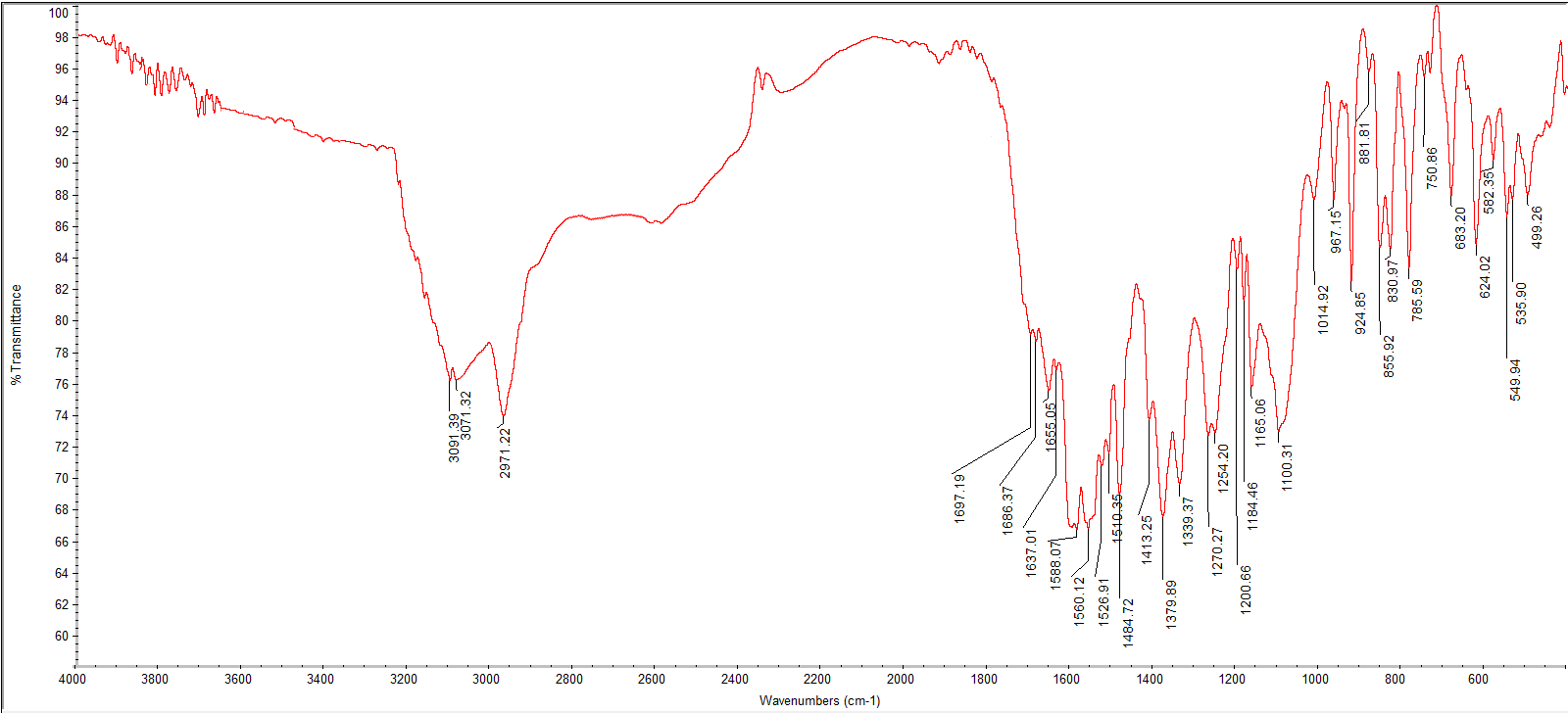


**Figure S17. IR spectrum of Compound 3d**


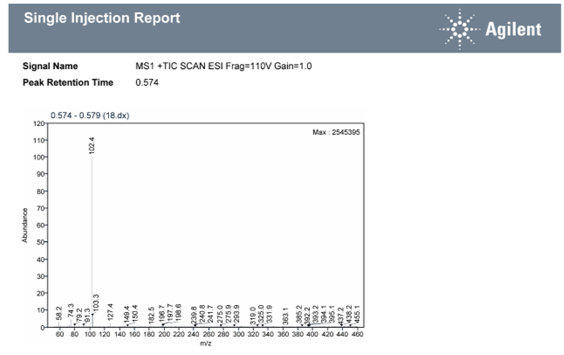


**Figure S18. Mass spectrum of Compound 3d**


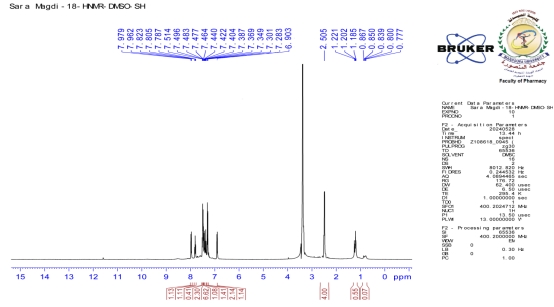


**Figure S19. ^1^ HNMR spectrum (400 MHz, DMSO) of compound 3d**


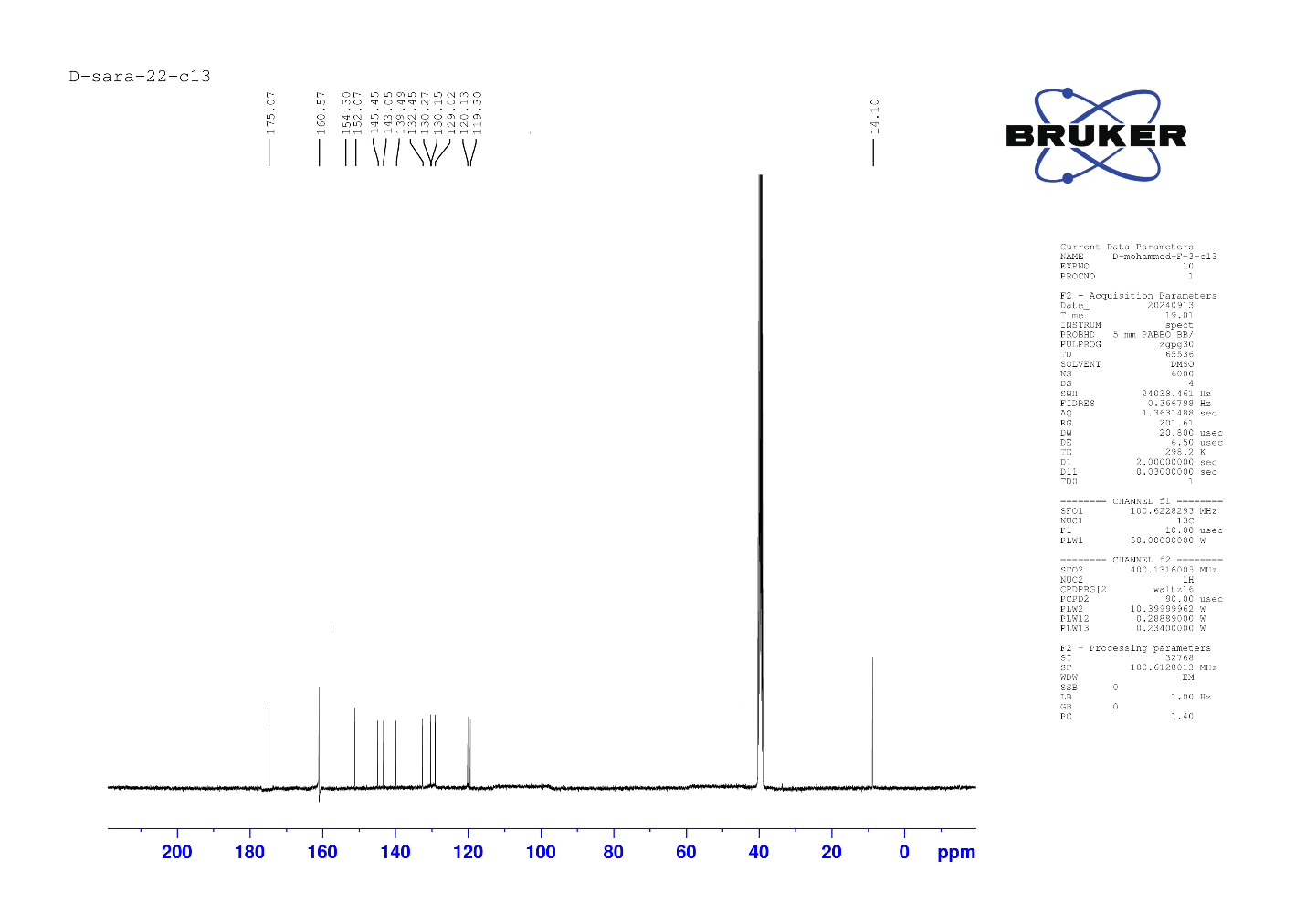


**Figure S20. ^13^C NMR spectrum (100 MHz, DMSO) of compound 3d**

**Characterization of Compound 4:-**

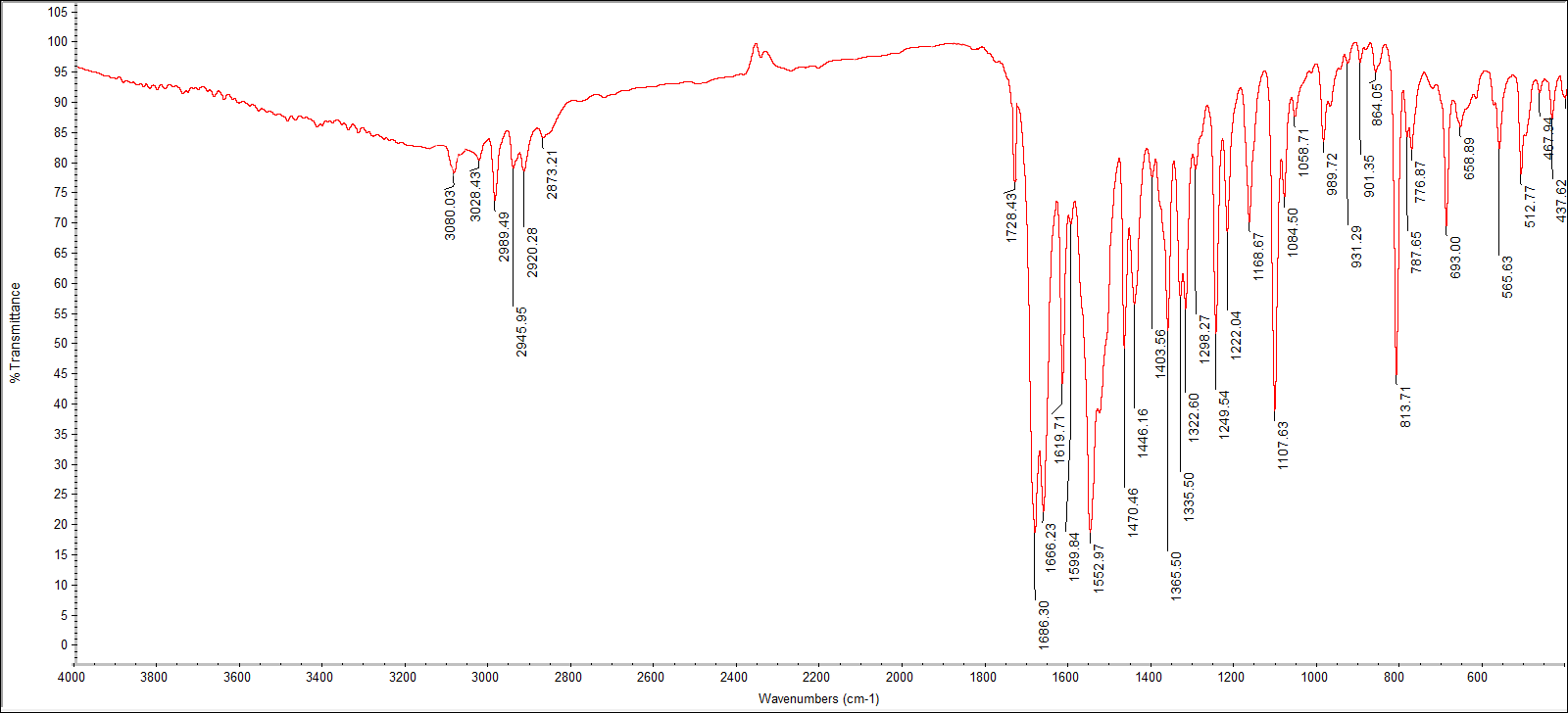


**Figure S21. IR spectrum of Compound 4**


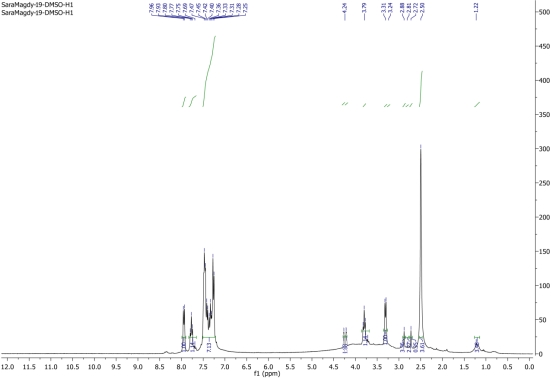


**Figure S22. ^1^HNMR spectrum (400 MHz, DMSO) of compound 4**


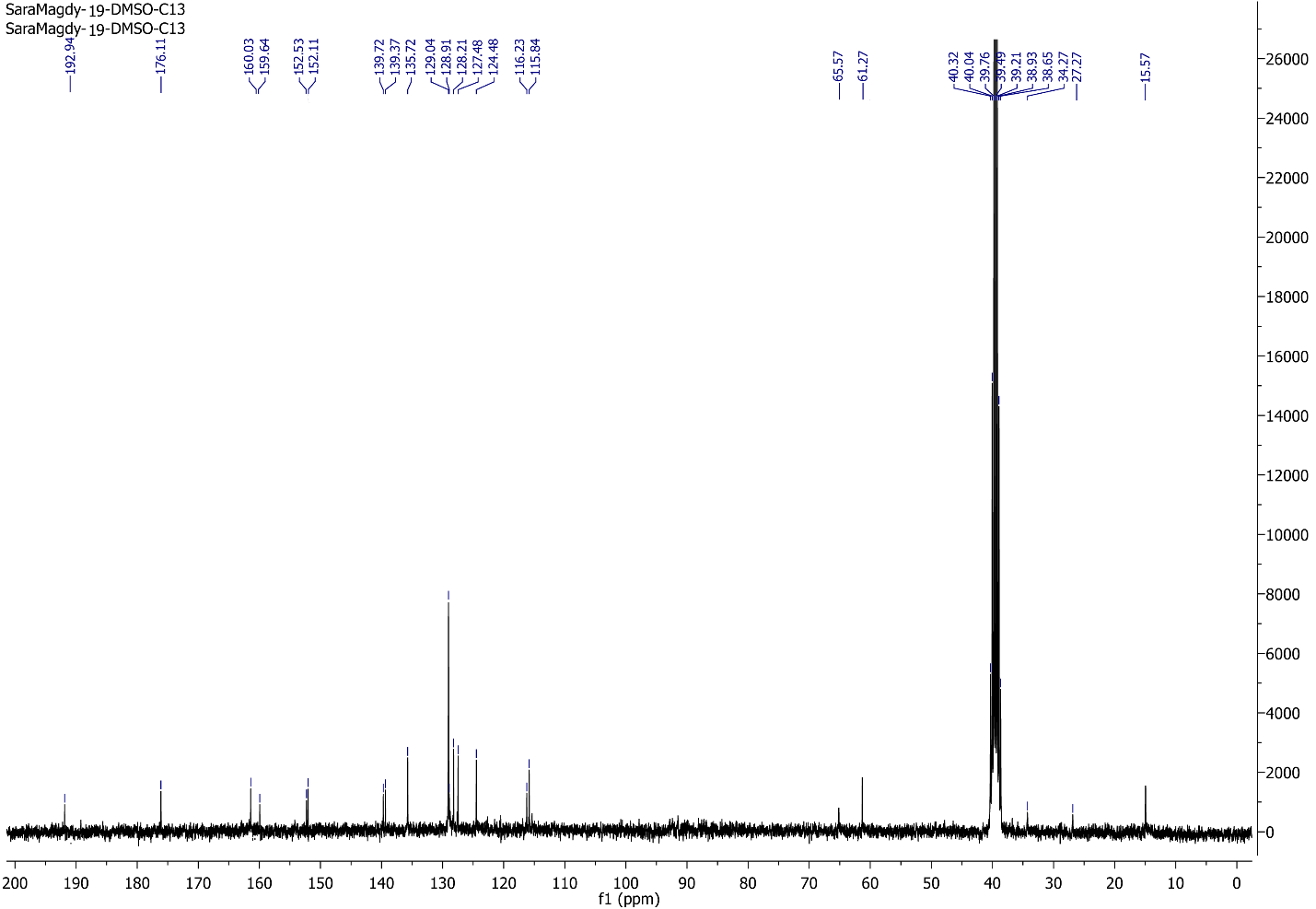


**Figure S23. ^13^C NMR spectrum (100 MHz, DMSO) of compound 4**

**Characterization of Compound 5:-**

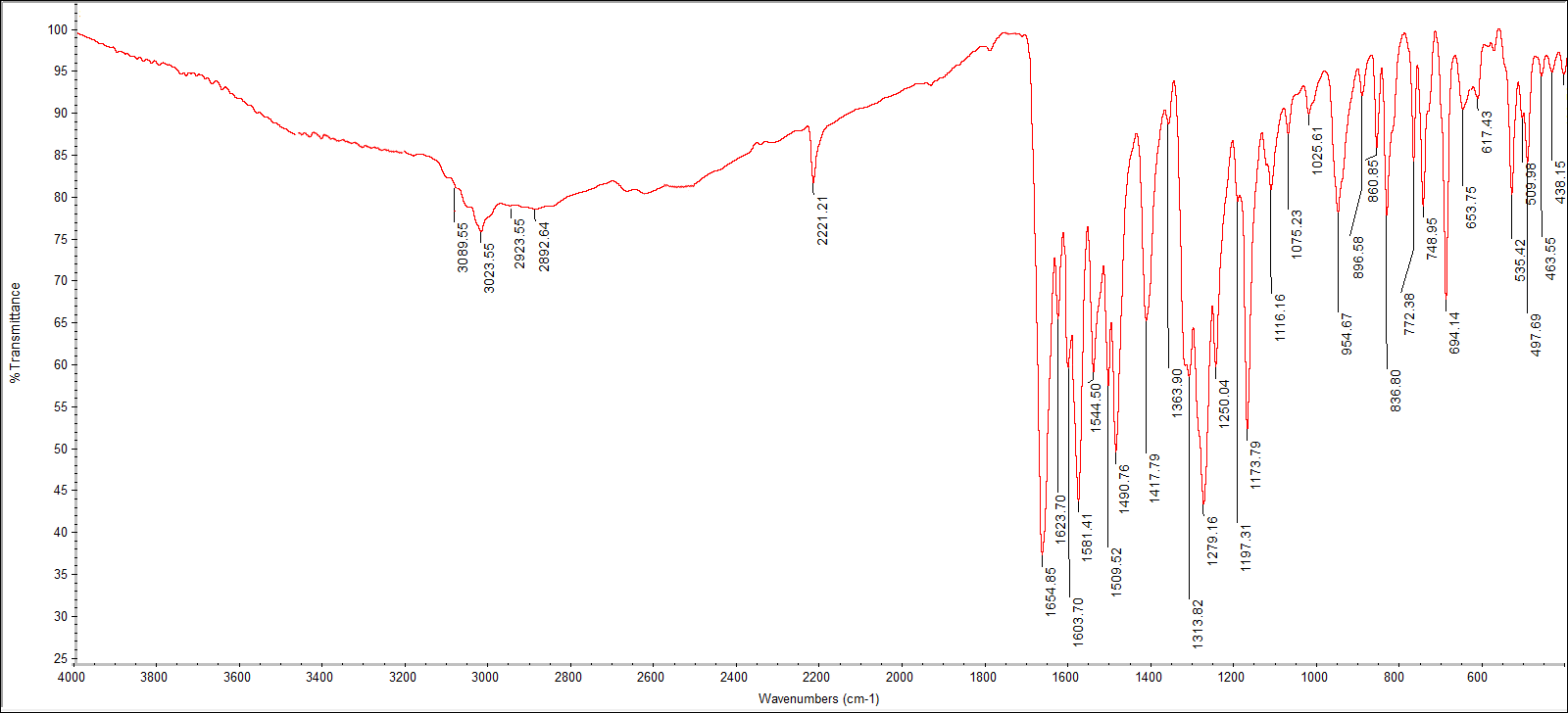


**Figure S24. IR spectrum of Compound 5**


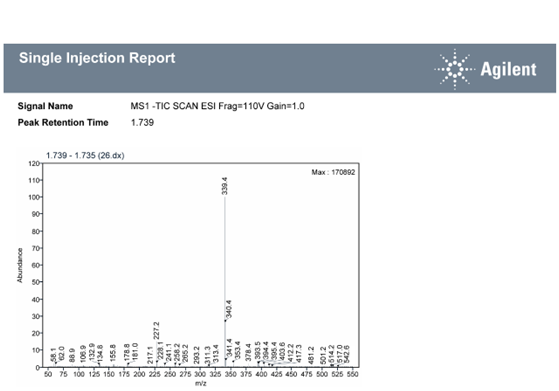


**Figure S25. Mass spectrum of Compound 6**


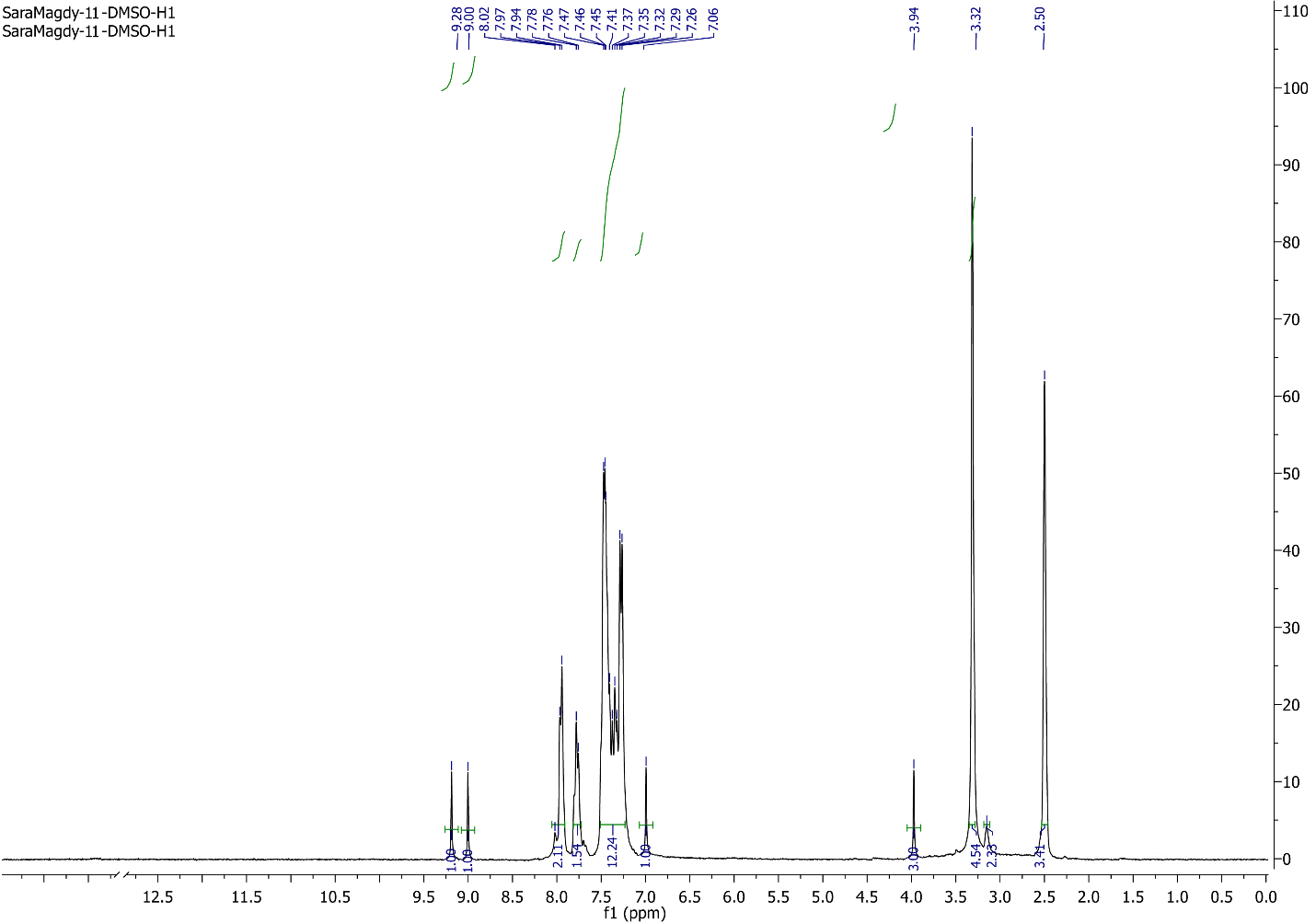


**Figure S26. ^1^ HNMR spectrum (400 MHz, DMSO) of compound 5**


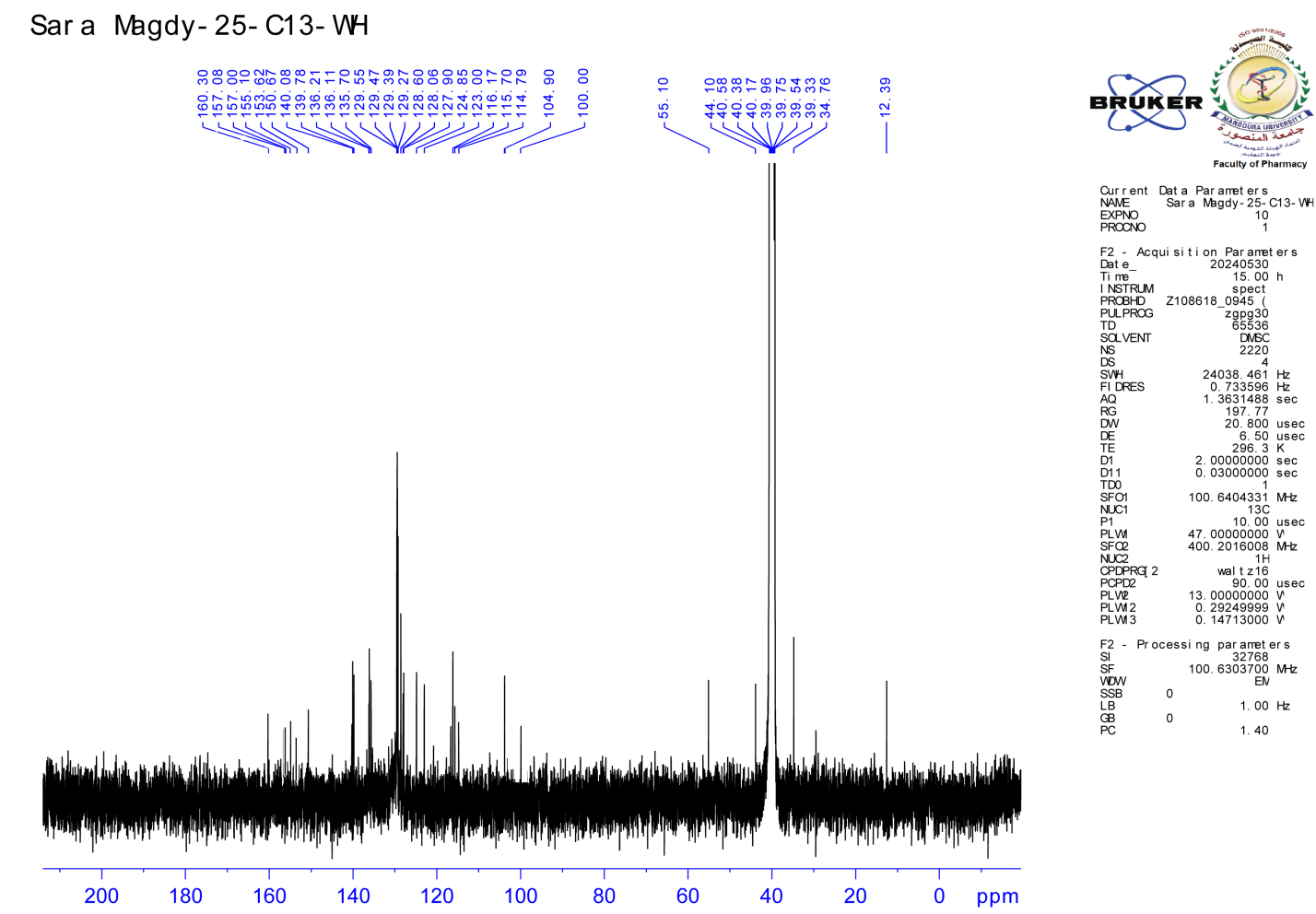


**Figure S27. ^13^C NMR spectrum (100 MHz, DMSO) of compound 5**

**Characterization of Compound 6a:-**

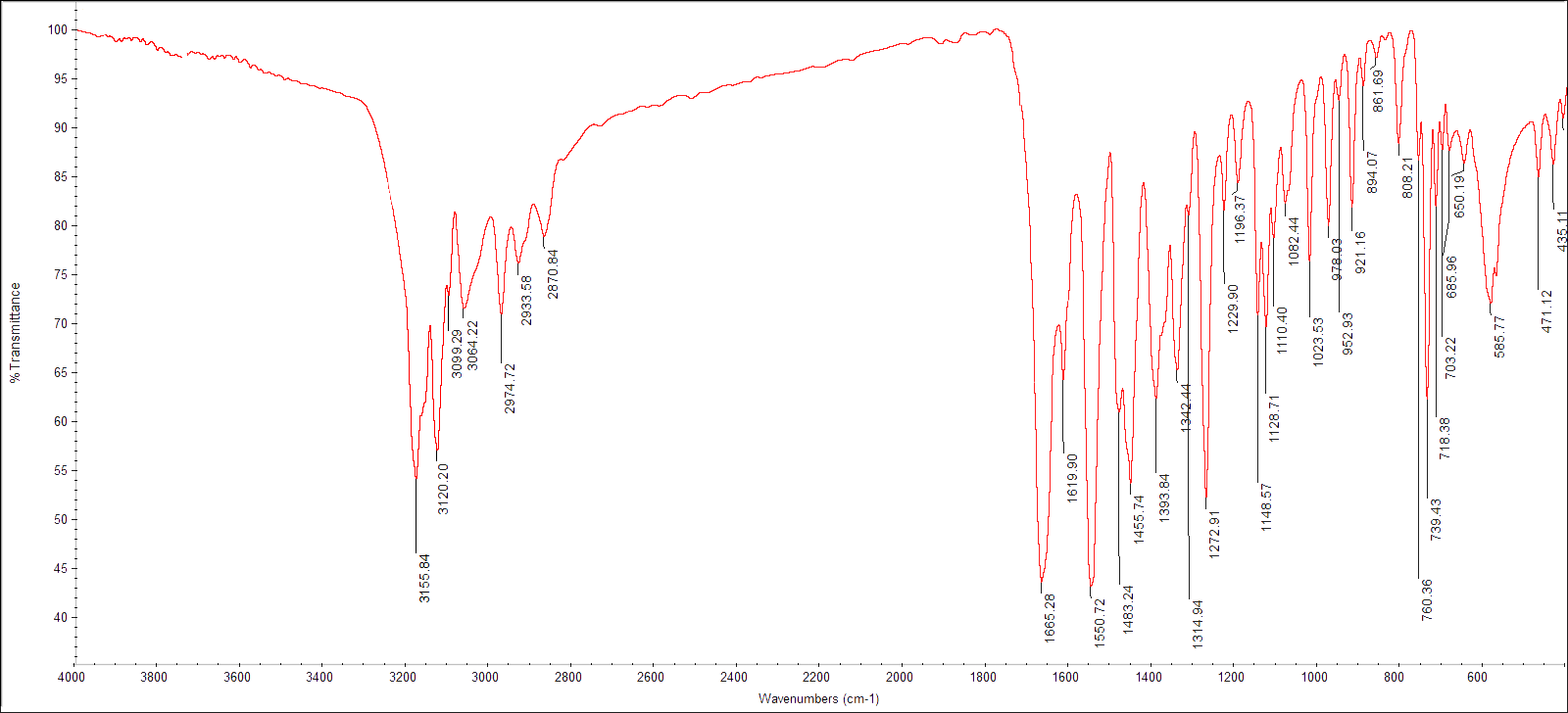


**Figure S28. IR spectrum of Compound 6a**


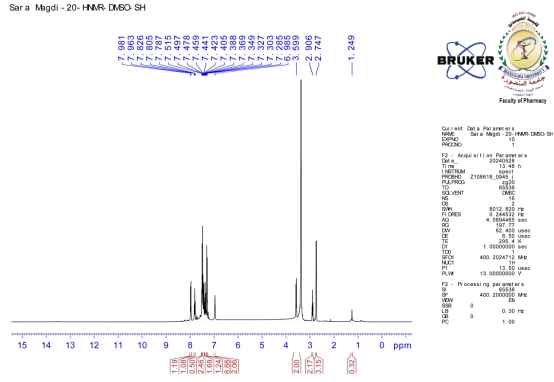


**Figure S29. ^1^ HNMR spectrum (400 MHz, DMSO) of compound 6a**


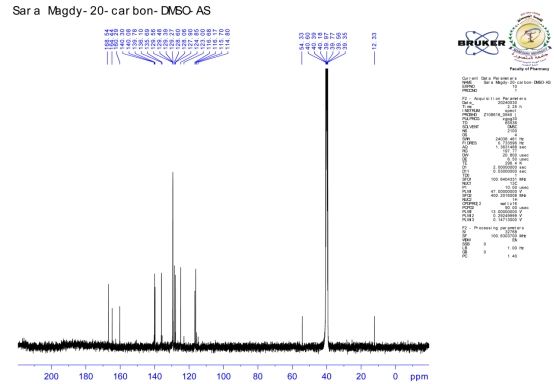


**Figure S30. ^13^C NMR spectrum (100 MHz, DMSO) of compound 6a**

**Characterization of Compound 6b:-**

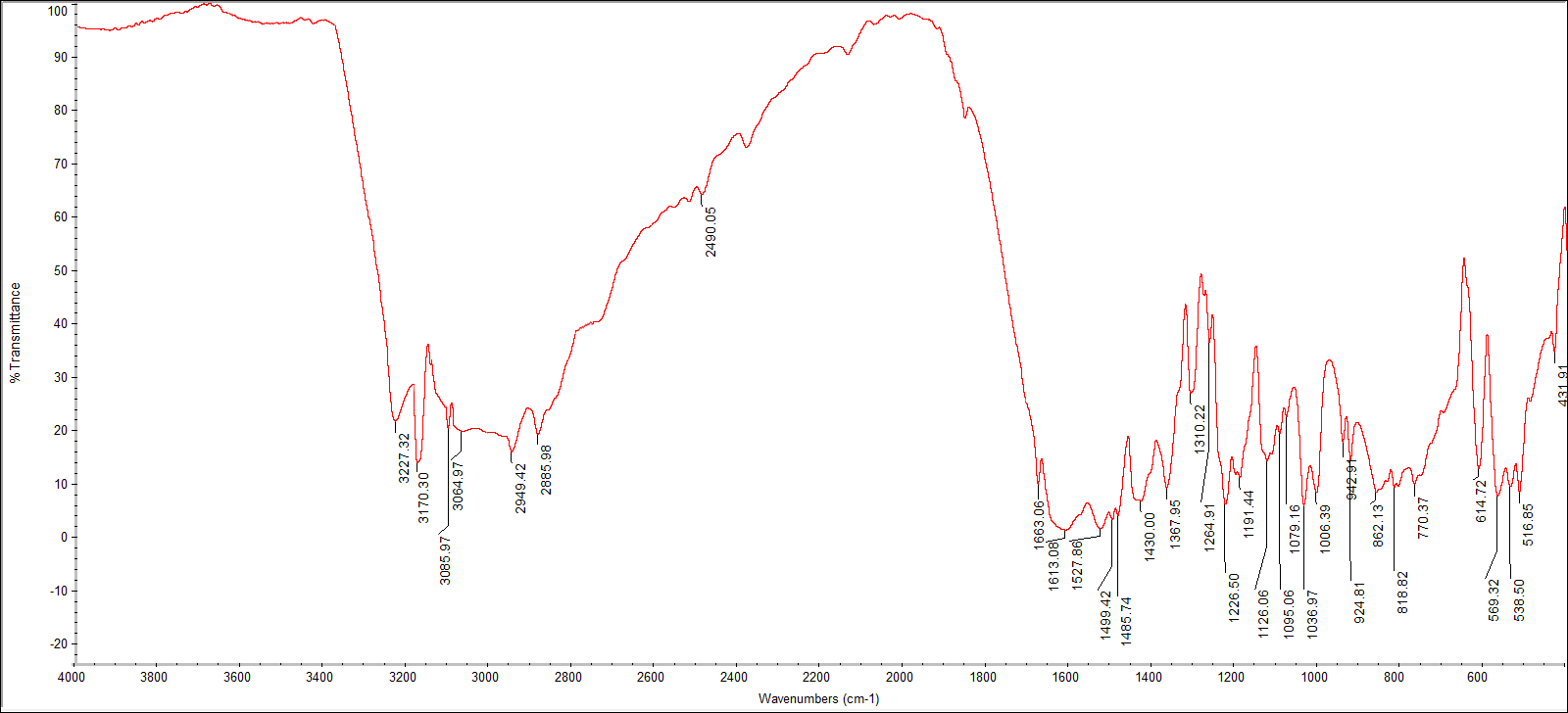


**Figure S31. IR spectrum of Compound 6b**


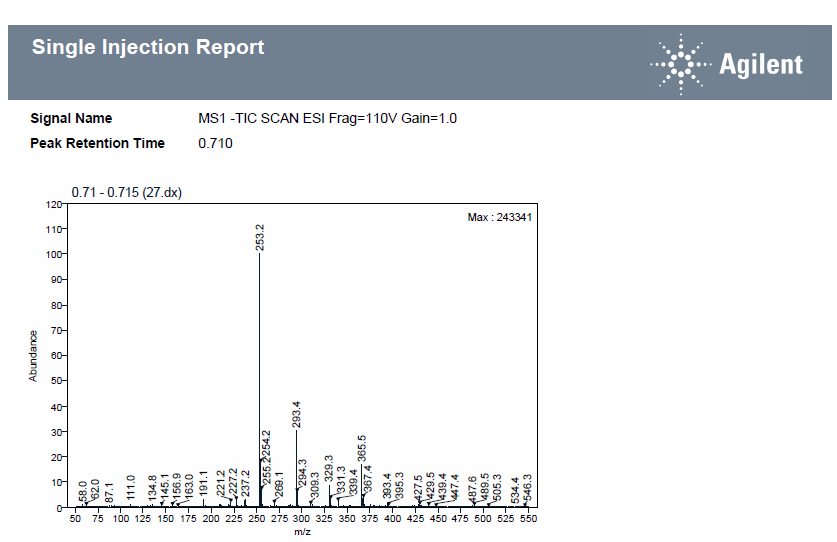


**Figure S32. Mass spectrum of Compound 6b**


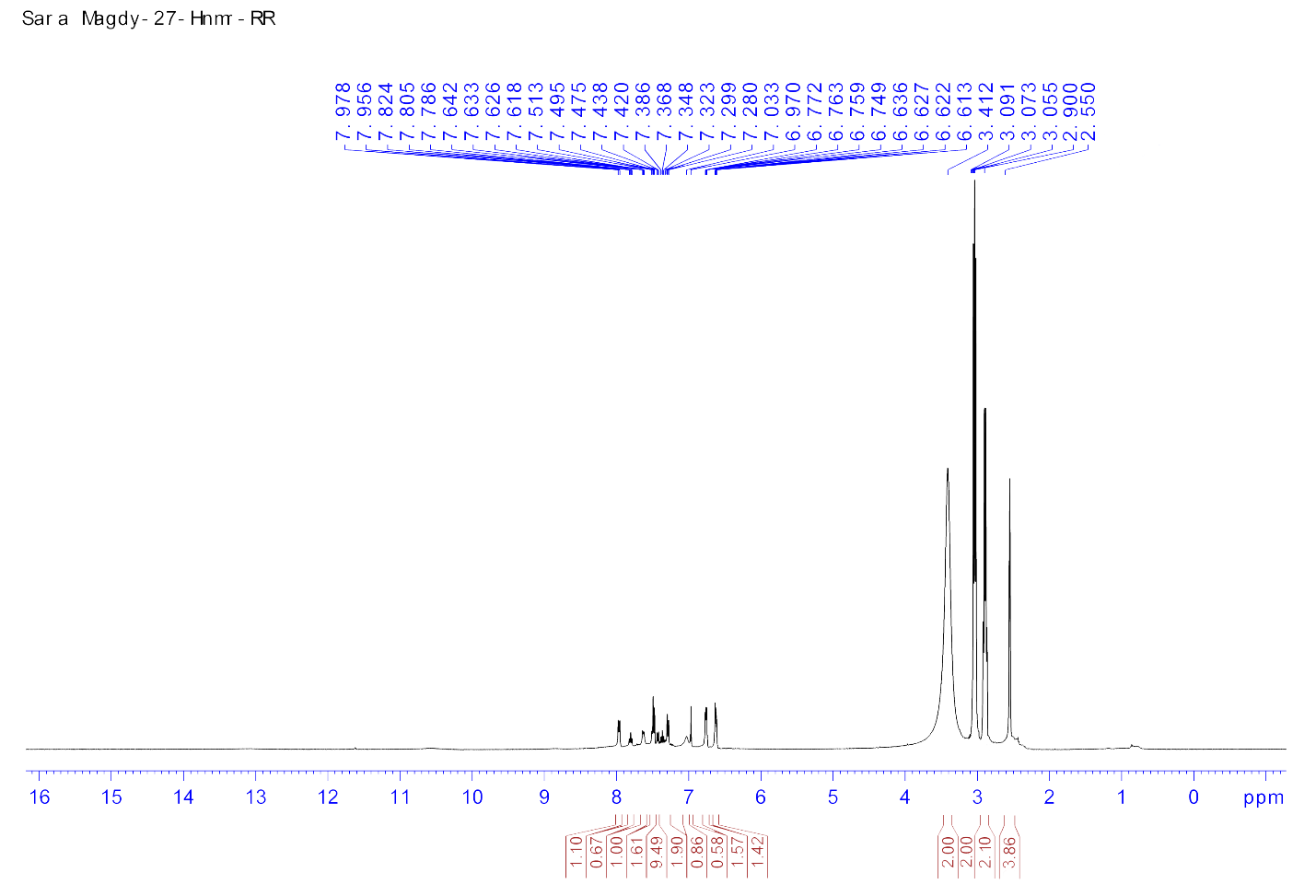


**Figure S33. ^1^ HNMR spectrum (400 MHz, DMSO) of compound 6b**

**Characterization of Compound 7:-**

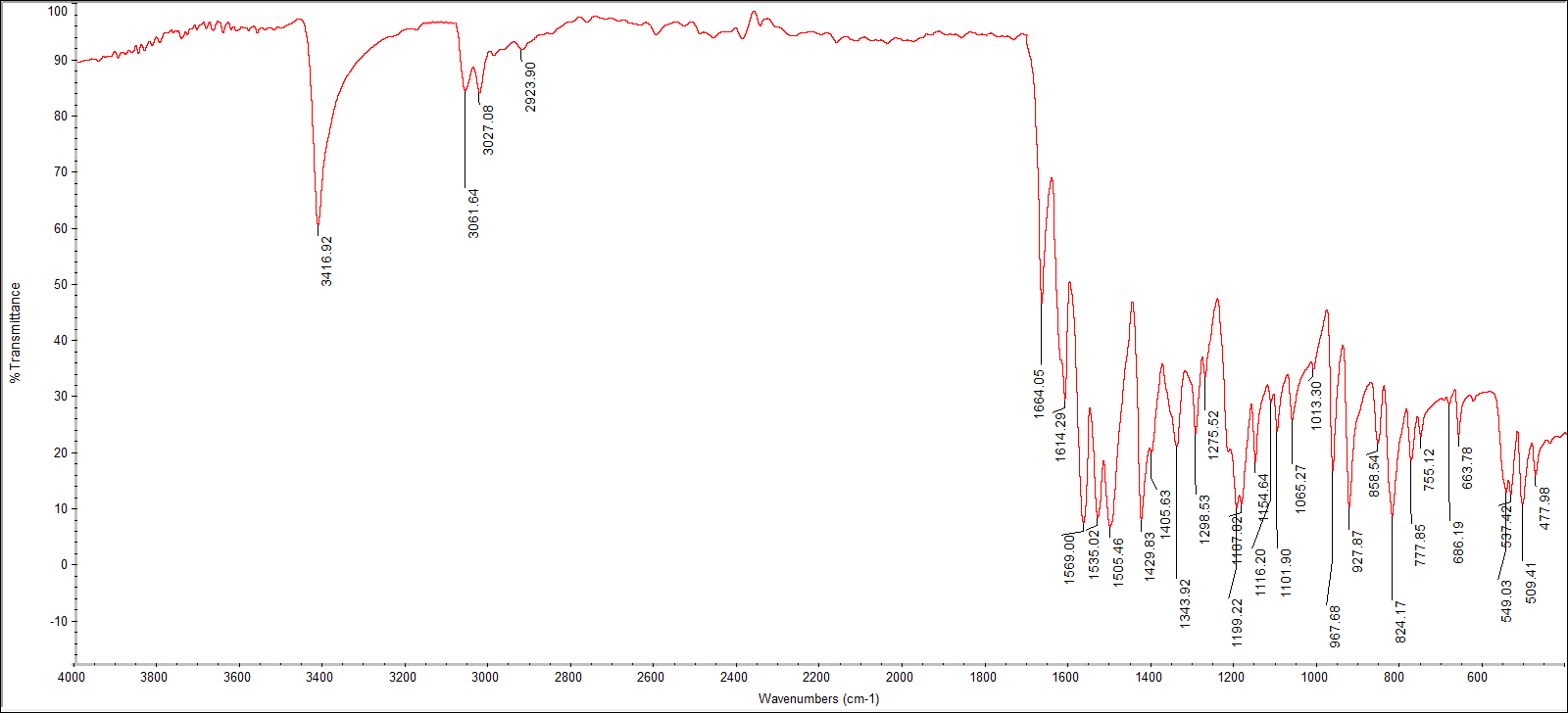


**Figure S34. IR spectrum of Compound 3b**


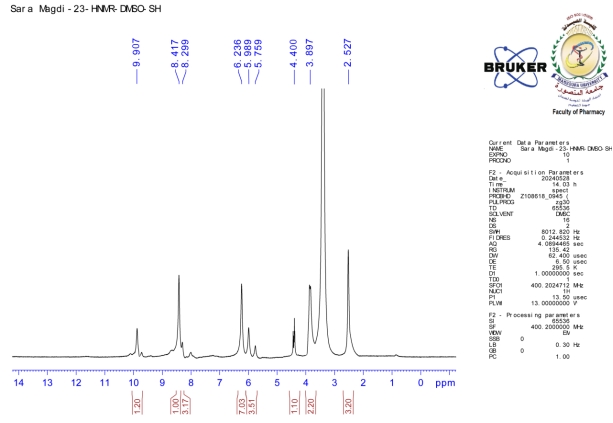


**Figure S35. ^1^ HNMR spectrum (400 MHz, DMSO) of compound 7**


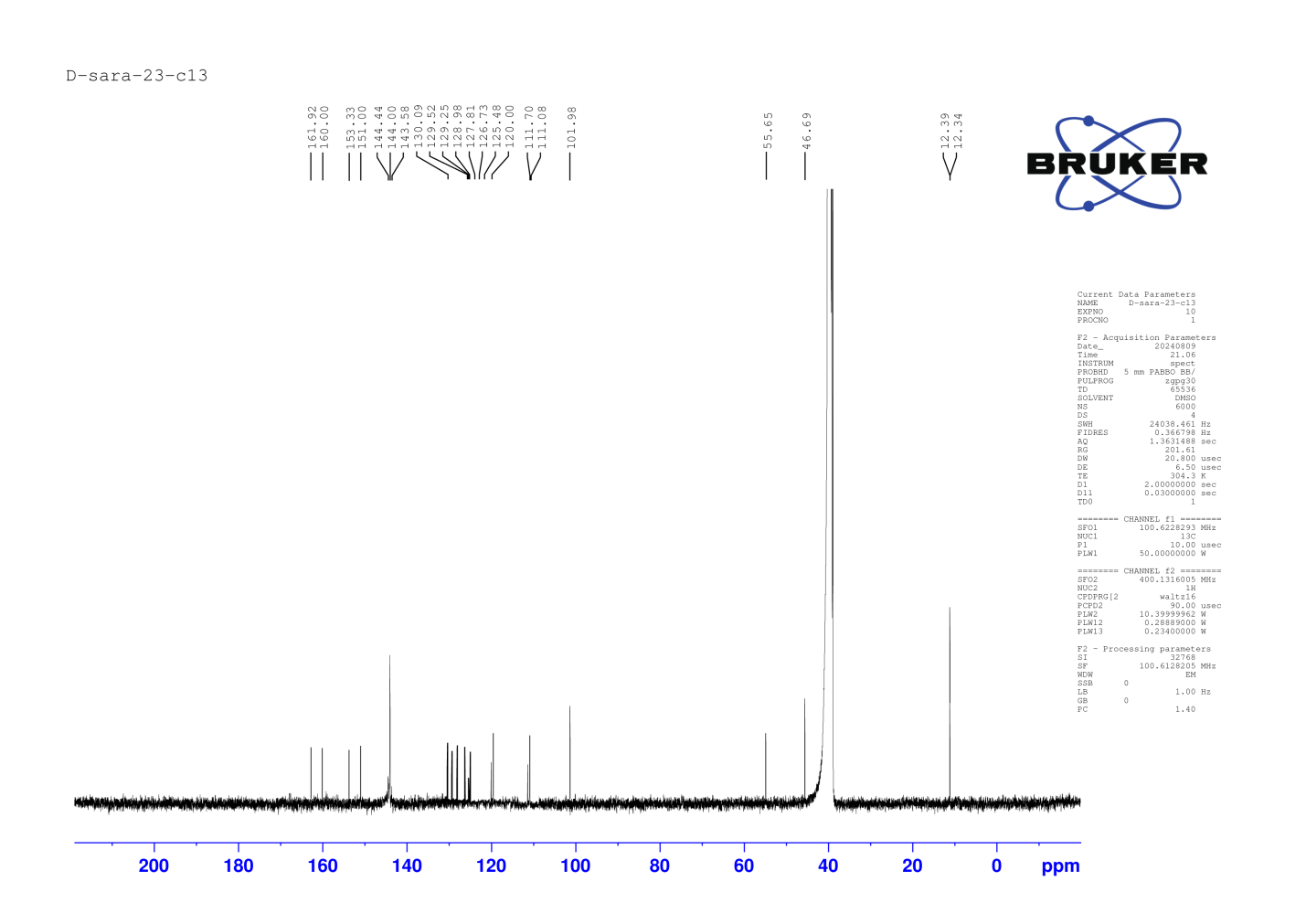


**Figure S36. ^13^C NMR spectrum (100 MHz, DMSO) of compound 7**

**Characterization of Compound 8a:-**

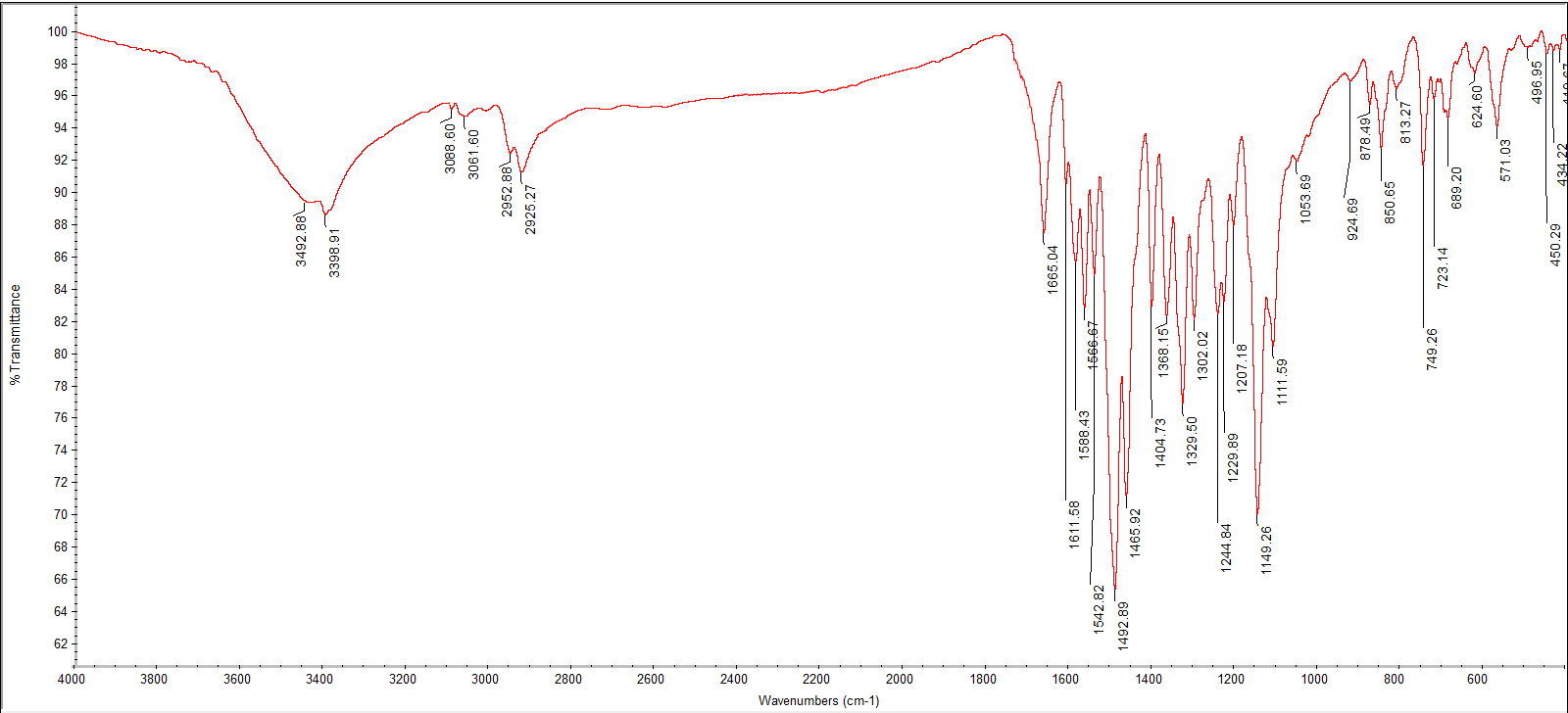


**Figure S37. IR spectrum of Compound 8a**


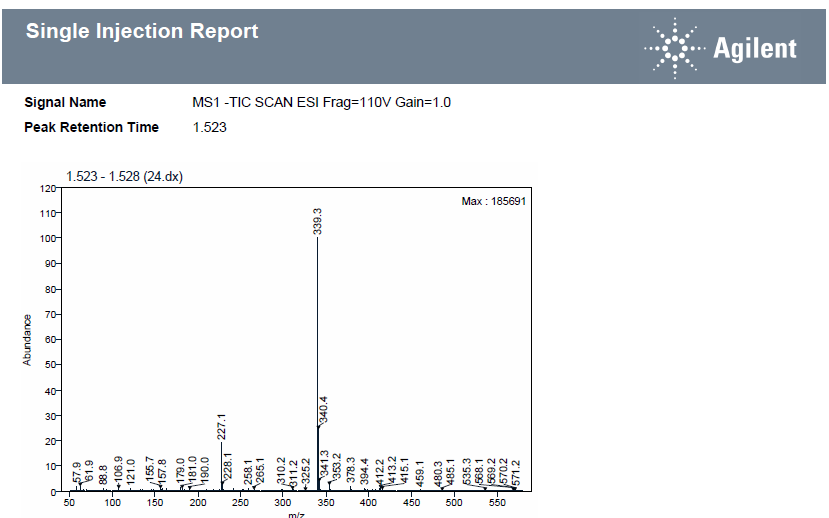


**Figure S38. Mass spectrum of compound 8a**


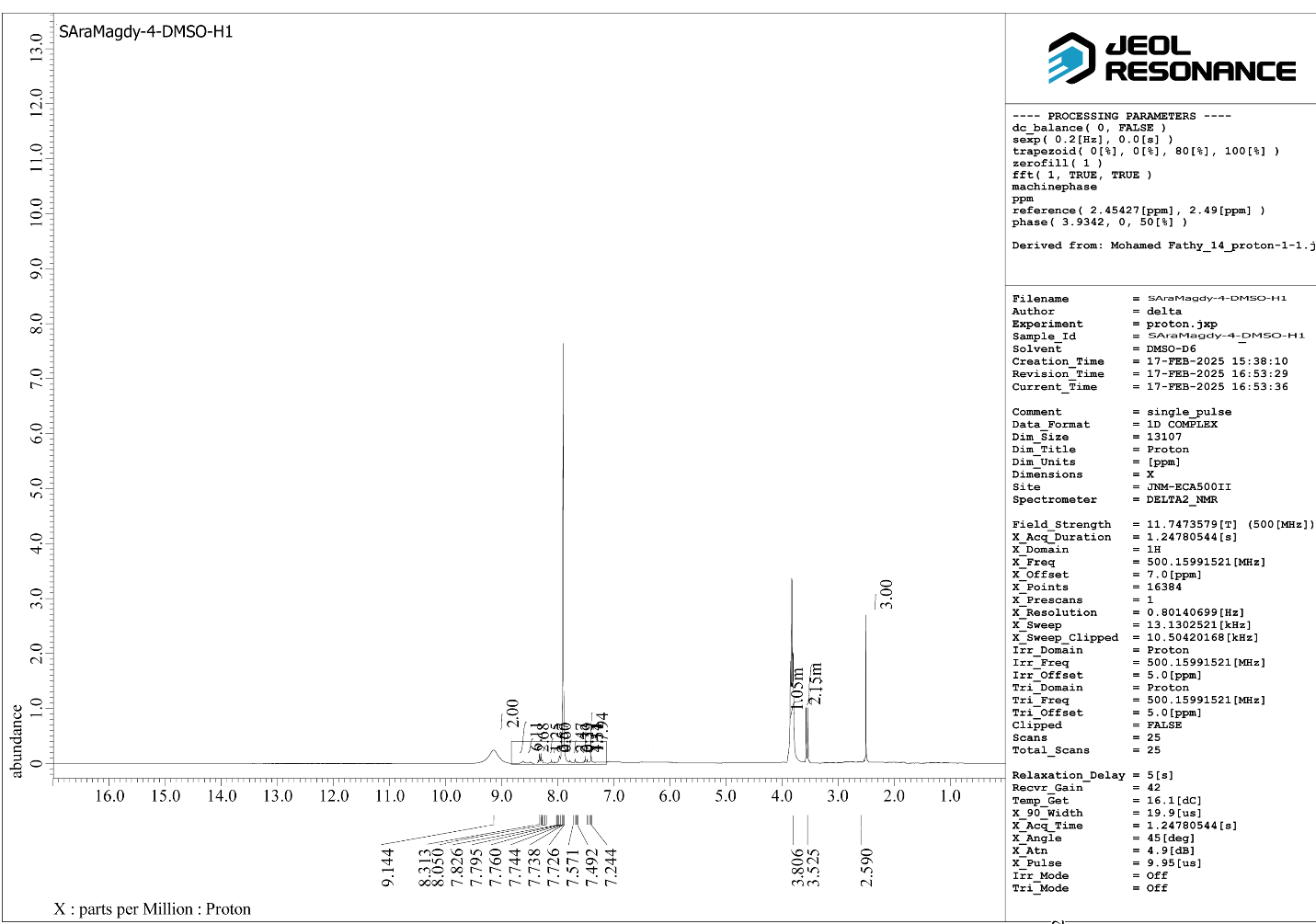


**Figure S39. ^1^ HNMR spectrum (400 MHz, DMSO) of compound 8a**


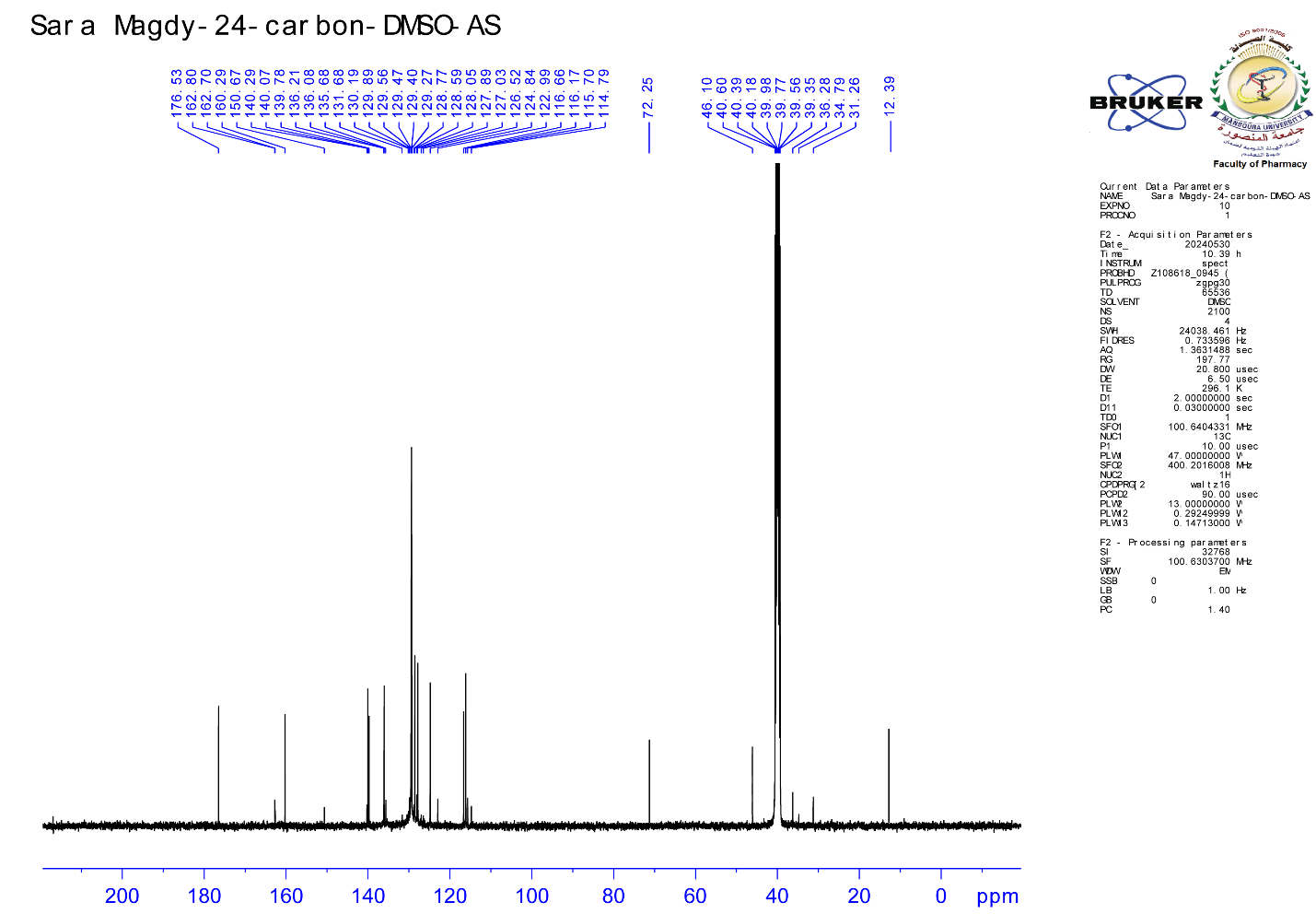


**Figure S40. ^13^C NMR spectrum (100 MHz, DMSO) of compound 8a**

**Characterization of Compound 8b:-**

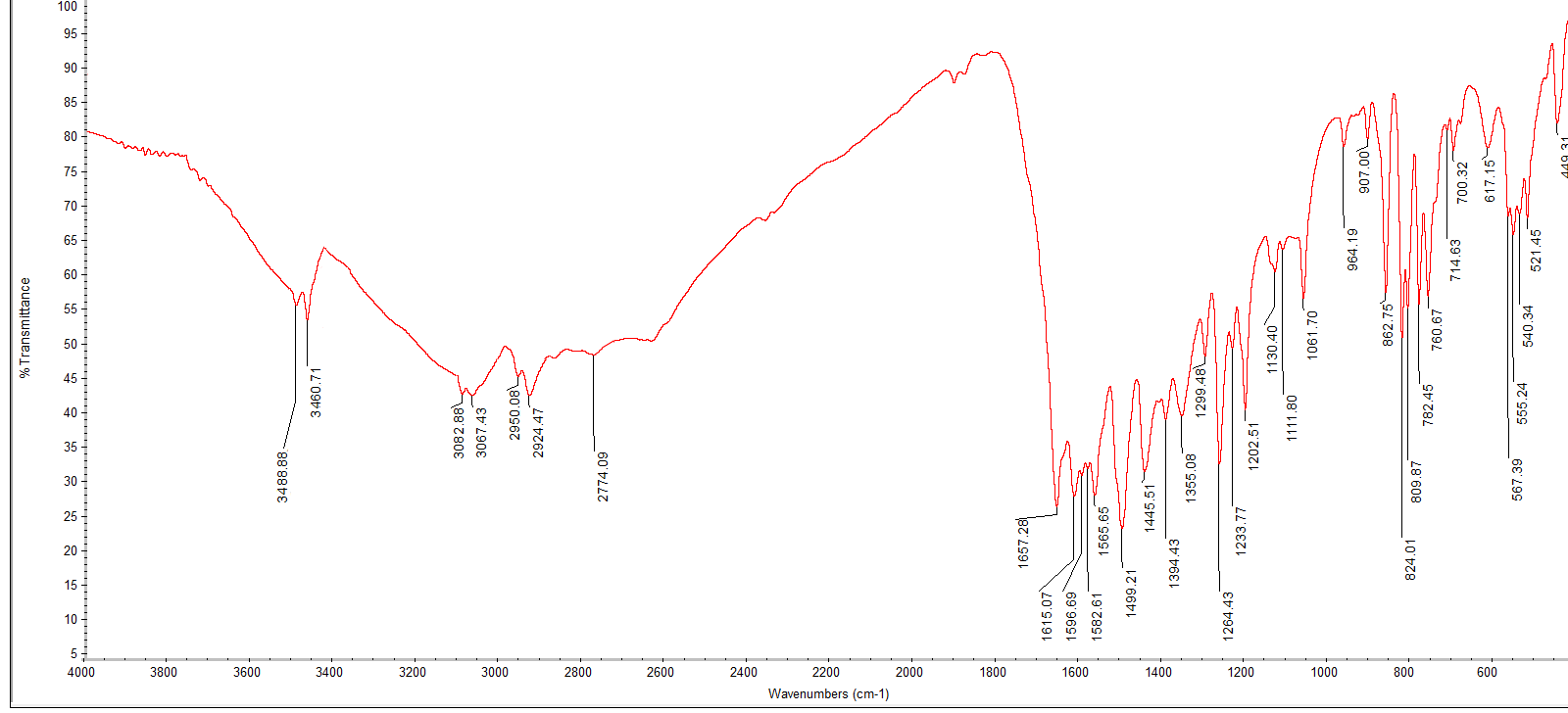


**Figure S41. IR spectrum of Compound 8b**


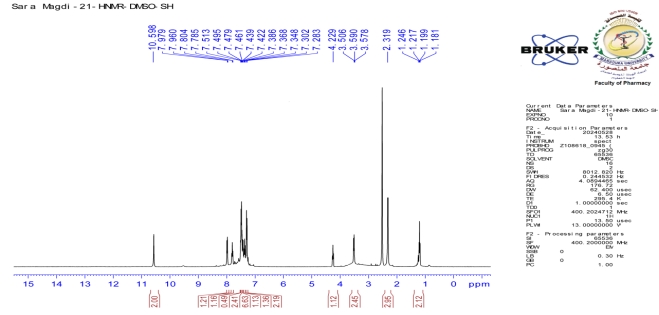


**Figure S42. ^1^HNMR spectrum (400 MHz, DMSO) of compound 8b**


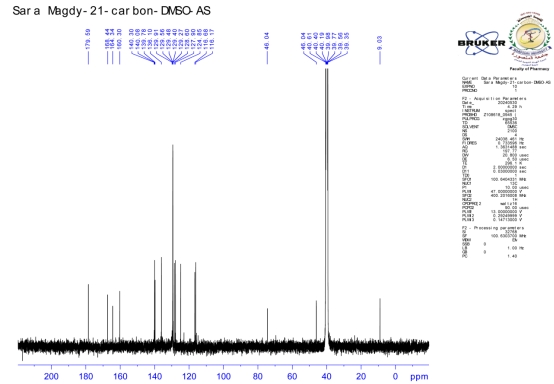


**Figure S43. ^13^C NMR spectrum (100 MHz, DMSO) of compound 8b**

**Characterization of Compound 9:-**

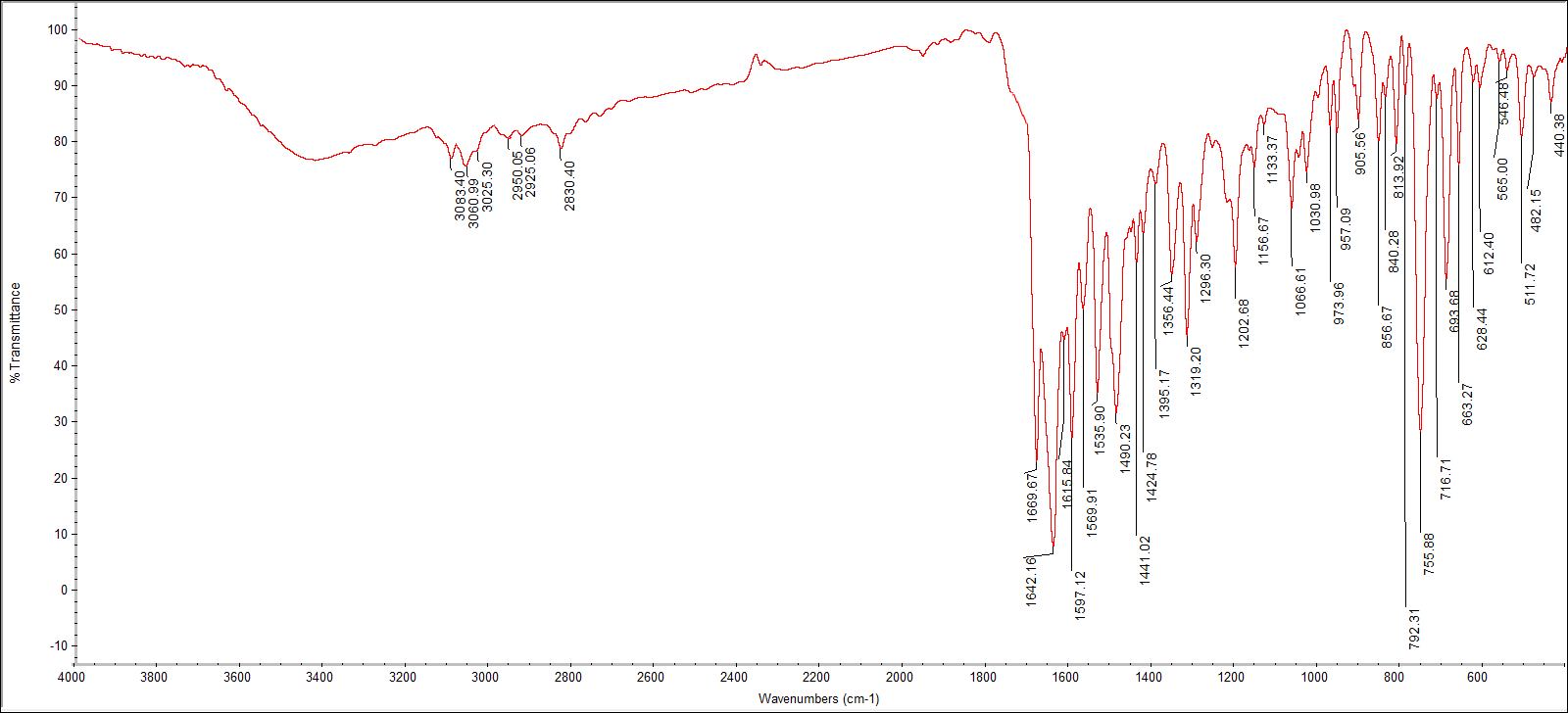


**Figure S44. IR spectrum of Compound 9**


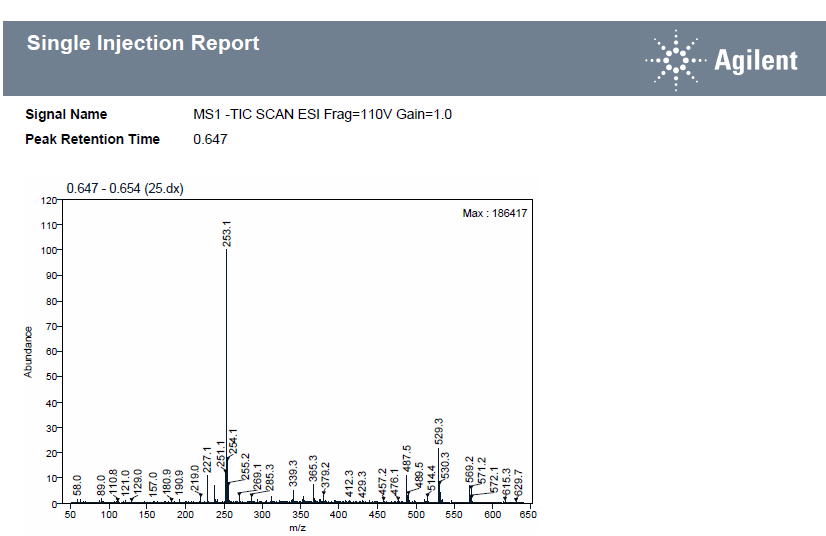


**Figure S45. Mass spectrum of Compound 9**


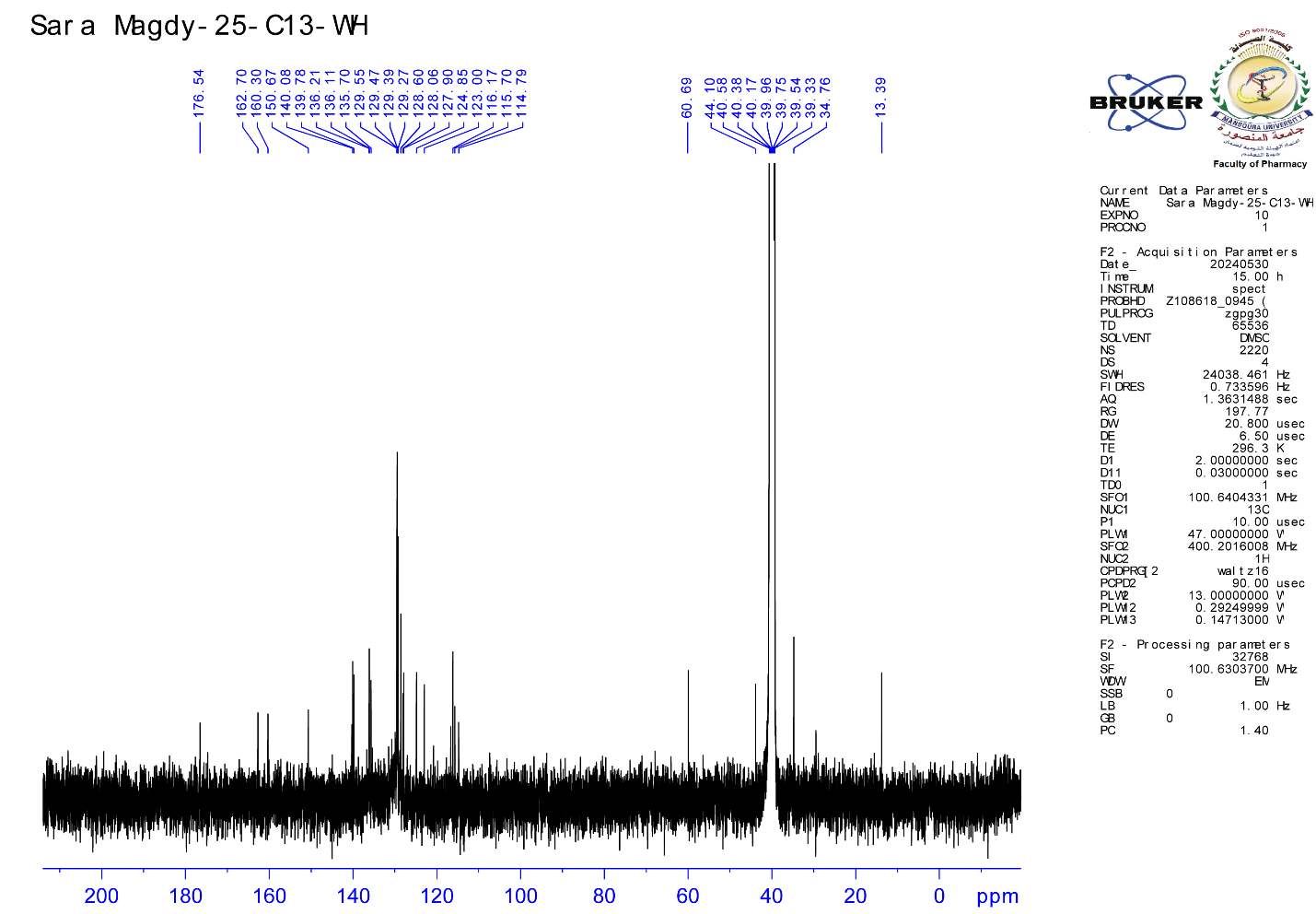


**Figure S46. ^13^C NMR spectrum (100 MHz, DMSO) of compound 9**

**Characterization of Compound 10:-**

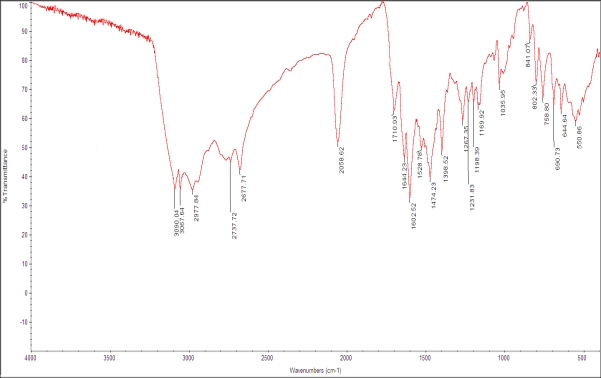


**Figure S47. IR spectrum of Compound 10**


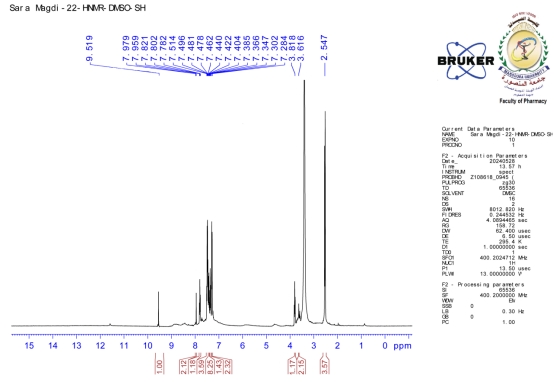


**Figure S48. ^1^ HNMR spectrum (400 MHz, DMSO) of compound 10**


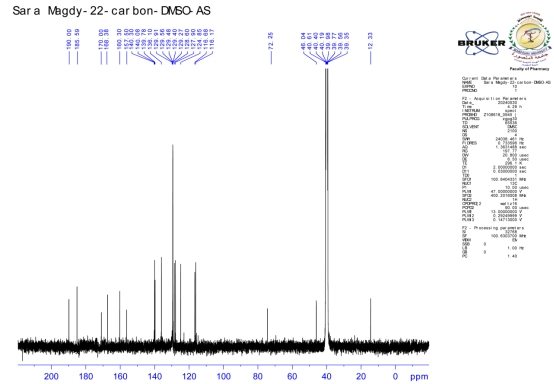


**Figure S49. ^13^C NMR spectrum (100 MHz, DMSO) of compound 10**

- 1. **Biological evaluation**
     1. ***In vitro* cytotoxic screening**

The cell lines were obtained from Karolinska Center, Department of Oncology and Pathology, Karolinska Institute and Hospital, Stockholm, Sweden, as follows: human liver HepG-2, breast MCF-7, and colorectal HCT-116 cancer cell lines and human diploid cell line WI-38. Exponentially, cells were placed in 10^4^ cells/ well for 24 h, and then fresh medium containing different concentrations of the tested sample. two-fold serial dilution of the tested sample was added using a multichannel pipette. Moreover, all cells were cultivated at 37 °C, 5% CO_2_ and 95% humidity. Also, incubation of control cells occurred at 37 °C. However, after incubation for 24 h different concentrations of the sample (100, 50, 25, 12.5, 6.25, 3.125, and 1.56 µM) were added, and continued the incubation for 48 h, then, add the crystal violet solution of 1% to each well for 0.5 h to examine viable cells. Rinse the wells using water until no stain. After that, add 30% glacial acetic acid to all wells with shaking plates on a Microplate reader (TECAN, Inc.) to measure the absorbance, using a test wavelength of 490 nm. Besides, compare the treated samples with the control cell. The cytotoxicity was estimated by IC_50_ in (μM), the concentration that inhibits 50% of growth of cancer cell.

- - 1. ***In vitro* inhibition assay of EGFR and VEGFR-2 activities**

EGFR assay: Abcam’s EGFR (ERBB) Human ELISA (Enzyme-Linked Immunosorbent Assay) kit is an in vitro enzyme-linked immunosorbent assay for the quantitative measurement of Human EGFR in serum, plasma and cell culture supernatants.

The master mixture (6 μL 5X Kinase Buffer + 1 μL ATP (500 μM) + 1 μL 50 X PTK substrate + 17 μL water) was prepared then, 25 μL to every well was added. 5 μL of Inhibitor solution of each well labeled as “Test Inhibitor” was added. However, for the “Positive Control" and “Blank”, 5 μL of the same solution without inhibitor (Inhibitor buffer) was added. 3 mL of 1X Kinase Buffer by mixing 600 μL of 5X Kinase Buffer with 2400 μL water was prepared. So, 3 mL of 1X Kinase Buffer became sufficient for 100 reactions. To the wells designated as "Blank", 20 μl of 1X Kinase Buffer was added. EGFR enzyme on ice was thawed. Upon first thaw, briefly the tube containing enzyme was spun to recover full content of the tube. The amount of EGFR required for the assay and dilute enzyme to 1 ng/μL with 1X Kinase Buffer was calculated. Moreover, the remaining undiluted enzyme in aliquots was stored at -80°C. The reaction was initiated by adding 20 μL of diluted EGFR enzyme to the wells designated “Positive Control” and "Test Inhibitor Control", after that it was incubated at 30°C for 40 minutes. After the 40 minutes reaction, 50 μL of Kinase-Glo Max reagent was added to each well and the plate was covered with aluminum foil and incubated at room temperature for 15 min. Luminescence was measured using the microplate reader. The concentration of the test compound causing 50% inhibition (IC_50_) was calculated from the concentration inhibition response curve and the data were compared with erlotinib as a standard EGFR inhibitor.

VEGFR-2 assay: the effect of the most promising cytotoxic compounds **1, 6b, 8a,** and **10** was evaluated for their *in vitro* inhibitory activity against Human VEGFR-2 using ELISA kit according to manufacturer's instructions (Ray Biotech). This assay employs an antibody specific for human VEGFR-2 coated on a 96-well plate. Samples and standards were added to the wells and incubated over night at 4 °C with gentle shaking. The wells were washed and biotinylated antibody was added followed by incubation for one hour at room temperature. After washing away unbound biotinylated antibody, HRP conjugated streptavidin was pipetted to the wells and

incubated at room temperature for 45 min. The wells were then washed; TMB substrate solution was added and incubated for 30 min. The color developed was in proportion to the amount of VEGFR-2 bound. The Stop Solution was added and the intensity of the color was measured at 450 nm. Percent inhibition was calculated by the comparison of compounds treated to control incubations. The concentration of the test compound causing 50% inhibition (IC_50_) was calculated from the concentration inhibition response curve and the data were compared with sorafenib as a standard VEGFR-2 inhibitor.

- - 1. **Cell cycle arrest and apoptosis of compound 6b**

Cell cycle analysis and apoptosis study were carried out using ab139418–Propidium Iodide Flow Cytometry Kit for Cell Cycle Analysis. MCF-7 cells were seeded at 8×10^4^ and incubated at 37°C in 5% CO_2_ overnight. After treatment with the tested compound 10 for 24 h, cell pellets were collected and centrifuged (300 g, 5 min). For cell cycle analysis, cell pellets were fixed with 70% ethanol on ice for 15 min and collected again. The pellets were incubated with propidium iodide (PI) staining solution at room temperature for 1 h and analyzed by a Gallios flow cytometer (Beckman Coulter, Brea, CA, USA). Apoptosis detection was carried out by FITC AnnexinV/PI commercial kit (Becton Dickenson, Franklin Lakes, NJ, USA) following the manufacturer protocol. The samples were analyzed by fluorescence-activated cell sorting (FACS) with a Gallios flow cytometer (Beckman Coulter, Brea, CA, USA) within 1 h after staining. Data were analyzed using Kaluza v 1.2 (Beckman Coulter).

- - 1. **Estimation the levels of p53, Bax and Bcl-2**

The magnitudes of the apoptotic proteins; BAX and Bcl-2 were evaluated using BIORAD iScript TM One-Step RT-PCR kit with SYBR^®^Green (BIO-RAD Laboratories, Hercules, CA, US). The method of the applied kit was conducted according to the manufacturer’s protocol and previously reported method. First, mRNA isolation is done using RNeasy extraction kit, up to 1 × 10^7^ cells, depending on the cell line. Then Cells were exposed to RNeasy Lysis Buffer (RLT buffer) and homogenized, while ethanol was then supplemented to the mixture to allow selective binding of RNA to the RNeasy membrane. Afterwards, the sample was loaded to the RNeasy mini spin column where total RNA was bound to the membrane while contaminants competently passed through, and high-quality RNA was eluted in RNase-free water. Then, the reaction mixture was incubated in a real-time thermal detection system (Rotorgene) as follows: cDNA synthesis: 10 min at 50 ◦C, iScript reverse transcriptase inactivation: 5 min at 95 ◦C, polymerase chain reaction (PCR) cycling and detection (30–45 cycles): 10s at 95 ^◦^C and 30 s at 55 ^◦^C to 60 ^◦^C (data collection step) and melt curve analysis: 1 min at 95 ◦C and 1 min at 55 ^◦^C and 10s at 55 ^◦^C (80 cycles, increasing each by 0.5 ^◦^C each cycle).

Human p53 present in MCF-7 cells was determined; using Human p53 ELISA-Kit (CS0070 Sigma) read using spectrophotometer at 450 nm against untreated control cells (negative control) applying the standard protocols of the manufacturers. The samples or standard having human p53 bind to antibodies adsorbed to the microwells. Addition of biotin-conjugated was followed by incubation and addition of dispense of unbound biotin-conjugated streptavidin HRP. Then, the reaction was terminated by adding acid, and the absorbance was measured at 450 nm.

- 1. **Molecular docking study**

The crystal structure of EGFR with its native ligand, erlotinib was downloaded from the protein data bank (PDB code: 1M17). Docking attempts were conducted using Auto Dock Vina 4.2, which requires that both the receptor and the ligands be in PDBT format. Ligand/protein files, grid, and docking parameters were prepared according to previous reports. A 3D grid box of 60 × 60 × 60 Å size (x, y, z) with the spacing of 0.375 Å centered at 96.86, 62.55, and 19.13 Å for docking into the HPIMPD model and at 195.31, 166.19 and 248.78 Å for docking into EGFR. Before docking, M.G.L tools were necessary to synthesize enzyme, co-crystalized ligand, and three lead compounds into the correct format. The calculated RMSD between the docked and co-crystalized ligand, erlotinib was 0.81 Å after re-docking the co-crystalized ligand into the enzyme, indicating that the docking procedure was valid. The Discovery Studio 4.5 visualizer was used to visualize the docking results. For each docked compound, the docking process generated ten poses; the one with the highest affinity and the best docking score was selected.

- 1. **Quantum chemical calculations study**

Density Functional Theory (DFT) computations were performed using the B3LYP functional, a hybrid exchange-correlation functional that combines the gradient-corrected correlation functional of Lee, Yang, and Parr (LYP) with Becke's three-parameter exchange functional. By addressing integration concerns, this method has advantages over pure DFT techniques.

Gaussian 09 was used to fully optimize all molecular geometries at the B3LYP/6-311G++(d,p) level of theory. The energy gap (ΔE), global electrophilicity (ω), softness (σ), electronegativity (χ), hardness (η), and ionization potential (I) were then determined using frontier molecular orbital (FMO)analysis.

Using the optimized geometries, calculations were carried out at the B3LYP/6-31G level of theory in order to visualize the molecular electrostatic potential (MEP). The MEP maps reveal information about the molecules' electrophilic and nucleophilic areas.

**Tables and figures**

**Table S1.** The percentage cytotoxicity of quinazolinones **1**–**10** upon human tumor HepG-2, HCT-116, MCF-7 and normal WI-38 cell lines at different concentrations according to the MTT assay.

| **Conc.(µM)** | **% inhibition** | | | |
| --- | --- | --- | --- | --- |
|  | **HepG-2** | **HCT-116** | **MCF-7** | **WI-38** |
| **Erlotinib** | | | | |
| **1.56** | 6.3 | 7.1 | 6.2 | 7.8 |
| **3.125** | 11.2 | 13.9 | 10.9 | 14.5 |
| **6.25** | 14.1 | 18.7 | 14.3 | 21.2 |
| **12.5** | 28.3 | 31.4 | 26.9 | 34.0 |
| **25** | 45.8 | 47.9 | 41.5 | 50.3 |
| **50** | 57.6 | 60.5 | 58.4 | 64.9 |
| **100** | 71.2 | 73.8 | 69.1 | 87.4 |
| **1** | | | | |
| **1.56** | 18.9 | 20.4 | 13.1 | 46.1 |
| **3.125** | 25.4 | 32.7 | 19.8 | 53.7 |
| **6.25** | 33.5 | 42.2 | 27.4 | 87.3 |
| **12.5** | 42.3 | 53.2 | 38.5 | 98.4 |
| **25** | 65.4 | 73.5 | 64.3 | 100 |
| **50** | 84.9 | 92.3 | 80.1 | 100 |
| **100** | 100 | 100 | 96.3 | 100 |
| **2** | | | | |
| **1.56** | 36.4 | 47.3 | 38.5 | 30.4 |
| **3.125** | 48.1 | 59.4 | 52.4 | 42.9 |
| **6.25** | 63.5 | 71.8 | 66.1 | 54.7 |
| **12.5** | 72.8 | 87.2 | 73.3 | 72.5 |
| **25** | 89.2 | 99.1 | 95.2 | 89.2 |
| **50** | 100 | 100 | 100 | 99.6 |
| **100** | 100 | 100 | 100 | 100 |
| **3a** | | | | |
| **1.56** | 23.4 | 29.3 | 30.1 | 40.9 |
| **3.125** | 33.9 | 43.1 | 44.6 | 59.8 |
| **6.25** | 46.5 | 52.6 | 58.0 | 71.4 |
| **12.5** | 56.3 | 66.4 | 64.1 | 84.7 |
| **25** | 71.7 | 87.9 | 97.3 | 99.2 |
| **50** | 90.6 | 99.6 | 100 | 100 |
| **100** | 100 | 100 | 100 | 100 |
| **3b** | | | | |
| **1.56** | 40.9 | 47.3 | 43.4 | 26.5 |
| **3.125** | 51.3 | 61.3 | 57.9 | 35.3 |
| **6.25** | 63.2 | 78.4 | 73.1 | 50.6 |
| **12.5** | 78.8 | 89.7 | 85.1 | 68.2 |
| **25** | 94.1 | 98.8 | 99.2 | 82.5 |
| **50** | 100 | 100 | 100 | 95.8 |
| **100** | 100 | 100 | 100 | 100 |
| **3c** | | | | |
| **1.56** | 44.2 | 51.9 | 24.1 | 38.7 |
| **3.125** | 55.4 | 70.5 | 37.9 | 51.2 |
| **6.25** | 72.4 | 87.8 | 48.2 | 63.9 |
| **12.5** | 85.5 | 95.3 | 54.9 | 74.3 |
| **25** | 97.7 | 100 | 71.9 | 95.4 |
| **50** | 100 | 100 | 93.3 | 100 |
| **100** | 100 | 100 | 100 | 100 |
| **3d** | | | | |
| **1.56** | 18.3 | 23.7 | 18.9 | 54.9 |
| **3.125** | 28.7 | 34.2 | 25.7 | 66.2 |
| **6.25** | 43.7 | 52.4 | 37.4 | 78.9 |
| **12.5** | 52.3 | 64.1 | 51.6 | 93.4 |
| **25** | 74.1 | 75.2 | 69.8 | 100 |
| **50** | 93.6 | 90.9 | 87.1 | 100 |
| **100** | 100 | 100 | 100 | 100 |
| **4** | | | | |
| **1.56** | 43.8 | 48.3 | 42.7 | 33.1 |
| **3.125** | 55.4 | 64.6 | 54.1 | 45.3 |
| **6.25** | 63.9 | 76.3 | 65.3 | 63.4 |
| **12.5** | 85.1 | 93.1 | 80.2 | 76.5 |
| **25** | 96.2 | 100 | 93.9 | 94.9 |
| **50** | 100 | 100 | 100 | 100 |
| **100** | 100 | 100 | 100 | 100 |
| **5** | | | | |
| **1.56** | 30.4 | 41.2 | 35.2 | 20.4 |
| **3.125** | 41.3 | 58.3 | 49.3 | 28.9 |
| **6.25** | 61.6 | 69.3 | 65.2 | 39.1 |
| **12.5** | 71.9 | 80.4 | 77.6 | 51.3 |
| **25** | 86.5 | 98.5 | 93.9 | 72.7 |
| **50** | 99.7 | 100 | 100 | 92.4 |
| **100** | 100 | 100 | 100 | 100 |
| **6a** | | | | |
| **1.56** | 28.2 | 39.2 | 29.1 | 47.9 |
| **3.125** | 42.3 | 48.7 | 41.3 | 63.4 |
| **6.25** | 57.1 | 63.4 | 53.4 | 74.9 |
| **12.5** | 72.3 | 79.3 | 69.5 | 94.1 |
| **25** | 81.9 | 97.5 | 82.6 | 100 |
| **50** | 100 | 100 | 100 | 100 |
| **100** | 100 | 100 | 100 | 100 |
| **6b** | | | | |
| **1.56** | 7.9 | 8.4 | 5.2 | 33.9 |
| **3.125** | 12.3 | 17.1 | 10.4 | 45.2 |
| **6.25** | 20.5 | 25.3 | 17.1 | 58.5 |
| **12.5** | 32.3 | 33.9 | 23.8 | 69.7 |
| **25** | 54.2 | 55.9 | 38.1 | 91.4 |
| **50** | 61.8 | 71.4 | 58.1 | 100 |
| **100** | 73.5 | 88.3 | 68.3 | 100 |
| **7** | | | | |
| **1.56** | 23.6 | 34.6 | 34.5 | 52.3 |
| **3.125** | 37.3 | 49.1 | 49.2 | 69.9 |
| **6.25** | 52.1 | 64.3 | 60.3 | 79.1 |
| **12.5** | 65.2 | 76.7 | 73.5 | 96.5 |
| **25** | 78.4 | 96.1 | 89.6 | 100 |
| **50** | 96.3 | 100 | 100 | 100 |
| **100** | 100 | 100 | 100 | 100 |
| **8a** | | | | |
| **1.56** | 7.4 | 37.1 | 9.2 | 39.7 |
| **3.125** | 20.5 | 53.8 | 16.8 | 53.6 |
| **6.25** | 22.2 | 69.2 | 24.9 | 73.1 |
| **12.5** | 39.1 | 78.3 | 32.7 | 94.5 |
| **25** | 57.3 | 93.8 | 61.3 | 100 |
| **50** | 85.1 | 100 | 70.5 | 100 |
| **100** | 97.5 | 100 | 89.2 | 100 |
| **8b** | | | | |
| **1.56** | 46.1 | 51.4 | 46.7 | 26.3 |
| **3.125** | 63.4 | 63.1 | 56.4 | 38.4 |
| **6.25** | 72.3 | 76.5 | 76.5 | 50.2 |
| **12.5** | 87.1 | 93.4 | 88.7 | 61.3 |
| **25** | 98.5 | 100 | 100 | 75.4 |
| **50** | 100 | 100 | 100 | 93.8 |
| **100** | 100 | 100 | 100 | 100 |
| **9** | | | | |
| **1.56** | 33.2 | 44.9 | 40.3 | 33.5 |
| **3.125** | 45.8 | 59.1 | 53.6 | 44.6 |
| **6.25** | 63.4 | 72.3 | 65.2 | 56.1 |
| **12.5** | 70.6 | 85.2 | 76.8 | 70.5 |
| **25** | 95.1 | 96.4 | 92.5 | 91.3 |
| **50** | 100 | 100 | 100 | 100 |
| **100** | 100 | 100 | 100 | 100 |
| **10** | | | | |
| **1.56** | 9.1 | 12.4 | 7.9 | 27.7 |
| **3.125** | 17.2 | 21.7 | 14.5 | 42.3 |
| **6.25** | 26.4 | 29.9 | 22.2 | 56.5 |
| **12.5** | 36.5 | 41.3 | 32.4 | 70.7 |
| **25** | 61.3 | 66.5 | 50.7 | 83.9 |
| **50** | 69.5 | 80.4 | 65.5 | 100 |
| **100** | 90.1 | 98.1 | 82.7 | 100 |

**Table S2.** Cell cycle analysis after 48 h incubation with compound **6b**

| **Compound No.** | **%G0-G1** | **%S** | **%G2/M** |
| --- | --- | --- | --- |
| **6b** /**MCF-7** | 46.68 | 18.03 | 35.29 |
| **Cont./MCF-7** | 69.25 | 23.94 | 6.81 |

**Table S3.** Apoptosis induction analysis within MCF-7 cells treated with compound **6b**

|  | Apoptosis | | | Necrosis |
| --- | --- | --- | --- | --- |
|  | **Total** | **Early** | **Late** |  |
| 6b/ MCF-7 | 29.76 | 8.92 | 15.39 | 5.45 |
| Cont. / MCF-7 | 2.37 | 0.35 | 0.18 | 1.84 |

**Table S4.** HOMO, LUMO, and ESP energies for the compounds.

| Com. No. | Quantum diagram |
| --- | --- |
| **1** | 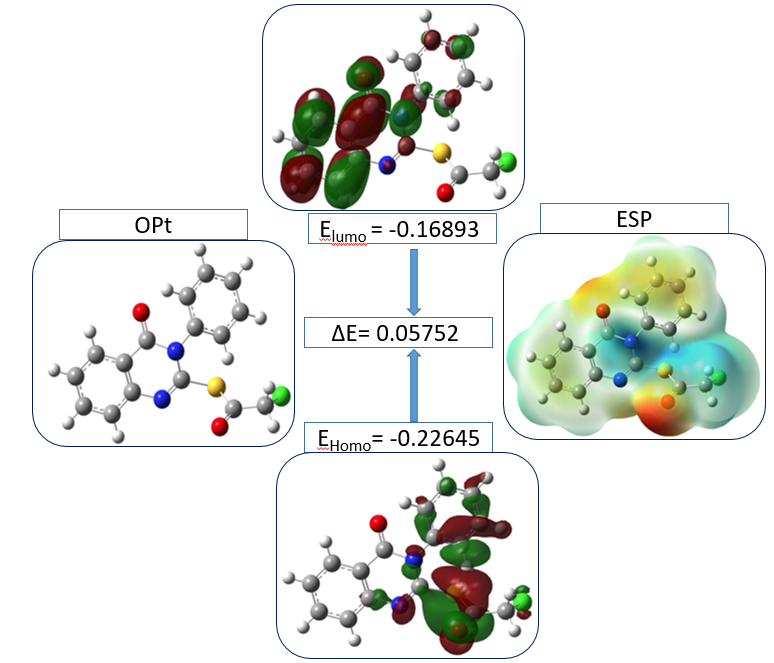 |
| **2** | 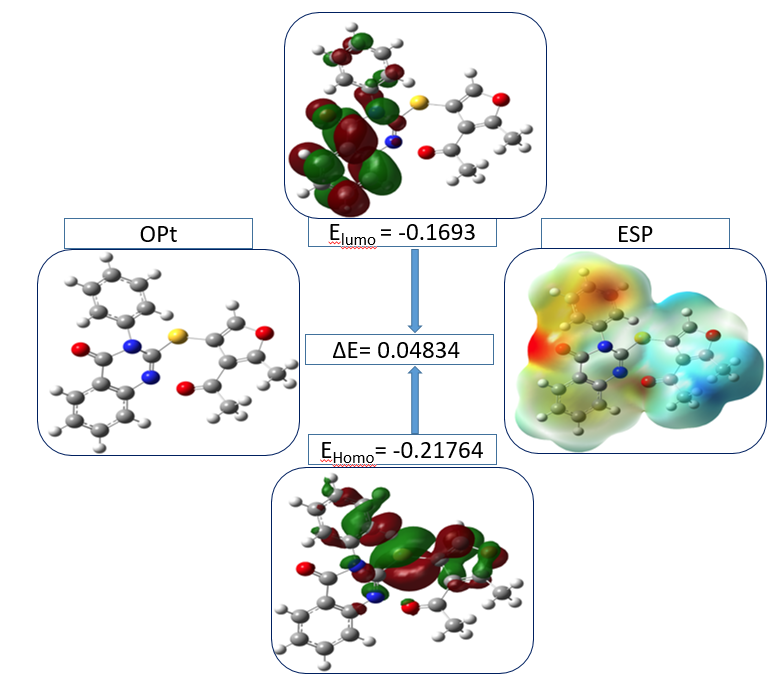 |
| **3a**  **3b**  **3c**  **3d** | 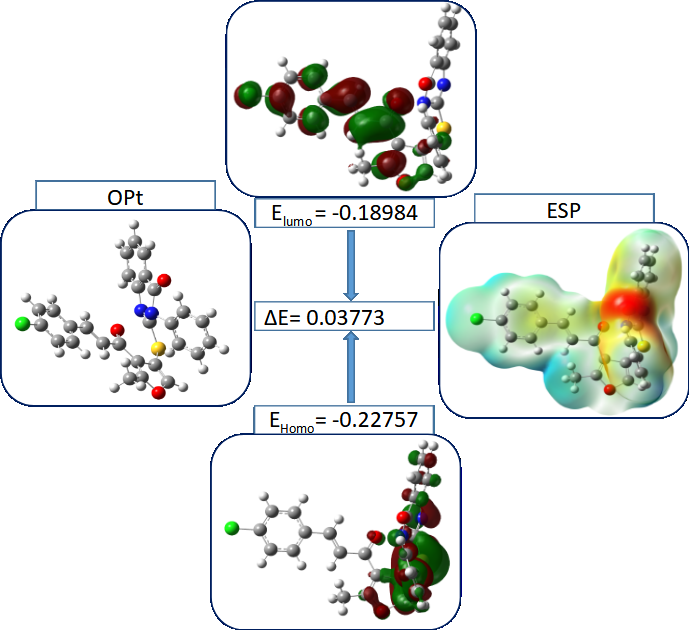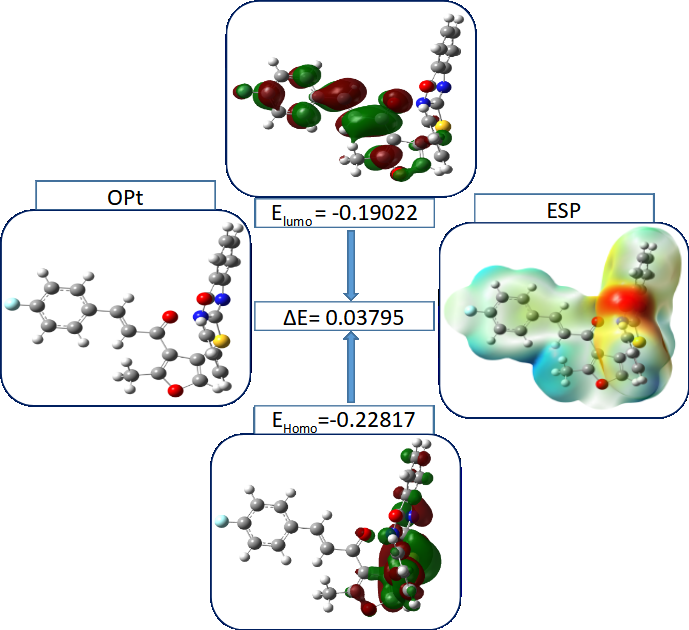  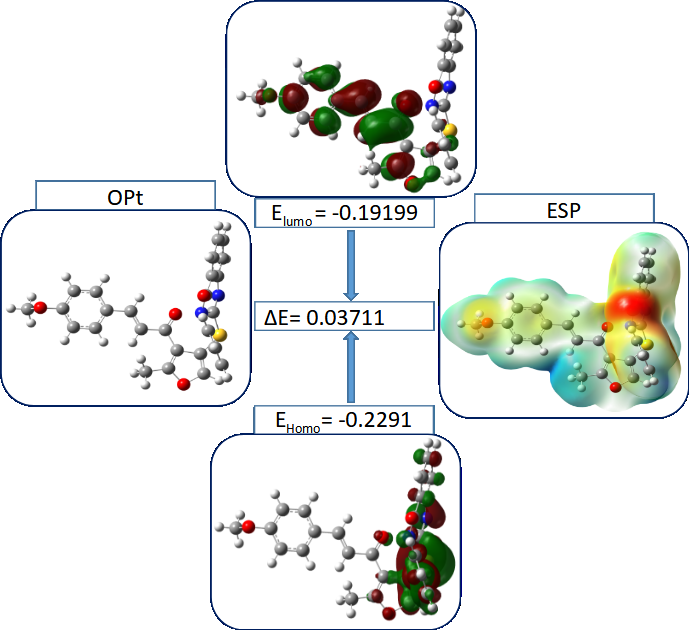  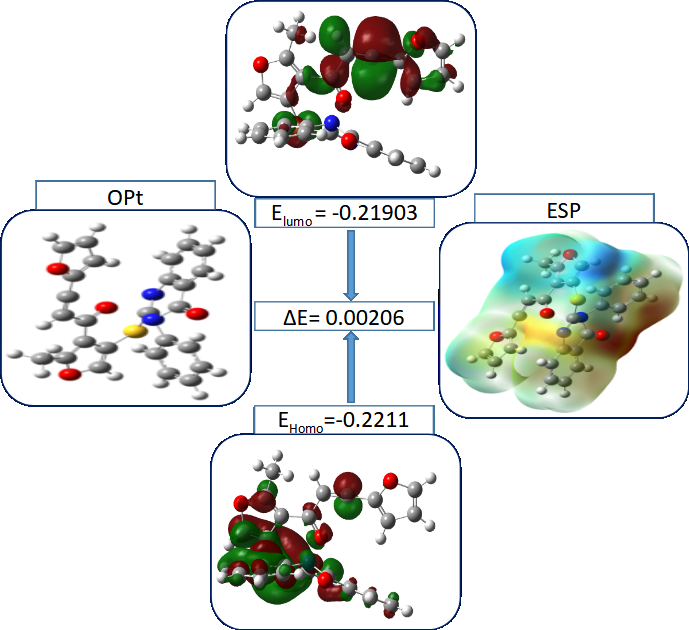 |
|  |  |
|  |  |
| **4**  **5** | 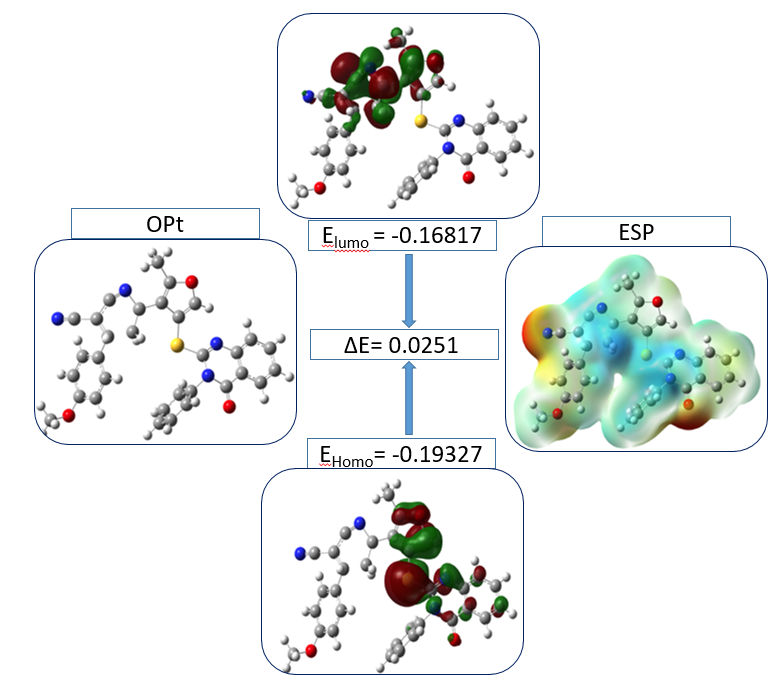  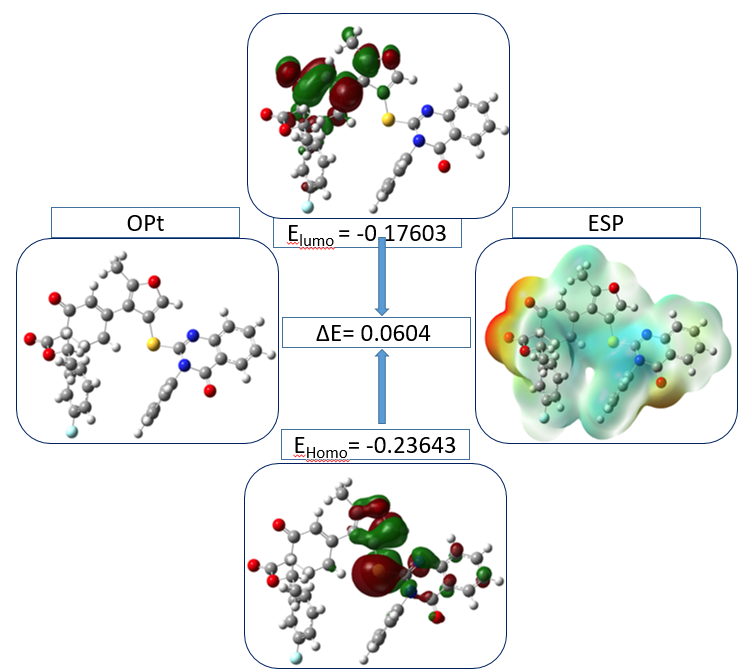 |
|  |  |
| **6a** | 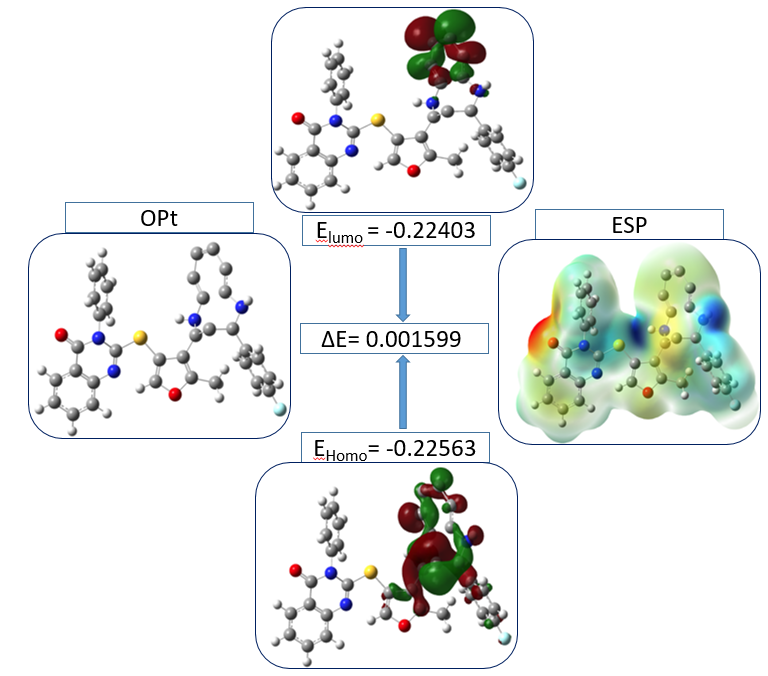 |
| **6b** | 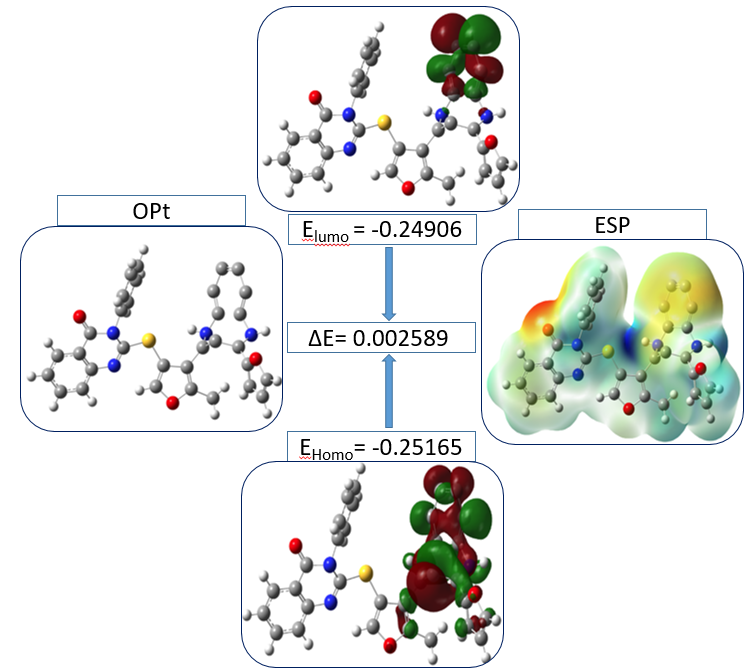 |
| **7** | 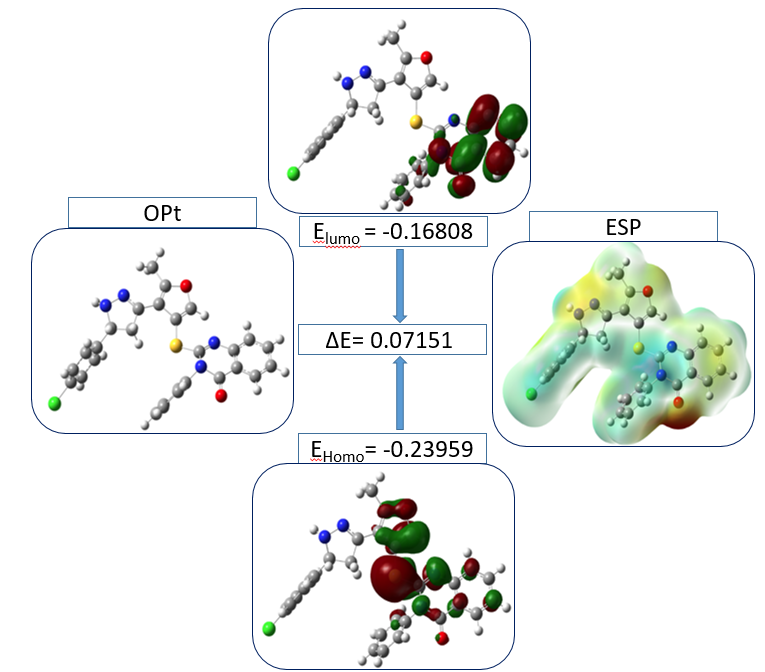 |
| **8a** | 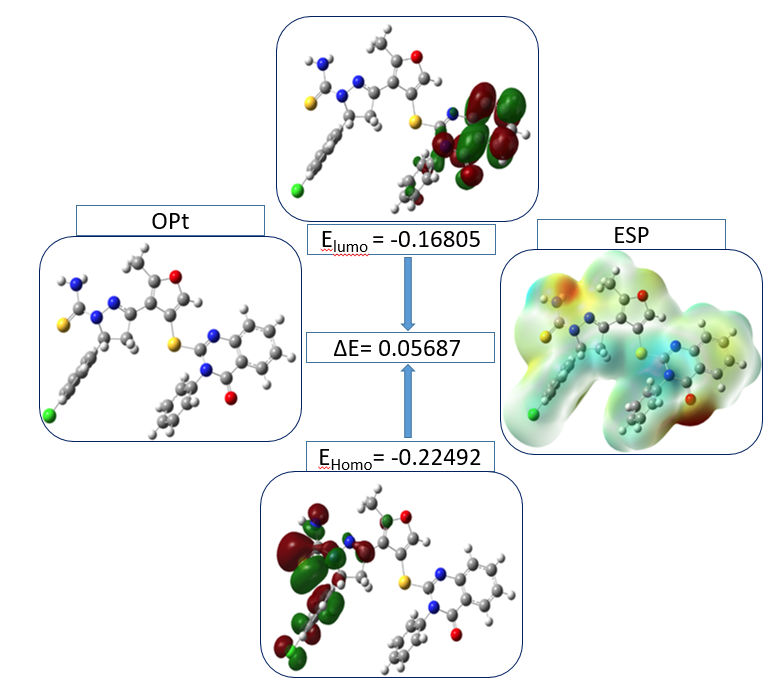 |
| **8b** | 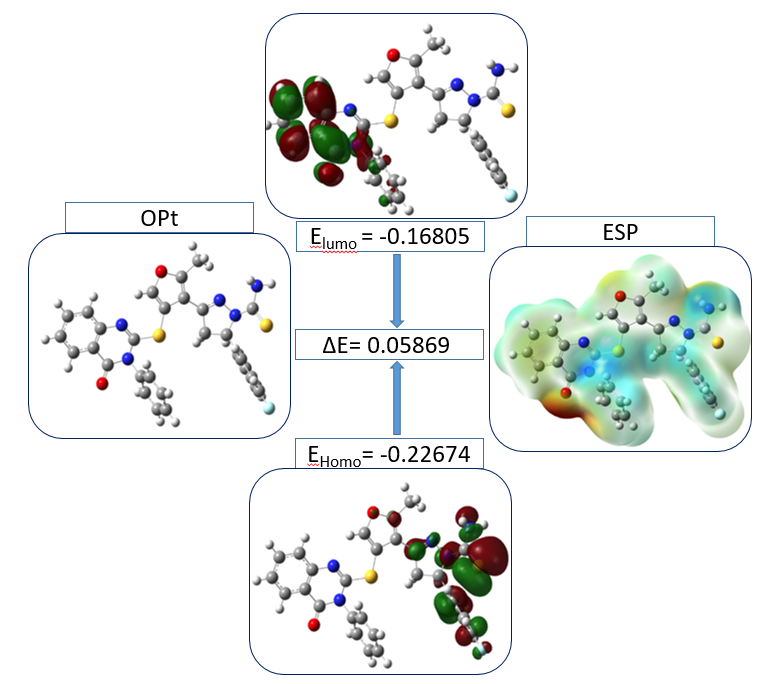 |
| **9** | 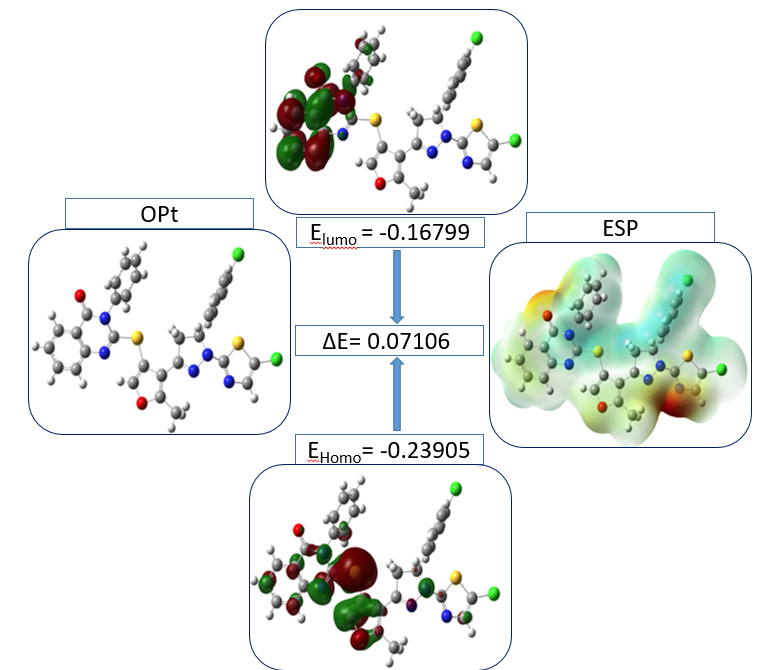 |
| **10** | 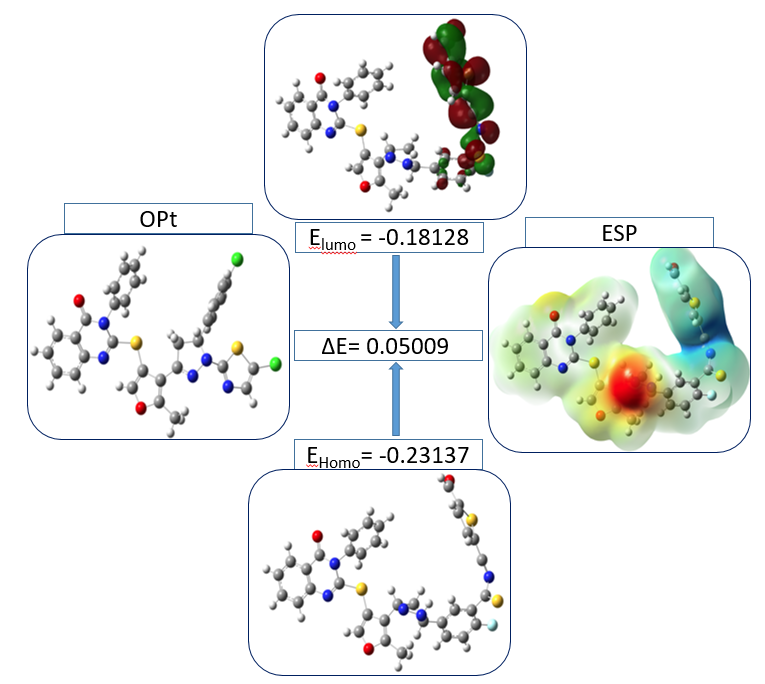 |

**
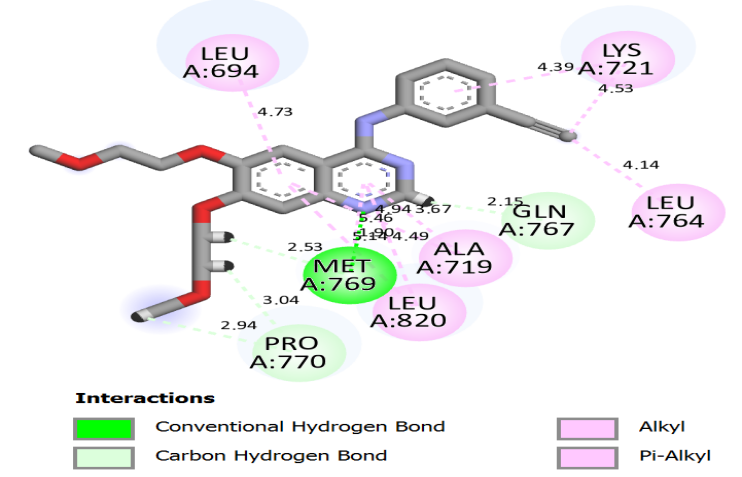
**

**
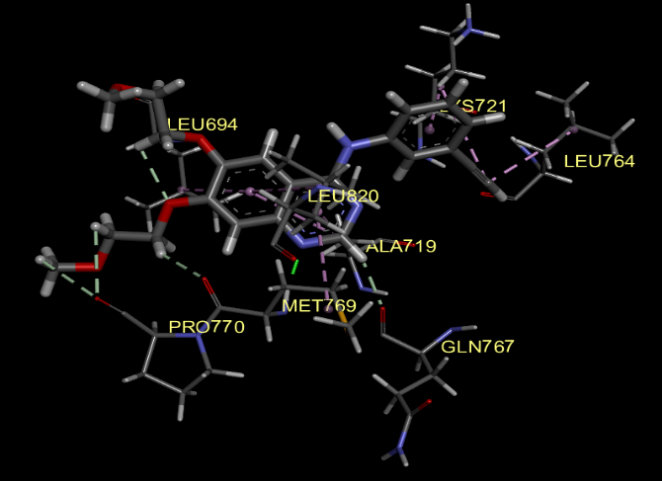
**

**Figure S50. A** & **B** diagrams illustrated 2D and 3D binding features of the original ligand erlotinib within the active site of EGFR (PDB code: 1M17).
